# Future projection of cancer patients with cardiovascular disease in Japan by the year 2039: a pilot study

**DOI:** 10.1007/s10147-019-01426-w

**Published:** 2019-03-22

**Authors:** Yuji Okura, Tsugumi Takayama, Kazuyuki Ozaki, Hiroshi Tanaka, Akira Kikuchi, Toshihiro Saito, Toshiki Tanigawa, Yasumasa Takii, Hiroshi Seki, Tatsuya Takenouchi, Takaaki Chou, Nobuaki Sato, Naohito Tanabe, Tohru Minamino

**Affiliations:** 1 Department of Onco-cardiology, Niigata Cancer Center Hospital, Chu-o-ku Kawagishicho 2-15-3, Niigata, 951-8560 Japan; 2 Department of Cardiovascular Biology and Medicine, Niigata University Graduate School of Medical and Dental Sciences, Niigata, Japan; 3 Department of Respiratory Medicine, Niigata Cancer Center Hospital, Niigata, Japan; 4 Department of Gynecology, Niigata Cancer Center Hospital, Niigata, Japan; 5 Department of Urology, Niigata Cancer Center Hospital, Niigata, Japan; 6 Department of Gastroenterological Surgery, Niigata Cancer Center Hospital, Niigata, Japan; 7 Department of Diagnostic Radiology, Niigata Cancer Center Hospital, Niigata, Japan; 8 Department of Dermatology, Niigata Cancer Center Hospital, Niigata, Japan; 9 Department of Hematology, Niigata Cancer Center Hospital, Niigata, Japan; 10 Department of Breast Oncology, Niigata Cancer Center Hospital, Niigata, Japan; 11 Department of Health and Nutrition, Faculty of Human Life Studies, University of Niigata Prefecture, Niigata, Japan

**Keywords:** Epidemiology, Cancer, Cardiovascular disease, Comorbidity, Prediction models

## Abstract

**Background:**

The number of cancer patients in Japan is estimated to rise to 3.5 million by 2025. The disease burden may be further complicated by comorbidities caused by cardiovascular disease (CVD). Predicting the number of cancer patients with CVD can help anticipate future resource needs.

**Methods:**

We used statistics derived from the Niigata Cancer Center CVD Study (2015) as well as population estimates from the National Cancer Center’s Cancer Registry and Statistics survey of 2017 for convenience. We simply multiplied the projected number of cancer patients through the year 2039 by the CVD prevalence in 2015, with patients classified by sex, age, and cancer type to estimate the number of cancer patients with CVD.

**Results:**

The total number of Japanese cancer patients with CVD was 253,000 in 2015 and is predicted to increase rapidly by 30,000 in 2020 and peak at 313,000 in 2030–2034. Men will dominate the CVD population at 2.5-fold the number of women. The growth rate of the population with both cancer and CVD will be greater than that of the cancer-only population (1.23 vs 1.18, *P* < 0.001), and will comprise notably high proportions of patients with prostatic, breast, and uterine cancers (1.80, 1.57, and 1.66, *P* < 0.001, respectively).

**Conclusion:**

Future cancer patients will be older and more likely to have CVD. Although men will continue to dominate this population, the increase in the number of women will be pronounced. Cancer care providers should be trained to recognize CVD and provide any necessary interventions concurrently with cancer therapy.

**Electronic supplementary material:**

The online version of this article (10.1007/s10147-019-01426-w) contains supplementary material, which is available to authorized users.

## Introduction

Cancer and cardiovascular disease (CVD) are major public health problems with rising incidence rates in developed countries [1–4]. In 2015, approximately 3.1 million individuals in Japan had cancer, and more than 862,000 new cases are diagnosed annually [3]. Separately, the number of outpatients with left ventricular dysfunction (LVD) was 979,000 in 2005 and is predicted to increase gradually as the population ages, reaching 1.3 million by 2030 [4].

CVD is endemic among cancer patients; hypertension, atrial fibrillation (AF), ischemic heart disease (IHD), venous thromboembolism (VTE), LVD, and heart failure (HF) are common comorbidities [5, 6]. This is attributable to common risk factors shared by cancer and CVD (e.g., aging, smoking, diabetes, obesity, and physical inactivity), neoplastic effects on the cardiovascular system, chemotherapy- and radiotherapy-induced cardiovascular toxicities, and longer life expectancies [1, 3, 6–9].

CVD-related comorbidities are a persistent burden on cancer patients [5–7]. CVD causes acute HF, myocardial infarction, pulmonary thromboembolism, stroke, cardiogenic shock, syncope, and arrhythmia; these can be lethal or lead to serious disabilities. CVDs often relapse, and some cause major adverse cardiovascular events (MACEs); they also often interrupt the patient’s cancer treatment. Some anticancer drugs are contraindicated to avoid CVD and MACE, resulting in insufficient treatment and a worse prognosis. For example, anthracycline and trastuzumab are withheld for LVD patients, while bevacizumab is withheld for VTE patients. Curative surgery may be abandoned in favor of palliative surgery or radiotherapy. In our registry, the 5-year survival rate was 64.0% for all cancer patients and 44.2% for cancer patients with CVD [6]. Comorbidities with AF, VTE, and N-terminal prohormone of brain natriuretic peptide (NT-proBNP) elevation were significantly associated with mortality [6].

The number of cancer patients in Japan is estimated to increase to 3.5 million by 2025 [3]. The future disease burden may be further complicated by comorbidities associated with CVD and may vary among cancer types. Predicting the CVD epidemic can help to anticipate future resource needs among cancer-treating institutions. However, to our knowledge, no predictive studies have been performed on this issue in Japan. Therefore, we conducted a retrospective study at our cancer center hospital to predict the impact of CVD on cancer patients over the next 20 years.

## Methods

### Study setting and design

To predict the future number of cancer patients with CVD, we utilized the projected number of cancer patients by 2039 as determined by national cancer statistics (Supplementary Table 1) [3]. The CVD prevalence in 2015 was determined by examining our institutional cancer registry, in which we identified all cancer patients with CVD over a 10-year period (2005–2014), as previously described [6]. We also estimated CVD prevalence as correlated with age, sex, and cancer type in 2015 (Supplementary Table 2), and then extrapolated these correlations to project the number of cancer patients in each age and sex group up to 2039 [3] and to estimate the number of cancer patients with CVD.

### Institutional cancer database

Our hospital maintains a cancer registry that is regularly updated with the clinical information and vital statuses of patients. The dates of initial diagnosis, clinical and pathological diagnosis, and death were obtained from the registry [6, 9].

### Selection of patients with CVD

Systemic screening at our hospital is performed before cancer therapy, as previously described [6, 9]. All patients underwent a physical examination, 95% submitted to electrocardiograms, and 53% underwent chest radiographies. When necessary, cancer patients were referred to cardiologists; 23% and 7% of the patients underwent transthoracic echocardiography and vascular echo, respectively. Nearly all of the patients (98%) underwent computed tomography (CT) to detect tumors in the chest, abdomen, or pelvis. NT-proBNP levels were measured for screening or diagnosis of HF beginning in 2008. These data were double-checked and stored in our database. Only the first image or record was analyzed for patients with multiple imaging studies or laboratory tests that satisfied our inclusion criteria. Moreover, the maximum values of NT-proBNP obtained for each patient were selected for analysis.

### Definition and diagnosis of CVD

We focused on LVD, AF, IHD, aortic stenosis (AS), VTE, and significant elevation of NT-proBNP because these conditions constitute a burden on cancer patients seeing as they require sustained surveillance or medication. Although hypertension was the most common CVD in both cancer and non-cancer patients, we did not focus on it owing to its wide prevalence and relatively successful treatability. The definitions of CVDs used were the same as those described previously, as follows [6]: LVD was divided into systolic dysfunction (LVSD) and diastolic dysfunction (LVDD) exclusively, as determined by echocardiography. LVSD was diagnosed based on a left ventricular ejection fraction (LVEF) ≤ 50%, while LVDD was diagnosed based on an ejection fraction > 50% and early transmitral flow velocity/early diastolic velocity of the mitral annulus > 8 plus at least 2 of the following criteria: (1) left atrial enlargement, (2) left ventricular hypertrophy, and (3) AF [9]. AF was diagnosed using 12-lead electrocardiography. IHD was identified if the patient had at least 2 of the following: (1) documented history of IHD (myocardial infarction, angina, or prior coronary revascularization); (2) medication for IHD, and (3) presence of IHD following any cardiac tests or imaging. AS was classified as moderate or severe depending on the echocardiography findings [9]. VTE was diagnosed if proximal deep venous thrombosis (DVT) or pulmonary thromboembolism were present. VTE was diagnosed with contrast-enhanced CT by radiologists. DVT of the legs was also diagnosed with vascular echo. We included all pulmonary thromboemboli and central types of DVT (superior vena cava, inferior vena cava, subclavian, iliac, femoral, and popliteal DVT); however, we excluded DVT of the lower legs. NT-proBNP > 900 pg/mL was considered a significant elevation based on the Japanese Heart Failure Society statement and international standards [10, 11]. CVD was defined as any of LVSD, LVDD, AF, IHD, AS, VTE, or elevation of NT-proBNP > 900 pg/mL that were documented anytime during the clinical course.

### Prevalence of CVD according to cancer type

We determined the number of cancer patients who had CVD between 2005 and 2014 and who survived until January 2015. The proportion of CVD patients among cancer survivors in 2015 was considered the prevalence of CVD in the present study (Supplementary Table 2) [6]. If any patient was diagnosed with any other cancers 2 or more times during the recruitment period and survived until January 2015, CVD was counted for each cancer type to determine the cancer type-specific CVD prevalence. Cancer types were categorized in accordance with International Classification of Diseases, 10th revision (Supplementary Table 1–3). There were too few patients with male breast cancer (*n* = 4) and brain cancer (*n* = 5) to evaluate CVD prevalence. Other cancers and unspecified primary sites were not included in the study.

### Projected number of cancer patients with CVD per each cancer type

We applied the CVD prevalence reported in 2015 [6] to the projected number of cancer patients in each age and sex group until the year 2039 [3] to provide a prospective estimate of the number of cancer patients with CVD. The projected number of cancer patients [3] was calculated using the formula reported by Pisani et al. [12] with incidence rates and survival probabilities in Japan [13]. To predict the total number of cancer patients with each type of CVD, we used a two-step process as follows. Using AF as an example, we first determined the subtotals of the cancer patients with AF for each cancer type. Second, we summed the subtotals of each aforementioned cancer type and presented the sums as the predicted total number of cancer patients with AF. These steps were repeated for each type of CVD within each time period.

### Statistical analysis

The proportions of cancer with each CVD were compared between 2015–2019 and 2035–2039 using *χ*^2^ test. The change in the number of patients with each cancer type from 2015–2019 to 2035–2039 (growth rate) was calculated using the following equation:

$${\text{Number of cancer patients in 2}}0{\text{35}}{-}{\text{2}}0{\text{39}}/{\text{number of cancer patients in 2}}0{\text{15}}{-}{\text{2}}0{\text{19}}.$$

The odds ratio of comorbidity of CVD in 2035–2039 in comparison with 2015–2019 was calculated for each cancer. Data were analyzed with IBM SPSS statistics version 25.0 (Armonk, NY, USA).

### Ethical considerations

The study protocol was reviewed and approved by the Niigata Cancer Center ethics committee. The study was approved as minimal-risk research and informed consent was waived in accordance with the ethical guidelines for epidemiology research in Japan.

## Results

### Prevalence of CVD among cancer patients in 2015

Table 1 shows the prevalence of CVD across 6 age groups. LVSD, LVD, and elevation of NT-proBNP were frequently observed in patients with childhood and adolescent cancer. Generally, the rate of CVD increased progressively with age, reaching 13.1% in male patients and 7.9% in female patients. However, VTE rates were largely unchanged among age groups. AS increased progressively with age and occurred more frequently in female than in male patients. The prevalence data of each type of CVD according to the type of cancer are shown in Supplementary Table 2.

**Table 1:** Prevalence of CVD in all cancer (C00-96) in age groups

Male							
Age groups	0–14	15–44	45–54	55–64	65–74	75-	All
LVSD	3.49	0.51	0.84	0.77	1.53	2.20	1.31
LVDD	0.00	0.25	0.56	1.54	1.91	2.41	1.58
LVD	3.49	0.76	1.40	2.31	3.44	4.61	2.89
AF	1.16	0.25	1.26	2.72	4.93	6.71	3.98
IHD	0.00	0.76	1.54	2.31	3.22	3.46	2.46
AS	0.00	0.00	0.00	0.08	0.48	1.20	0.39
VTE	1.16	1.26	1.26	0.65	1.02	1.31	0.98
NT-proBNP	11.63	0.25	0.56	0.85	1.24	3.20	1.36
CVD	12.79	2.78	5.04	7.14	10.44	13.10	8.73
Female							
Age groups	0–14	15–44	45–54	55–64	65–74	75-	All
LVSD	5.77	0.13	0.19	0.27	0.40	0.81	0.39
LVDD	0.00	0.20	0.19	0.64	1.45	1.94	0.79
LVD	5.77	0.34	0.38	0.91	1.85	2.75	1.18
AF	3.85	0.00	0.13	0.37	2.40	2.91	1.05
IHD	0.00	0.07	0.06	0.46	0.60	0.97	0.40
AS	0.00	0.00	0.00	0.09	0.75	1.78	0.44
VTE	1.92	0.54	0.38	1.37	1.30	1.13	0.95
NT-proBNP	7.69	0.07	0.25	0.32	1.05	2.10	0.72
CVD	19.23	0.87	0.89	2.97	6.00	7.92	3.53

C00-96 indicates cancer types coded by ICD 10. CVD prevalence in all cancers (from 00 to 96) are derived from the Niigata Cancer Center study (2015). Values in each CVD line are percentages

*AF* atrial fibrillation, *AS* aortic stenosis, *CVD* cardiovascular disease, *IHD* ischemic heart disease, *LVD* left ventricular dysfunction, *LVDD* left ventricular diastolic dysfunction, *LVSD* left ventricular systolic dysfunction, *NT-proBNP* N-terminal pro-brain natriuretic peptide, *VTE* venous thromboembolism

### Projection of cancer patients with CVD by 2039

**Fig. 1:**
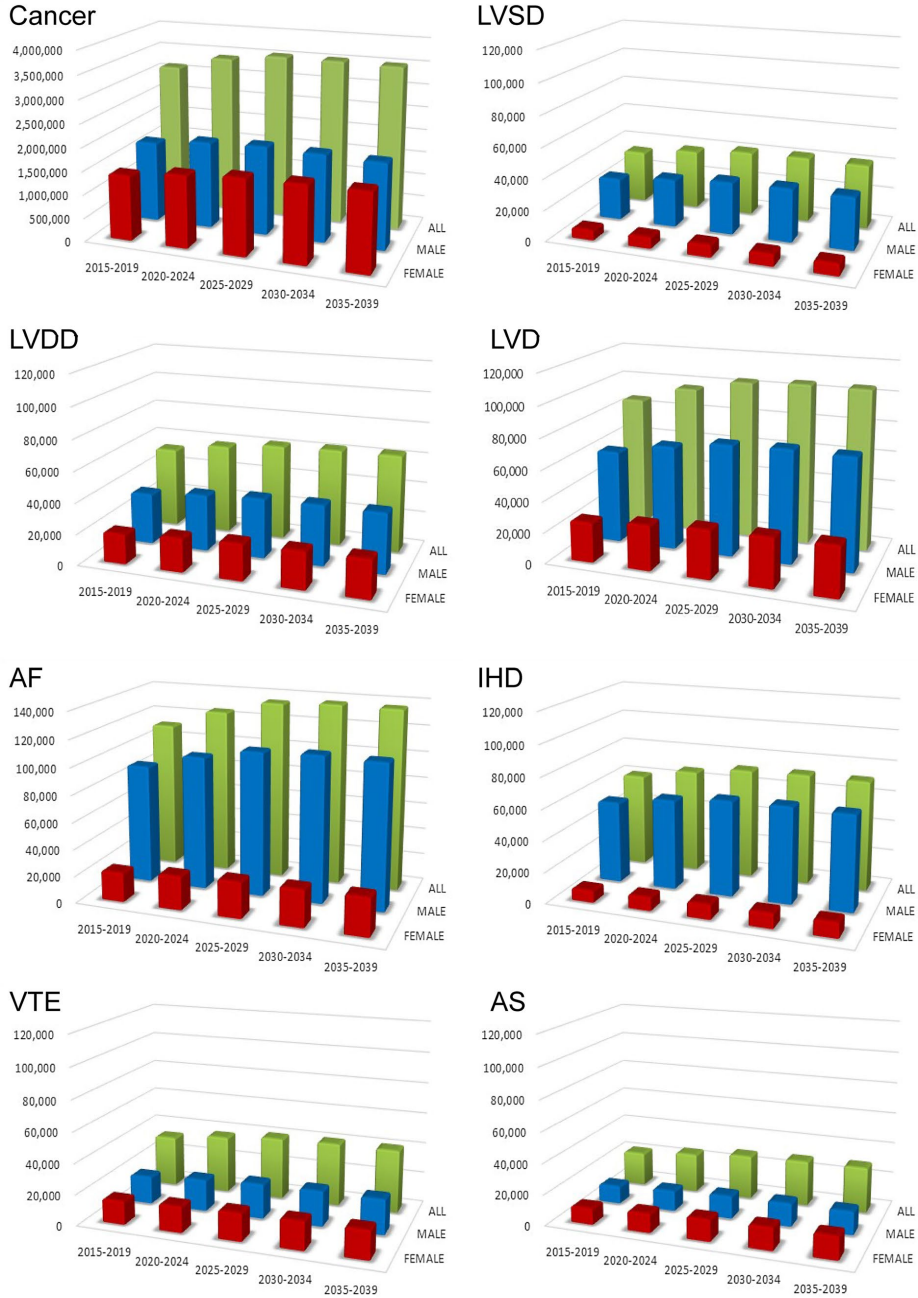

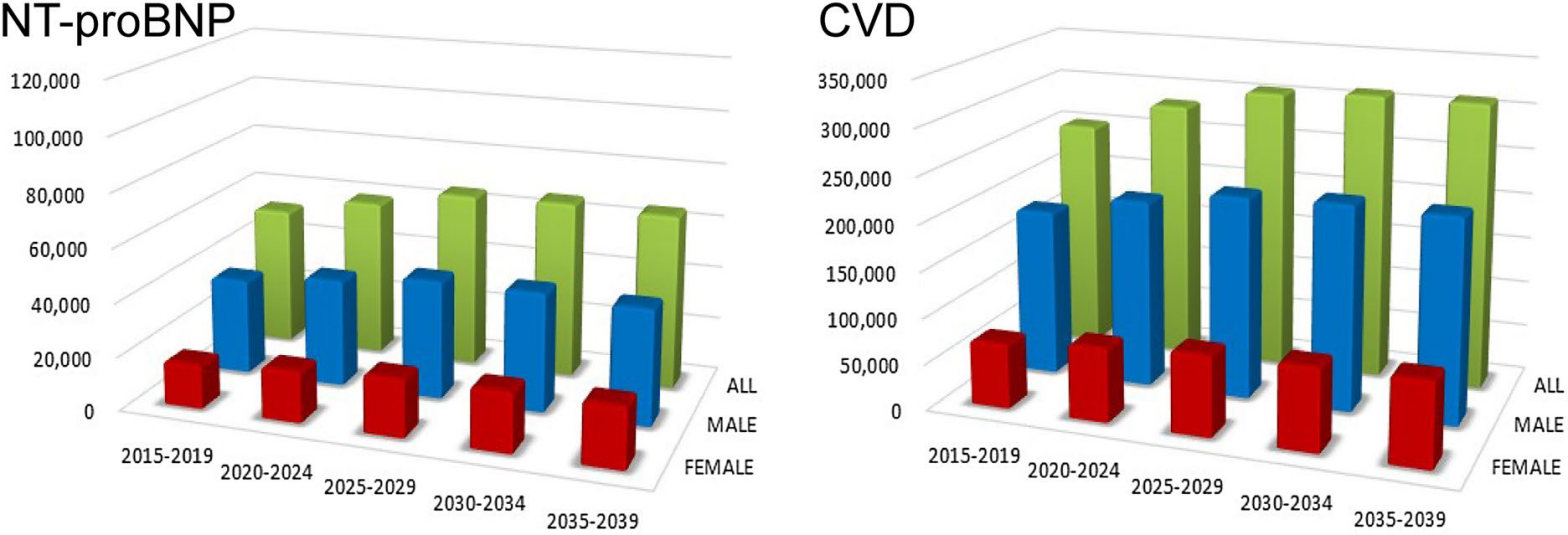
Projected number of cancer patients between 2015 and 2039. Bar graphs show the number of cancer patients in total and with each CVD stratified by 5-year intervals. The numbers of cancer patients are the sum of “all (C00–C96)” lines listed in Supplementary Table 1. The numbers of cancer patients with CVD are listed in the “total” categories of Supplementary Table 3. AF, atrial fibrillation; AS, aortic stenosis; CVD, cardiovascular disease; IHD, ischemic heart disease; LVD, left ventricular dysfunction; LVDD, left ventricular diastolic dysfunction; LVSD, left ventricular systolic dysfunction; NT-proBNP, N-terminal pro-brain natriuretic peptide; VTE, venous thromboembolism

Japanese cancer patients totaled 3.1 million in 2015; their numbers were expected to increase rapidly by 300,000 by 2020 and peak at 3.5 million in 2025–2034 (Fig. 1 and Supplementary Table 1). The total number of Japanese cancer patients with CVD was 253,000 in 2015 and is expected to rise by 30,000 in 2020, peaking at 313,000 in 2030–2034 (Fig. 1 and Supplementary Table 3). Male patients will dominate the CVD population as a whole, at 2.5-fold greater than the female population. Male CVD will peak by the early 2030s; however, female CVD will continue to increase thereafter. AF was the most common CVD in cancer patients in 2015 (111,000 people) and its incidence is expected to peak at 137,000 in 2030. AF in men will be 4 times more prevalent than in women. The majority of cancer patients with AF were over 75 years (52% and 54% of male and female patients, respectively). LVD was the second most common CVD in patients with cancer, numbering 87,000 in 2015; this number is expected to peak at 106,000 in 2030. Most cancer patients with LVD were over 75 years of age (51% and 53% of male and female patients, respectively). IHD was the third most common comorbidity among cancer patients with CVD; a strong male predominance (6.5-fold that of female patients) will be observed in the future. There were 61,000 cancer patients with IHD in 2015; this number is expected to peak at 72,000 in 2030.

### Proportion of cancer types in male CVD patients in 2015 and 2039

**Fig. 2:**
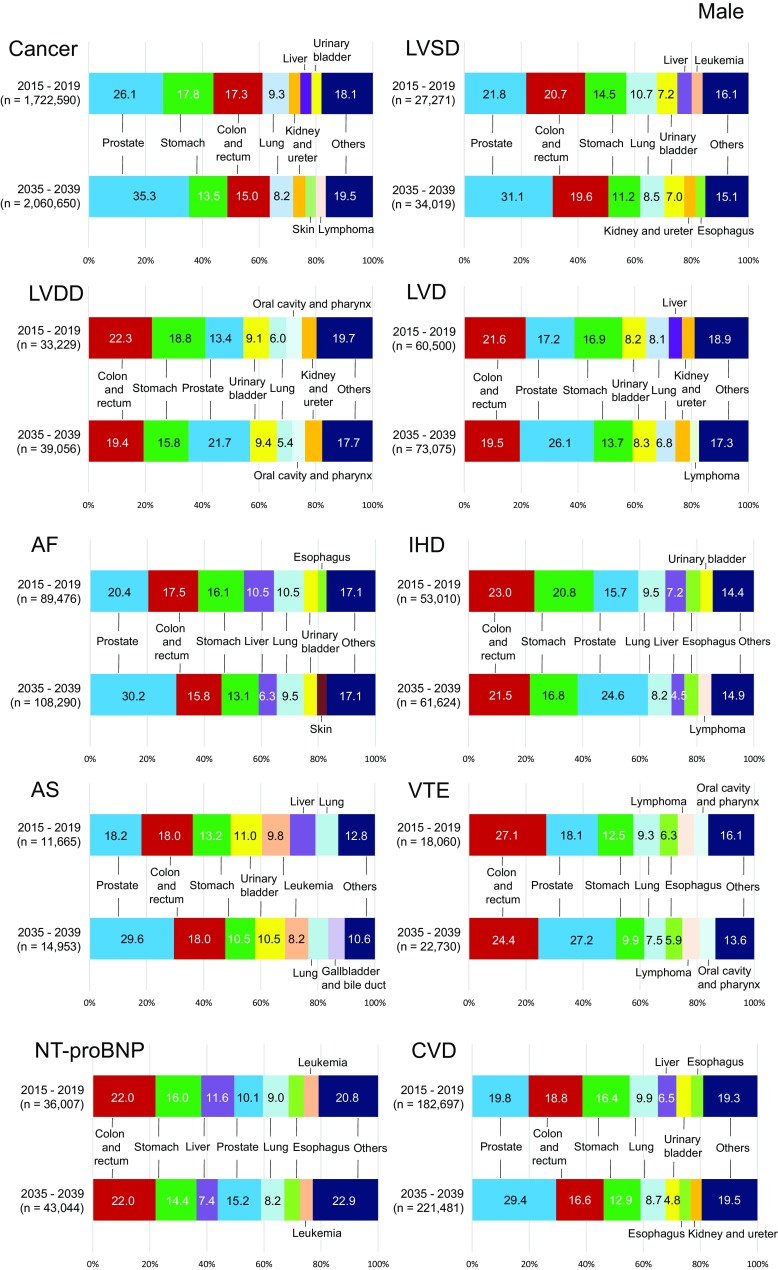

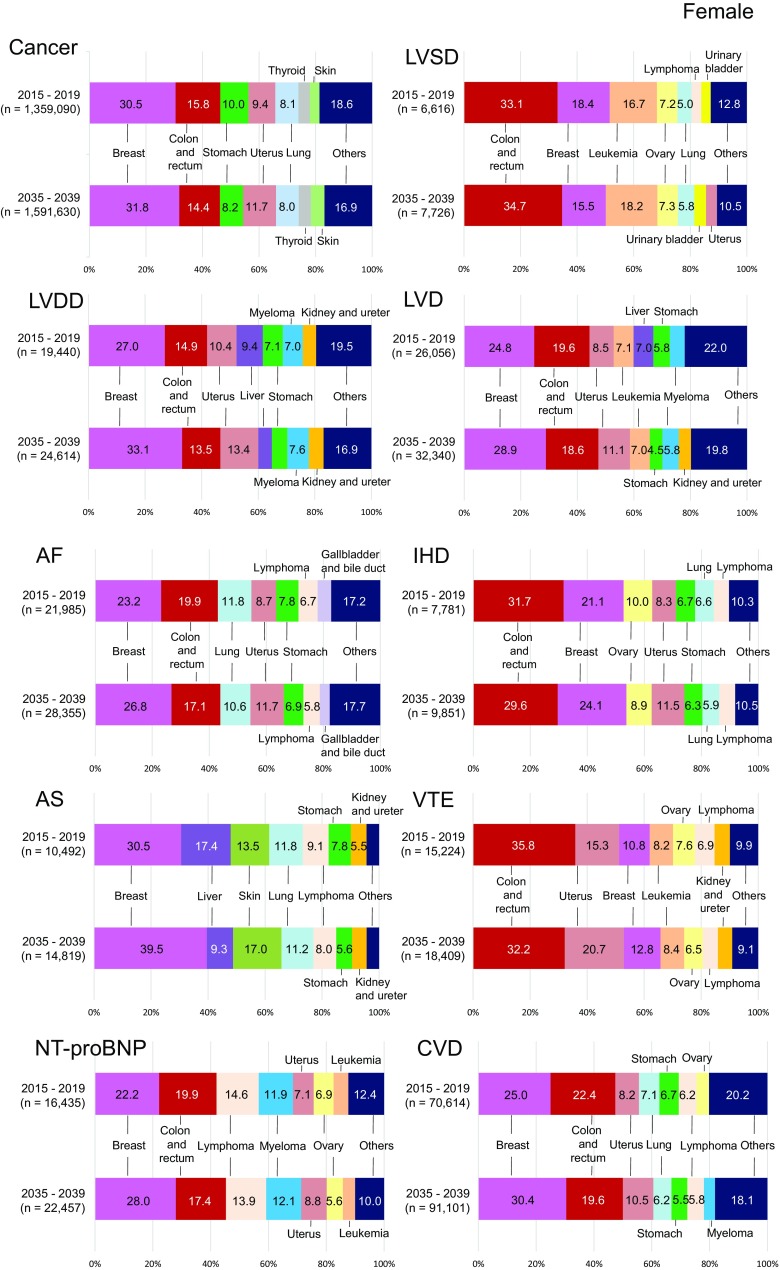
Proportions of the top 7 cancer types in 2015–2019 and 2035–2039 in cancer patients with each type of CVD. The numbers of cancer patients are the sum of each categories from ‘Oral cavity & pharynx’ (C00–C14) to ‘Leukemia’ (C91–C95) listed in Supplementary Table 3. The numbers of cancer patients with CVD are listed in the “total” categories of Supplementary Table 3. According to Japanese national cancer statistics, there were 1.7 million male cancer patients and 1.4 million female cancer patients in 2015; the numbers are expected to be 1.9 million and 1.7 million in 2025, respectively (Supplementary Table 1). To compare the proportion of cancer types, we summed the number of patients with each cancer and considered the total to be the denominator. Accordingly, the number of cancer patients listed here was larger than those of Japanese national cancer statistics. *AF* atrial fibrillation, *AS* aortic stenosis, *CVD* cardiovascular disease, *IHD* ischemic heart disease, *LVD* left ventricular dysfunction, *LVDD* left ventricular diastolic dysfunction, *LVSD* left ventricular systolic dysfunction, *NT-proBNP* N-terminal pro-brain natriuretic peptide, *VTE* venous thromboembolism

**Fig. 3:**
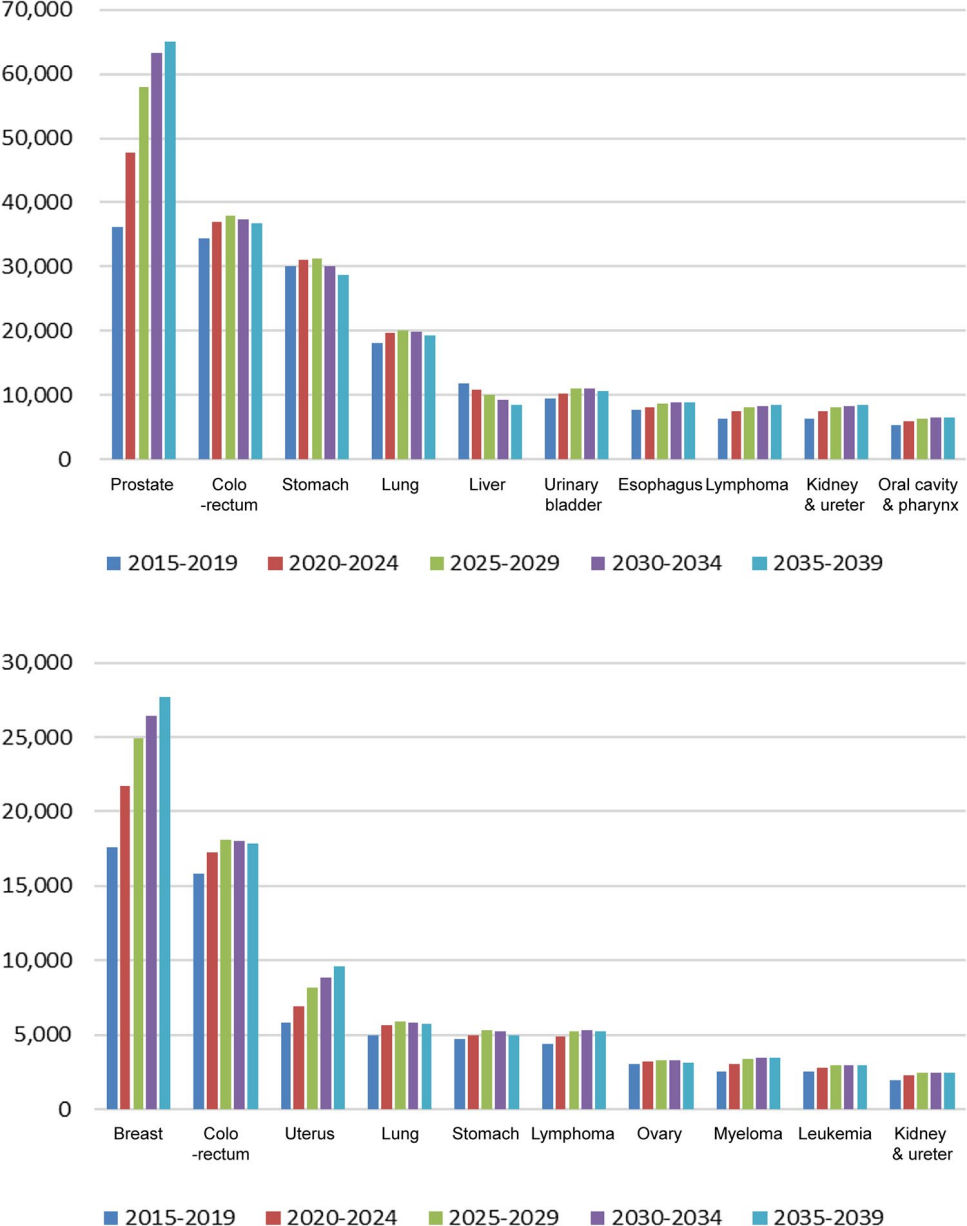
Sex-, and cancer type-specific projected number of CVDs for the top 10 cancers, 2015–2039. Bar graphs show the number of cancer patients with CVD stratified by 5-year intervals. The top 10 is not a ranking in all cancers but of cancers with CVD in 2015. CVD, cardiovascular disease

The top 5 male cancers in 2015 were prostate, stomach, colorectal, lung, and kidney/ureter (Supplementary Table 1, Fig. 2 ‘Cancer’ panel). The number of prostate cancer patients will increase 1.6-fold overall (Table 2) and 1.4-fold as a proportion of male cancers by 2035 (Fig. 2); the rank will change to 2nd or 3rd as colorectal cancer rates will increase while stomach cancer rates will decline by 2035. In general, a high CVD prevalence in elderly patients with various cancer types (Supplementary Table 2) increased the prevalence rank of each cancer (e.g., colorectal cancer in LVD, IHD, VTE, high NT-proBNP, or CVD, and urinary bladder cancer in LVD), while an average CVD prevalence in frequent cancers retained the latter’s higher proportions (e.g., prostate cancer with SD, AF, or CVD) in 2015. As cancer patients age, those with prostate cancer plus CVD will increase 1.5-fold in proportion by 2035 (Fig. 2) and 1.8-fold in number (Table 2; Fig. 3).

**Table 2:** Growth rate of cancer, cancer without CVD and cancer with CVD between 2015–2010 and 2035–2039 in 5 cancers

	2015–2019	2035–2039	Growth rate	Odds ratio (95% CI)	
All					
Cancer	3,081,680	3,652,280	1.19		
Cancer without CVD	2,828,369	3,339,698	1.18		
Cancer with CVD	253,311	312,582	1.23**	1.05	(1.04–1.05)
Male					
Cancer	1,722,590	2,060,650	1.20		
Cancer without CVD	1,539,893	1,839,169	1.19		
Cancer with CVD	182,697	221,481	1.21**	1.02	(1.01–1.02)
Prostatic cancer	450,160	727,500	1.62		
Prostatic cancer without CVD	414,059	662,479	1.60		
Prostatic cancer with CVD	36,101	65,021	1.80**	1.13	(1.11–1.14)
Colon and rectal cancer	297,370	308,650	1.04		
Colon and rectal cancer without CVD	263,017	271,845	1.03		
Colon and rectal cancer with CVD	34,353	36,805	1.07**	1.04	(1.02–1.05)
Stomach cancer	306,210	277,560	0.91		
Stomach cancer without CVD	276,176	248,902	0.90		
Stomach cancer with CVD	30,034	28,658	0.95**	1.06	(1.04–1.08)
Lung cancer	160,160	169,360	1.06		
Lung cancer without CVD	142,091	150,101	1.06		
Lung cancer with CVD	18,069	19,259	1.07	1.01	(0.99–1.03)
Kidney and ureter cancer	68,540	89,540	1.31		
Kidney and ureter cancer without CVD	62,237	81,028	1.30		
Kidney and ureter cancer with CVD	6303	8512	1.35*	1.04	(1.00–1.07)
Female					
Cancer	1,359,090	1,591,630	1.17		
Cancer without CVD	1,288,476	1,500,529	1.16		
Cancer with CVD	70,614	91,101	1.29**	1.11	(1.10–1.12)
Breast cancer	413,970	506,070	1.22		
Breast cancer without CVD	396,338	478,357	1.21		
Breast cancer with CVD	17,632	27,713	1.57**	1.30	(1.28–1.33)
Colon and rectal cancer	214,900	229,250	1.07		
Colon and rectal cancer without CVD	199,063	211,404	1.06		
Colon and rectal cancer with CVD	15,837	17,846	1.13**	1.06	(1.04–1.08)
Uterine cancer	128,240	185,990	1.45		
Uterine cancer without CVD	122,454	176,391	1.44		
Uterine cancer with CVD	5786	9599	1.66**	1.15	(1.11–1.19)
Lung cancer	110,760	127,010	1.15		
Lung cancer without CVD	105,780	121,316	1.15		
Lung cancer with CVD	4980	5694	1.14	1.00	(0.96–1.04)
Stomach cancer	136,310	129,750	0.95		
Stomach cancer without CVD	131,570	124,745	0.95		
Stomach cancer with CVD	4740	5005	1.06**	1.11	(1.07–1.16)

Asterisks indicate statistical differences in CVD prevalence between 2015–2019 and 2035–2039 for each cancer. (*P < 0.05, **P < 0.001). The odds ratio indicates the relative risk of CVD comorbidities in 2035–2039 in comparison with 2015–2019 for each cancer

*CI* confidence interval

### Proportion of cancer type in female CVD patients in 2015 and 2039

The top 5 cancers in 2015 were breast, colorectal, stomach, uterus, and lung (Supplementary Table 1, Fig. 2 ‘Cancer’ panel). In contrast to men, the proportion of women with cancer is projected to remain relatively unchanged. In general, a high CVD prevalence in elderly patients with various cancer types increased their rank proportions (e.g., colorectal cancer with SD, IHD, or VTE, as well as uterine cancer with LVDD, LVD, VTE, or CVD). Moreover, an average CVD prevalence in frequent cancers maintained their high rank proportions (e.g., breast cancer with LVDD, LVD, AF, AS, high NT-proBNP, or CVD) in 2015. Patients with VTE and high NT-proBNP had notably higher rates of gynecologic cancers and hematological malignancies, respectively. As cancer patients age, breast cancer patients with CVD will increase 1.2-fold in proportion (Fig. 2) and 1.6-fold in number by 2035 (Table 2; Fig. 3).

### Growth rates of top 5 cancers with and without CVD

The growth rates of the top 5 cancers with and without CVD were compared between 2015–2019 and 2035–2039 (Table 2). The growth rate of cancer with CVD is predicted to be larger than that of cancer without CVD (1.23 vs 1.18, *P* < 0.001). The prevalence of 4 of the top 5 cancers (excluding stomach cancer) will increase regardless of CVD, although these increases will be greater in patients with CVD. The growth rates of urological cancers (prostate and kidney and ureter) with CVD will be significantly higher in men (1.80 vs 1.60, *P* < 0.001 and 1.35 vs 1.30, *P* < 0.05, respectively), and those of breast and uterine cancers will be significantly higher in women (1.57 vs 1.21, *P* < 0.001 and 1.66 vs 1.44, *P* < 0.001, respectively), compared with the growth rates of patients with each corresponding cancer without CVD. The odds ratio of CVD comorbidity was high in male patients with prostatic cancer (1.13, 95% confidence interval [CI] 1.11–1.14) and in female patients with breast cancer (1.30, 95% CI 1.28–1.33), uterine cancer (1.15, 95% CI 1.11–1.19), and stomach cancer (1.11, 95% CI 1.07–1.16). Fewer female than male patients will have comorbid cancer/CVD; however, the odds ratio of CVD comorbidity will be larger in female than in male patients by 2035 (1.11, 95% CI 1.10–1.12 vs 1.02, 95% CI 1.01–1.02). The projected numbers of sex- and cancer type-specific CVDs in the top 10 cancers between 2015 and 2039 are presented in Fig. 3.

## Discussion

Japan is projected to face an HF pandemic [4, 14, 15] and cancer epidemic [3] in the coming 2 decades owing to an aging population. Therefore, we predicted the future number of cancer patients with CVD. Our study revealed the following new observations: (1) the total number of Japanese cancer patients with CVD was 253,000 in 2015, and is expected to increase rapidly by 30,000 by 2020, peaking at 313,000 in 2030–2034; (2) the CVD population will be predominantly men (2.5-fold the number of women) and ≥ 75 years of age; (3) the growth rate in the number of cancer patients ≥ 75 years will be greater in women than in men; hence, the growth rate in cancer patients with CVD will also be greater in women; and (4) therefore, cancer patients in 2035 will be older and more likely to have CVD than those in 2015, especially women.

While cancer patients with CVD will continue to be predominantly male (2.5 times the number of female patients), female patients will experience greater increases in the rate of cancer with CVD. While rates of cancer without CVD will increase 1.16-fold between 2015 and 2035, cancer with CVD rates will increase 1.29-fold. The odds ratio, which is the relative ratio between increasing rates of cancer with vs without CVD, will be 1.11 (Table 2). Breast and uterine cancers show the highest odds ratios at 1.30 and 1.15, respectively. Therefore, healthcare practitioners caring for female (i.e., breast and gynecological) cancers should prepare to treat CVD, especially LVD and VTE.

The cancer/CVD epidemic will persist in a decremental phase in Japan after 2005 owing to the aging population. As cancer and CVD are more common in individuals aged over 75 years, cancer centers will be obliged to improve medical care for CVD as well. Cancer patients in 2035 will be older and more likely to have CVD than cancer patients in 2015, especially among women. Japan is projected to face an epidemic of cancer with CVD; as a history of CVD is a strong predictor of relapse or MACE, cancer healthcare practitioners should recognize CVD presence and history. However, the appropriate management of CVD may be deficient owing to unawareness of its existence, failure of patients to mention CVD history (e.g., because of cognitive impairment), communication lapses between hospitals, and shorter medical record storage periods. Electronic medical records containing information on both CVD and cancer should be available at cancer centers.

Cancer and CVD incident cases have increased in the US owing to the aging Caucasian population [16, 17]. Cancer patients with CVD are prevalent [5] owing to the longer survival rates of patients with both diseases [18]. The prevalence of HF in patients aged 66 years or older with breast cancer, colorectal cancer, lung cancer, and prostatic cancer were 6.9%, 11.6%, 12.4% and 5.7%, respectively [5]. Although precise comparisons with previously published data are not feasible because of the different methods of assessment and cohort types, our cancer patients may have a lower prevalence of CVD than the US cohort (Supplementary Table 2). However, a future epidemic of cancer with CVD in Japan will likely occur given that Japan has the most rapidly aging population among developed countries [2, 3].

A number of limitations must also be considered. First, this pilot study was retrospective and observational; a prospective study would be preferable for the precise assessment of CVD burden on cancer patients. Second, this was a single-center study; the incidence rates of some cancers differ in Japan (e.g., stomach cancer), and Niigata Cancer Center Hospital may not be a representative Japanese cancer center. However, no single representative hospital has been selected to study the prevalence of cancer with CVD in Japan to date; thus, our hospital is the closest to realizing this goal owing to its suitable population size, average number of cancer patients at each type, and proper diagnosis by cardiologists. Furthermore, the CVD/cancer deaths (ratios) in 2014 in Niigata city and Japan overall were 153/292 (0.524) and 157/294 (0.535), respectively [19]. The ratio in Niigata city was the 15th closest to the overall ratio in Japan, which encompassed 47 prefectures and 21 major cities. CVD and cancer death in Niigata were sufficiently close to the nationwide average that our study can be deemed representative of the country. Third, CVD diagnoses were influenced by access to electrocardiography, echocardiography, vascular echo, CT, and NT-proBNP measurement; the identification of CVD in all cancer patients was not feasible. Moreover, there is a possibility that the actual prevalence of CVD in cancer patients was underestimated because of selection bias arising from the fact that patients with latent CVD in our hospital are not identified simply because of their non-referral to those examinations. Fourth, both our own public data and those of the National Center were based on past-year surveys. With advancing therapies and improved prognoses, future extrapolations may be modified. However, because survival rates in the future promise to improve with advancing anticancer and anti-CVD therapies, the actual number of patients living with cancer and CVD cannot be lower than our projection; in other words, it is highly unlikely that our projection is overestimated.

In conclusion, as cancer patients in Japan progressively age, comorbid CVD is expected to increase in prevalence in the near future. Cancer care providers should prepare the medical system for CVD management. The rapid growth of CVD in women with cancer should be recognized, especially in those with breast cancer and gynecological cancers.

## Electronic supplementary material

Below is the link to the electronic supplementary material.

Supplementary material 1 (DOCX 28 KB)

Supplementary material 2 (DOCX 35 KB)

Supplementary material 3 (DOCX 159 KB)
