# Supplementary material for: Future projection of cancer patients with cardiovascular disease in Japan by the year 2039: a pilot study
Source: Int J Clin Oncol. 2019 Mar 22;24(8):983–94. doi: 10.1007/s10147-019-01426-w (PMC6597732; doi:10.1007/s10147-019-01426-w)
Supplement: Supplementary file 1 — Supplementary material 1 (DOCX 28 KB) [file 10147_2019_1426_MOESM1_ESM.docx]

| **Supplementary Table 1**  Age-, gender-, and cancer sites-specific projected number, 2015 through 2039. (male) | | | | | | | |
| --- | --- | --- | --- | --- | --- | --- | --- |
| Sites | Year | Age groups | | | | | |
|  |  | 0-14 | 15-44 | 45-54 | 55-64 | 65-74 | 75+ |
| All  C00-C96 | 2015-2019 | 3,740 | 52,910 | 97,440 | 272,030 | 640,810 | 667,130 |
|  | 2020-2024 | 3,520 | 51,780 | 113,490 | 263,680 | 652,620 | 780,340 |
|  | 2025-2029 | 3,280 | 51,760 | 112,220 | 284,590 | 539,150 | 919,400 |
|  | 2030-2034 | 3,100 | 50,440 | 97,340 | 310,220 | 507,470 | 913,590 |
|  | 2035-2039 | 2,960 | 50,880 | 90,210 | 295,440 | 542,680 | 861,960 |
| Oral cavity &  pharynx  C00-C14 | 2015-2019 | 30 | 2,490 | 4,780 | 9,730 | 16,320 | 11,220 |
|  | 2020-2024 | 30 | 2,260 | 6,000 | 9,730 | 16,990 | 13,930 |
|  | 2025-2029 | 20 | 2,040 | 5,740 | 11,310 | 14,300 | 17,470 |
|  | 2030-2034 | 20 | 1,860 | 4,660 | 12,850 | 13,630 | 17,320 |
|  | 2035-2039 | 20 | 1,900 | 3,770 | 11,780 | 15,560 | 16,490 |
| Esophagus  C15 | 2015-2019 | - | 430 | 2,550 | 10,030 | 20,860 | 14,490 |
|  | 2020-2024 | - | 350 | 3,010 | 9,620 | 20,480 | 17,370 |
|  | 2025-2029 | - | 280 | 3,050 | 10,720 | 17,280 | 21,150 |
|  | 2030-2034 | - | 40 | 2,590 | 12,500 | 17,150 | 21,200 |
|  | 2035-2039 | - | 40 | 1,890 | 12,290 | 19,510 | 20,190 |
| Stomach  C16 | 2015-2019 | 30 | 5,300 | 15,900 | 52,520 | 117,130 | 115,330 |
|  | 2020-2024 | 30 | 4,650 | 16,880 | 45,370 | 115,060 | 127,570 |
|  | 2025-2029 | 30 | 4,150 | 16,440 | 44,230 | 91,520 | 146,980 |
|  | 2030-2034 | 30 | 3,440 | 14,650 | 47,090 | 81,190 | 144,980 |
|  | 2035-2039 | 20 | 3,380 | 12,890 | 45,330 | 81,470 | 134,470 |
| Colon &  rectum  C18-C20 | 2015-2019 | 10 | 7,210 | 19,590 | 52,430 | 109,830 | 108,300 |
|  | 2020-2024 | 10 | 6,390 | 21,690 | 51,020 | 109,540 | 125,050 |
|  | 2025-2029 | 10 | 6,210 | 20,500 | 54,950 | 90,600 | 146,500 |
|  | 2030-2034 | 10 | 5,550 | 17,440 | 58,790 | 87,300 | 145,470 |
|  | 2035-2039 | 10 | 5,550 | 16,140 | 54,370 | 93,940 | 138,640 |
| Liver  C22 | 2015-2019 | 110 | 870 | 3,610 | 11,830 | 25,030 | 26,440 |
|  | 2020-2024 | 100 | 690 | 3,730 | 9,990 | 21,930 | 25,350 |
|  | 2025-2029 | 90 | 600 | 3,510 | 10,180 | 16,340 | 25,490 |
|  | 2030-2034 | 90 | 520 | 2,950 | 10,920 | 14,640 | 23,370 |
|  | 2035-2039 | 80 | 500 | 2,620 | 10,280 | 15,420 | 20,230 |
| Gallbladder &  bile duct  C23-C24 | 2015-2019 | - | 270 | 770 | 2,790 | 7,990 | 10,780 |
|  | 2020-2024 | - | 240 | 900 | 2,540 | 7,990 | 12,200 |
|  | 2025-2029 | - | 190 | 890 | 2,730 | 6,330 | 14,310 |
|  | 2030-2034 | - | 80 | 830 | 3,060 | 5,780 | 14,290 |
|  | 2035-2039 | - | 80 | 600 | 2,940 | 6,280 | 13,460 |
| Pancreas  C25 | 2015-2019 | 10 | 330 | 1,220 | 3,550 | 7,890 | 6,500 |
|  | 2020-2024 | 10 | 300 | 1,310 | 3,340 | 7,850 | 7,740 |
|  | 2025-2029 | 10 | 220 | 1,240 | 3,520 | 6,360 | 9,320 |
|  | 2030-2034 | 10 | 180 | 1,050 | 3,670 | 5,930 | 9,120 |
|  | 2035-2039 | 10 | 180 | 760 | 3,410 | 6,280 | 8,380 |
| Larynx  C32 | 2015-2019 | - | 180 | 960 | 3,800 | 7,930 | 6,250 |
|  | 2020-2024 | - | 220 | 1,140 | 3,390 | 7,580 | 7,200 |
|  | 2025-2029 | - | 70 | 1,320 | 3,680 | 6,060 | 8,360 |
|  | 2030-2034 | - | 110 | 1,380 | 4,020 | 5,420 | 8,040 |
|  | 2035-2039 | - | 110 | 550 | 4,550 | 5,990 | 7,210 |
| Lung  C33-C34 | 2015-2019 | - | 2,170 | 7,540 | 25,360 | 63,640 | 61,450 |
|  | 2020-2024 | - | 2,260 | 8,100 | 24,250 | 66,350 | 71,930 |
|  | 2025-2029 | - | 2,220 | 7,640 | 25,180 | 55,680 | 87,010 |
|  | 2030-2034 | - | 2,190 | 7,050 | 25,790 | 51,990 | 87,740 |
|  | 2035-2039 | - | 2,230 | 6,500 | 23,770 | 53,980 | 82,880 |
| Skin  C43-C44 | 2015-2019 | 30 | 2,550 | 3,160 | 5,390 | 12,170 | 24,210 |
|  | 2020-2024 | 30 | 2,560 | 4,630 | 6,700 | 14,280 | 31,130 |
|  | 2025-2029 | 30 | 2,810 | 5,010 | 8,490 | 13,310 | 38,160 |
|  | 2030-2034 | 20 | 2,850 | 3,840 | 10,310 | 14,190 | 40,270 |
|  | 2035-2039 | 20 | 3,030 | 3,640 | 10,080 | 16,640 | 41,730 |
| Prostate  C61 | 2015-2019 | 10 | 120 | 3,950 | 50,200 | 194,580 | 201,300 |
|  | 2020-2024 | 10 | 80 | 3,200 | 51,010 | 234,180 | 288,440 |
|  | 2025-2029 | - | 50 | 2,920 | 49,160 | 219,170 | 400,370 |
|  | 2030-2034 | - | 20 | 1,840 | 43,270 | 213,760 | 457,360 |
|  | 2035-2039 | - | 20 | 1,030 | 38,320 | 207,100 | 481,030 |
| Urinary bladder  C67 | 2015-2019 | 10 | 600 | 2,330 | 8,170 | 21,510 | 28,230 |
|  | 2020-2024 | 10 | 490 | 2,090 | 7,520 | 21,520 | 32,370 |
|  | 2025-2029 | 10 | 410 | 1,750 | 7,310 | 17,030 | 38,880 |
|  | 2030-2034 | 10 | 310 | 1,470 | 6,780 | 15,890 | 40,330 |
|  | 2035-2039 | 10 | 310 | 1,210 | 5,820 | 15,770 | 39,050 |
| Kidney &  ureter  C64-C66, C68 | 2015-2019 | 230 | 3,080 | 7,780 | 13,900 | 23,600 | 19,950 |
|  | 2020-2024 | 220 | 2,760 | 9,480 | 15,460 | 27,600 | 25,230 |
|  | 2025-2029 | 200 | 2,220 | 8,820 | 18,570 | 25,390 | 32,790 |
|  | 2030-2034 | 190 | 2,310 | 6,600 | 19,970 | 25,780 | 34,770 |
|  | 2035-2039 | 180 | 2,440 | 4,820 | 17,680 | 29,610 | 34,810 |
| Brain  C70-C72 | 2015-2019 | 530 | 1,660 | 640 | 780 | 1,010 | 830 |
|  | 2020-2024 | 500 | 1,590 | 650 | 680 | 880 | 820 |
|  | 2025-2029 | 470 | 1,560 | 570 | 670 | 660 | 880 |
|  | 2030-2034 | 440 | 1,450 | 570 | 690 | 580 | 800 |
|  | 2035-2039 | 420 | 1,400 | 570 | 600 | 590 | 670 |
| Thyroid gland  C73 | 2015-2019 | 20 | 3,980 | 3,340 | 4,070 | 5,240 | 3,280 |
|  | 2020-2024 | 20 | 4,440 | 4,310 | 4,710 | 6,010 | 4,410 |
|  | 2025-2029 | 10 | 4,880 | 4,430 | 5,890 | 5,490 | 5,920 |
|  | 2030-2034 | 10 | 5,380 | 3,870 | 6,750 | 5,770 | 6,030 |
|  | 2035-2039 | 10 | 5,500 | 3,980 | 6,550 | 6,880 | 6,050 |
| Lymphoma  C81-C85, C96 | 2015-2019 | 390 | 4,840 | 5,970 | 11,580 | 17,860 | 16,390 |
|  | 2020-2024 | 370 | 5,100 | 7,110 | 12,320 | 20,540 | 20,410 |
|  | 2025-2029 | 350 | 5,150 | 7,190 | 13,380 | 19,600 | 25,490 |
|  | 2030-2034 | 330 | 5,040 | 6,480 | 14,340 | 19,230 | 27,080 |
|  | 2035-2039 | 310 | 5,160 | 5,810 | 13,730 | 20,330 | 27,510 |
| Myeloma  C88-C90 | 2015-2019 | - | 410 | 690 | 1,800 | 3,730 | 3,960 |
|  | 2020-2024 | - | 730 | 840 | 1,790 | 3,960 | 4,730 |
|  | 2025-2029 | - | 1,370 | 790 | 1,920 | 3,300 | 5,570 |
|  | 2030-2034 | - | 1,550 | 1,230 | 2,020 | 3,010 | 5,540 |
|  | 2035-2039 | - | 1,620 | 2,200 | 1,800 | 3,100 | 5,040 |
| Leukemia  C91-C95 | 2015-2019 | 1,410 | 3,530 | 1,940 | 2,530 | 4,050 | 3,290 |
|  | 2020-2024 | 1,320 | 3,420 | 2,350 | 2,610 | 4,130 | 3,770 |
|  | 2025-2029 | 1,230 | 3,510 | 2,330 | 2,870 | 3,470 | 4,470 |
|  | 2030-2034 | 1,170 | 3,310 | 2,110 | 3,270 | 3,430 | 4,410 |
|  | 2035-2039 | 1,120 | 3,260 | 2,090 | 3,140 | 3,710 | 4,220 |

| Age-, gender-, and cancer sites-specific projected number, 2015 through 2039. (female) | | | | | | | |
| --- | --- | --- | --- | --- | --- | --- | --- |
| Sites | Year | Age groups | | | | | |
|  |  | 0-14 | 15-44 | 45-54 | 55-64 | 65-74 | 75+ |
| All  C00-C96 | 2015-2019 | 3,130 | 126,730 | 181,430 | 239,000 | 383,630 | 465,460 |
|  | 2020-2024 | 2,940 | 124,060 | 205,390 | 255,790 | 406,870 | 552,570 |
|  | 2025-2029 | 2,740 | 120,650 | 200,880 | 289,460 | 358,740 | 662,760 |
|  | 2030-2034 | 2,590 | 115,080 | 179,890 | 308,300 | 363,490 | 690,760 |
|  | 2035-2039 | 2,460 | 115,990 | 162,800 | 292,270 | 400,670 | 697,440 |
| Oral cavity &  pharynx  C00-C14 | 2015-2019 | 30 | 1,730 | 2,190 | 3,070 | 5,450 | 8,080 |
|  | 2020-2024 | 30 | 1,650 | 2,590 | 3,510 | 5,770 | 9,570 |
|  | 2025-2029 | 30 | 1,540 | 2,670 | 4,060 | 5,190 | 11,330 |
|  | 2030-2034 | 30 | 1,470 | 2,320 | 4,480 | 5,580 | 11,700 |
|  | 2035-2039 | 30 | 1,470 | 1,950 | 4,460 | 6,260 | 12,160 |
| Esophagus  C15 | 2015-2019 | - | 220 | 730 | 1,830 | 3,350 | 3,490 |
|  | 2020-2024 | - | 230 | 820 | 1,910 | 3,390 | 4,100 |
|  | 2025-2029 | - | 210 | 1,000 | 2,130 | 2,930 | 4,910 |
|  | 2030-2034 | - | 190 | 980 | 2,410 | 3,110 | 5,100 |
|  | 2035-2039 | - | 180 | 870 | 2,760 | 3,440 | 5,130 |
| Stomach  C16 | 2015-2019 | - | 4,510 | 7,960 | 18,200 | 42,180 | 63,460 |
|  | 2020-2024 | - | 4,150 | 7,960 | 16,120 | 41,640 | 69,190 |
|  | 2025-2029 | - | 4,080 | 7,390 | 15,720 | 32,960 | 78,360 |
|  | 2030-2034 | - | 4,070 | 6,580 | 15,870 | 29,690 | 78,690 |
|  | 2035-2039 | - | 3,970 | 6,420 | 14,750 | 29,460 | 75,150 |
| Colon &  rectum  C18-C20 | 2015-2019 | - | 5,950 | 15,250 | 31,100 | 64,340 | 98,260 |
|  | 2020-2024 | - | 5,420 | 16,530 | 30,680 | 64,530 | 112,990 |
|  | 2025-2029 | - | 5,060 | 15,290 | 33,920 | 53,510 | 128,720 |
|  | 2030-2034 | - | 4,800 | 12,950 | 35,130 | 51,370 | 129,510 |
|  | 2035-2039 | - | 4,830 | 11,450 | 31,790 | 55,870 | 125,310 |
| Liver  C22 | 2015-2019 | 120 | 470 | 660 | 3,040 | 10,910 | 18,230 |
|  | 2020-2024 | 110 | 360 | 670 | 2,590 | 9,610 | 17,250 |
|  | 2025-2029 | 100 | 340 | 540 | 2,630 | 7,270 | 16,780 |
|  | 2030-2034 | 100 | 310 | 400 | 2,880 | 6,670 | 15,340 |
|  | 2035-2039 | 90 | 280 | 420 | 2,490 | 7,100 | 13,710 |
| Gallbladder &  bile duct  C23-C24 | 2015-2019 | - | 120 | 460 | 1,630 | 4,800 | 10,100 |
|  | 2020-2024 | - | 80 | 530 | 1,390 | 4,610 | 10,610 |
|  | 2025-2029 | - | 70 | 450 | 1,360 | 3,640 | 11,260 |
|  | 2030-2034 | - | 90 | 290 | 1,500 | 3,140 | 10,890 |
|  | 2035-2039 | - | 90 | 250 | 1,280 | 3,110 | 10,250 |
| Pancreas  C25 | 2015-2019 | 80 | 360 | 940 | 2,630 | 5,990 | 7,570 |
|  | 2020-2024 | 80 | 320 | 1,130 | 2,620 | 6,360 | 9,050 |
|  | 2025-2029 | 70 | 300 | 1,050 | 2,980 | 5,440 | 10,630 |
|  | 2030-2034 | 70 | 250 | 820 | 3,240 | 5,120 | 10,730 |
|  | 2035-2039 | 60 | 260 | 670 | 2,890 | 5,640 | 10,200 |
| Larynx  C32 | 2015-2019 | - | 20 | 80 | 320 | 450 | 460 |
|  | 2020-2024 | - | 10 | 110 | 260 | 420 | 530 |
|  | 2025-2029 | - | - | 100 | 280 | 450 | 600 |
|  | 2030-2034 | - | - | 70 | 340 | 400 | 690 |
|  | 2035-2039 | - | - | 10 | 320 | 450 | 710 |
| Lung  C33-C34 | 2015-2019 | 10 | 2,320 | 5,830 | 17,530 | 40,210 | 44,860 |
|  | 2020-2024 | 10 | 2,290 | 6,860 | 17,380 | 43,040 | 55,450 |
|  | 2025-2029 | 10 | 2,380 | 7,020 | 18,080 | 35,980 | 68,400 |
|  | 2030-2034 | 10 | 2,700 | 5,590 | 19,320 | 33,510 | 69,060 |
|  | 2035-2039 | 10 | 2,790 | 5,270 | 18,540 | 34,080 | 66,320 |
| Skin  C43-C44 | 2015-2019 | 80 | 2,450 | 2,360 | 4,010 | 9,500 | 28,190 |
|  | 2020-2024 | 80 | 2,810 | 3,140 | 4,830 | 10,980 | 36,450 |
|  | 2025-2029 | 70 | 2,880 | 3,750 | 5,720 | 10,140 | 44,050 |
|  | 2030-2034 | 70 | 2,770 | 3,680 | 6,510 | 10,640 | 47,890 |
|  | 2035-2039 | 70 | 2,890 | 3,280 | 7,080 | 11,810 | 51,370 |
| Breast  C50 | 2015-2019 | - | 49,770 | 90,160 | 89,120 | 104,620 | 80,300 |
|  | 2020-2024 | - | 44,950 | 100,720 | 101,030 | 119,070 | 111,040 |
|  | 2025-2029 | - | 39,980 | 93,240 | 115,410 | 111,460 | 150,420 |
|  | 2030-2034 | - | 36,350 | 75,540 | 118,350 | 115,920 | 165,500 |
|  | 2035-2039 | - | 37,580 | 61,710 | 105,560 | 126,930 | 174,290 |
| Uterus  C53-C55 | 2015-2019 | - | 32,390 | 31,030 | 26,850 | 22,860 | 15,110 |
|  | 2020-2024 | - | 35,030 | 38,590 | 33,810 | 25,220 | 18,920 |
|  | 2025-2029 | - | 35,680 | 41,260 | 43,210 | 24,980 | 24,080 |
|  | 2030-2034 | - | 32,600 | 42,610 | 48,840 | 29,130 | 25,400 |
|  | 2035-2039 | - | 33,050 | 40,210 | 49,650 | 35,950 | 27,130 |
| Ovary  C56 | 2015-2019 | 170 | 5,560 | 8,050 | 7,780 | 8,020 | 4,860 |
|  | 2020-2024 | 160 | 5,400 | 8,290 | 8,180 | 8,060 | 5,770 |
|  | 2025-2029 | 150 | 5,330 | 7,720 | 8,950 | 6,890 | 7,140 |
|  | 2030-2034 | 140 | 5,120 | 7,020 | 8,890 | 7,060 | 7,160 |
|  | 2035-2039 | 130 | 5,040 | 6,610 | 8,100 | 7,610 | 6,910 |
| Urinary bladder  C67 | 2015-2019 | - | 240 | 660 | 1,840 | 4,840 | 10,590 |
|  | 2020-2024 | - | 190 | 720 | 1,800 | 5,250 | 11,900 |
|  | 2025-2029 | - | 120 | 790 | 1,950 | 4,340 | 13,770 |
|  | 2030-2034 | - | 100 | 620 | 2,090 | 4,200 | 14,650 |
|  | 2035-2039 | - | 100 | 390 | 2,160 | 4,520 | 15,280 |
| Kidney &  ureter  C64-C66, C68 | 2015-2019 | 90 | 1,030 | 2,230 | 4,900 | 9,150 | 12,370 |
|  | 2020-2024 | 90 | 880 | 2,650 | 4,990 | 10,480 | 14,800 |
|  | 2025-2029 | 80 | 580 | 2,460 | 5,550 | 9,000 | 18,040 |
|  | 2030-2034 | 80 | 580 | 1,750 | 5,870 | 8,420 | 18,800 |
|  | 2035-2039 | 70 | 610 | 1,030 | 5,200 | 9,000 | 18,850 |
| Brain  C70-C72 | 2015-2019 | 470 | 870 | 630 | 700 | 1,100 | 950 |
|  | 2020-2024 | 440 | 710 | 560 | 590 | 920 | 930 |
|  | 2025-2029 | 410 | 640 | 410 | 570 | 660 | 930 |
|  | 2030-2034 | 390 | 550 | 350 | 520 | 570 | 850 |
|  | 2035-2039 | 370 | 520 | 310 | 390 | 570 | 750 |
| Thyroid gland  C73 | 2015-2019 | 40 | 12,420 | 10,160 | 10,470 | 13,410 | 8,630 |
|  | 2020-2024 | 40 | 13,350 | 12,230 | 11,400 | 13,770 | 10,910 |
|  | 2025-2029 | 40 | 13,910 | 12,770 | 13,290 | 12,150 | 13,580 |
|  | 2030-2034 | 40 | 14,680 | 11,770 | 14,830 | 12,390 | 13,660 |
|  | 2035-2039 | 30 | 14,710 | 11,760 | 14,870 | 14,010 | 13,280 |
| Lymphoma  C81-C85, C96 | 2015-2019 | 240 | 3,710 | 3,950 | 7,840 | 12,680 | 14,340 |
|  | 2020-2024 | 220 | 3,660 | 4,560 | 7,710 | 13,500 | 16,910 |
|  | 2025-2029 | 210 | 3,370 | 4,900 | 7,640 | 12,110 | 20,000 |
|  | 2030-2034 | 200 | 3,150 | 4,360 | 8,210 | 11,270 | 20,540 |
|  | 2035-2039 | 190 | 3,160 | 3,540 | 8,380 | 11,020 | 20,390 |
| Myeloma  C88-C90 | 2015-2019 | - | 270 | 710 | 1,560 | 3,310 | 4,870 |
|  | 2020-2024 | - | 380 | 820 | 1,820 | 3,480 | 5,850 |
|  | 2025-2029 | - | 320 | 900 | 2,220 | 3,120 | 6,800 |
|  | 2030-2034 | - | 210 | 1,100 | 2,220 | 3,290 | 7,000 |
|  | 2035-2039 | - | 220 | 880 | 2,340 | 3,770 | 6,810 |
| Leukemia  C91-C95 | 2015-2019 | 1,090 | 2,720 | 1,470 | 1,830 | 2,790 | 3,100 |
|  | 2020-2024 | 1,020 | 2,770 | 1,810 | 1,840 | 2,850 | 3,560 |
|  | 2025-2029 | 950 | 2,930 | 1,760 | 2,060 | 2,420 | 4,130 |
|  | 2030-2034 | 900 | 2,940 | 1,560 | 2,370 | 2,310 | 4,120 |
|  | 2035-2039 | 860 | 2,900 | 1,660 | 2,200 | 2,530 | 4,040 |
