# Supplementary material for: Future projection of cancer patients with cardiovascular disease in Japan by the year 2039: a pilot study
Source: Int J Clin Oncol. 2019 Mar 22;24(8):983–94. doi: 10.1007/s10147-019-01426-w (PMC6597732; doi:10.1007/s10147-019-01426-w)
Supplement: Supplementary file 2 — Supplementary material 2 (DOCX 35 KB) [file 10147_2019_1426_MOESM2_ESM.docx]

| **Supplementary Table 2** |  |  |  |  |  |  |
| --- | --- | --- | --- | --- | --- | --- |
| Prevalence of left ventricular systolic dysfunction in male | | | | | | |
| Age groups | 0-14 | 15-44 | 45-54 | 55-64 | 65-74 | 75- |
| Oral cavity & pharynx (C0-14) | 0.00 | 0.00 | 0.00 | 4.00 | 0.00 | 0.00 |
| Esophagus (C15) | 0.00 | 0.00 | 0.00 | 0.84 | 0.62 | 4.55 |
| Stomach (C16) | 0.00 | 0.00 | 0.00 | 0.00 | 1.44 | 1.97 |
| Colon & rectum (C18-20) | 0.00 | 0.00 | 0.78 | 0.60 | 0.88 | 3.88 |
| Liver (C22) | 0.00 | 0.00 | 0.00 | 0.00 | 0.00 | 5.26 |
| Gallbladder & bile duct (C23-24) | 0.00 | 0.00 | 0.00 | 0.00 | 0.00 | 0.00 |
| Pancreas (C25) | 0.00 | 0.00 | 0.00 | 0.00 | 0.00 | 0.00 |
| Larynx (C32) | 0.00 | 0.00 | 0.00 | 0.00 | 2.94 | 0.00 |
| Lung (C33-34) | 0.00 | 0.00 | 2.70 | 0.88 | 2.76 | 1.21 |
| Skin (C43-44) | 0.00 | 0.00 | 0.00 | 0.00 | 0.00 | 2.20 |
| Prostate (C61) | 0.00 | 0.00 | 0.00 | 0.47 | 1.25 | 1.62 |
| Urinary bladder (C67) | 0.00 | 0.00 | 2.44 | 0.57 | 1.96 | 5.11 |
| Kidney & ureter (C64-66, 68) | 0.00 | 0.00 | 0.00 | 1.79 | 1.46 | 1.89 |
| Thyroid gland (C73) | 0.00 | 0.00 | 0.00 | 2.78 | 0.00 | 0.00 |
| Lymphoma (C81-85, 96) | 0.00 | 0.00 | 2.86 | 0.00 | 3.17 | 0.00 |
| Myeloma (C88-90) | 0.00 | 0.00 | 0.00 | 20.00 | 5.00 | 0.00 |
| Leukemia (C91-95) | 5.56 | 0.00 | 7.69 | 5.56 | 16.67 | 0.00 |
| Other & unspecified primary sites | 0.00 | 1.56 | 0.00 | 1.41 | 1.56 | 0.00 |
|  |  |  |  |  |  |  |
| Prevalence of left ventricular systolic dysfunction in female | | | | | | |
| Age groups | 0-14 | 15-44 | 45-54 | 55-64 | 65-74 | 75- |
| Oral cavity & pharynx (C0-14) | 0.00 | 0.00 | 0.00 | 0.00 | 0.00 | 0.00 |
| Esophagus (C15) | 0.00 | 0.00 | 0.00 | 0.00 | 0.00 | 5.00 |
| Stomach (C16) | 0.00 | 0.00 | 0.00 | 0.00 | 0.35 | 0.00 |
| Colon & rectum (C18-20) | 0.00 | 0.00 | 0.00 | 0.00 | 0.43 | 1.95 |
| Liver (C22) | 0.00 | 0.00 | 0.00 | 0.00 | 0.00 | 0.00 |
| Gallbladder & bile duct (C23-24) | 0.00 | 0.00 | 0.00 | 0.00 | 4.35 | 0.00 |
| Pancreas (C25) | 0.00 | 0.00 | 0.00 | 0.00 | 0.00 | 0.00 |
| Larynx (C32) | 0.00 | 0.00 | 0.00 | 0.00 | 0.00 | 0.00 |
| Lung (C33-34) | 0.00 | 0.00 | 0.00 | 0.50 | 0.00 | 0.54 |
| Skin (C43-44) | 0.00 | 0.00 | 0.00 | 0.00 | 0.00 | 0.00 |
| Breast (C50) | 0.00 | 0.39 | 0.38 | 0.51 | 0.22 | 0.00 |
| Uterus (C53-55) | 0.00 | 0.00 | 0.00 | 0.00 | 0.83 | 0.00 |
| Ovary (C56) | 0.00 | 0.00 | 0.00 | 1.30 | 2.04 | 4.35 |
| Urinary bladder (C67) | 0.00 | 0.00 | 0.00 | 0.00 | 0.00 | 2.08 |
| Kidney & ureter (C64-66, 68) | 0.00 | 0.00 | 0.00 | 0.00 | 1.33 | 0.00 |
| Thyroid gland (C73) | 0.00 | 0.00 | 0.00 | 0.00 | 0.00 | 0.00 |
| Lymphoma (C81-85, 96) | 0.00 | 0.00 | 0.00 | 0.00 | 1.82 | 0.00 |
| Myeloma (C88-90) | 0.00 | 0.00 | 0.00 | 0.00 | 0.00 | 0.00 |
| Leukemia (C91-95) | 6.67 | 0.00 | 0.00 | 0.00 | 0.00 | 33.33 |
| Other & unspecified primary sites | 6.67 | 0.00 | 0.00 | 0.00 | 0.00 | 1.32 |
|  |  |  |  |  |  |  |
| Prevalence of left ventricular diastolic dysfunction in male | | | | | | |
| Age groups | 0-14 | 15-44 | 45-54 | 55-64 | 65-74 | 75- |
| Oral cavity & pharynx (C0-14) | 0.00 | 0.00 | 0.00 | 2.00 | 10.00 | 0.00 |
| Esophagus (C15) | 0.00 | 0.00 | 0.00 | 0.84 | 2.47 | 0.00 |
| Stomach (C16) | 0.00 | 0.00 | 0.78 | 1.32 | 1.60 | 3.09 |
| Colon & rectum (C18-20) | 0.00 | 1.96 | 1.56 | 1.51 | 3.24 | 2.43 |
| Liver (C22) | 0.00 | 0.00 | 0.00 | 0.00 | 0.00 | 5.26 |
| Gallbladder & bile duct (C23-24) | 0.00 | 0.00 | 0.00 | 0.00 | 5.56 | 3.85 |
| Pancreas (C25) | 0.00 | 0.00 | 0.00 | 5.56 | 5.56 | 7.69 |
| Larynx (C32) | 0.00 | 0.00 | 0.00 | 4.17 | 1.47 | 0.00 |
| Lung (C33-34) | 0.00 | 0.00 | 0.00 | 1.47 | 1.38 | 1.21 |
| Skin (C43-44) | 0.00 | 0.00 | 0.00 | 0.00 | 1.16 | 1.10 |
| Prostate (C61) | 0.00 | 0.00 | 0.00 | 0.23 | 0.75 | 1.42 |
| Urinary bladder (C67) | 0.00 | 0.00 | 0.00 | 2.87 | 2.45 | 7.95 |
| Kidney & ureter (C64-66, 68) | 0.00 | 0.00 | 0.00 | 3.57 | 3.65 | 1.89 |
| Thyroid gland (C73) | 0.00 | 0.00 | 0.00 | 2.78 | 0.00 | 0.00 |
| Lymphoma (C81-85, 96) | 0.00 | 0.00 | 0.00 | 3.90 | 1.59 | 2.38 |
| Myeloma (C88-90) | 0.00 | 0.00 | 0.00 | 6.67 | 10.00 | 0.00 |
| Leukemia (C91-95) | 0.00 | 0.00 | 7.69 | 0.00 | 0.00 | 0.00 |
| Other & unspecified primary sites | 0.00 | 0.00 | 0.00 | 1.41 | 1.56 | 0.00 |
|  |  |  |  |  |  |  |
| Prevalence of left ventricular diastolic dysfunction in female | | | | | | |
| Age groups | 0-14 | 15-44 | 45-54 | 55-64 | 65-74 | 75- |
| Oral cavity & pharynx (C0-14) | 0.00 | 0.00 | 0.00 | 0.00 | 0.00 | 0.00 |
| Esophagus (C15) | 0.00 | 0.00 | 0.00 | 0.00 | 0.00 | 5.00 |
| Stomach (C16) | 0.00 | 0.00 | 0.00 | 0.00 | 1.40 | 1.23 |
| Colon & rectum (C18-20) | 0.00 | 0.00 | 0.00 | 0.52 | 1.29 | 1.95 |
| Liver (C22) | 0.00 | 0.00 | 0.00 | 0.00 | 16.67 | 0.00 |
| Gallbladder & bile duct (C23-24) | 0.00 | 0.00 | 0.00 | 0.00 | 0.00 | 8.33 |
| Pancreas (C25) | 0.00 | 0.00 | 0.00 | 7.14 | 0.00 | 0.00 |
| Larynx (C32) | 0.00 | 0.00 | 0.00 | 0.00 | 0.00 | 0.00 |
| Lung (C33-34) | 0.00 | 0.00 | 0.00 | 0.50 | 1.00 | 0.00 |
| Skin (C43-44) | 0.00 | 0.00 | 0.00 | 0.00 | 1.35 | 0.00 |
| Breast (C50) | 0.00 | 0.20 | 0.25 | 0.64 | 2.19 | 2.56 |
| Uterus (C53-55) | 0.00 | 1.04 | 0.00 | 1.17 | 1.67 | 6.56 |
| Ovary (C56) | 0.00 | 0.00 | 1.18 | 0.00 | 2.04 | 0.00 |
| Urinary bladder (C67) | 0.00 | 0.00 | 0.00 | 0.00 | 0.00 | 2.08 |
| Kidney & ureter (C64-66, 68) | 0.00 | 0.00 | 0.00 | 5.00 | 0.00 | 5.41 |
| Thyroid gland (C73) | 0.00 | 0.00 | 0.00 | 0.00 | 0.00 | 0.00 |
| Lymphoma (C81-85, 96) | 0.00 | 0.00 | 0.00 | 0.00 | 3.64 | 2.13 |
| Myeloma (C88-90) | 0.00 | 0.00 | 0.00 | 11.11 | 6.67 | 20.00 |
| Leukemia (C91-95) | 0.00 | 0.00 | 0.00 | 0.00 | 7.69 | 16.67 |
| Other & unspecified primary sites | 0.00 | 0.00 | 0.00 | 0.00 | 0.00 | 2.63 |
|  |  |  |  |  |  |  |
| Prevalence of left ventricular dysfunction in male | | |  |  |  |  |
| Age groups | 0-14 | 15-44 | 45-54 | 55-64 | 65-74 | 75- |
| Oral cavity & pharynx (C0-14) | 0.00 | 0.00 | 0.00 | 6.00 | 10.00 | 0.00 |
| Esophagus (C15) | 0.00 | 0.00 | 0.00 | 1.68 | 3.09 | 4.55 |
| Stomach (C16) | 0.00 | 0.00 | 0.78 | 1.32 | 3.04 | 5.06 |
| Colon & rectum (C18-20) | 0.00 | 1.96 | 2.34 | 2.11 | 4.12 | 6.31 |
| Liver (C22) | 0.00 | 0.00 | 0.00 | 0.00 | 0.00 | 10.53 |
| Gallbladder & bile duct (C23-24) | 0.00 | 0.00 | 0.00 | 0.00 | 5.56 | 3.85 |
| Pancreas (C25) | 0.00 | 0.00 | 0.00 | 5.56 | 5.56 | 7.69 |
| Larynx (C32) | 0.00 | 0.00 | 0.00 | 4.17 | 4.41 | 0.00 |
| Lung (C33-34) | 0.00 | 0.00 | 2.70 | 2.35 | 4.15 | 2.42 |
| Skin (C43-44) | 0.00 | 0.00 | 0.00 | 0.00 | 1.16 | 3.30 |
| Prostate (C61) | 0.00 | 0.00 | 0.00 | 0.70 | 2.01 | 3.04 |
| Urinary bladder (C67) | 0.00 | 0.00 | 2.44 | 3.45 | 4.41 | 13.07 |
| Kidney & ureter (C64-66, 68) | 0.00 | 0.00 | 0.00 | 5.36 | 5.11 | 3.77 |
| Thyroid gland (C73) | 0.00 | 0.00 | 0.00 | 5.56 | 0.00 | 0.00 |
| Lymphoma (C81-85, 96) | 0.00 | 0.00 | 2.86 | 3.90 | 4.76 | 2.38 |
| Myeloma (C88-90) | 0.00 | 0.00 | 0.00 | 26.67 | 15.00 | 0.00 |
| Leukemia (C91-95) | 5.56 | 0.00 | 15.38 | 5.56 | 16.67 | 0.00 |
| Other & unspecified primary sites | 0.00 | 1.56 | 0.00 | 2.82 | 3.13 | 0.00 |
|  |  |  |  |  |  |  |
| Prevalence of left ventricular dysfunction in female | | | | | | |
| Age groups | 0-14 | 15-44 | 45-54 | 55-64 | 65-74 | 75- |
| Oral cavity & pharynx (C0-14) | 0.00 | 0.00 | 0.00 | 0.00 | 0.00 | 0.00 |
| Esophagus (C15) | 0.00 | 0.00 | 0.00 | 0.00 | 0.00 | 10.00 |
| Stomach (C16) | 0.00 | 0.00 | 0.00 | 0.00 | 1.75 | 1.23 |
| Colon & rectum (C18-20) | 0.00 | 0.00 | 0.00 | 0.52 | 1.72 | 3.90 |
| Liver (C22) | 0.00 | 0.00 | 0.00 | 0.00 | 16.67 | 0.00 |
| Gallbladder & bile duct (C23-24) | 0.00 | 0.00 | 0.00 | 0.00 | 4.35 | 8.33 |
| Pancreas (C25) | 0.00 | 0.00 | 0.00 | 7.14 | 0.00 | 0.00 |
| Larynx (C32) | 0.00 | 0.00 | 0.00 | 0.00 | 0.00 | 0.00 |
| Lung (C33-34) | 0.00 | 0.00 | 0.00 | 0.99 | 1.00 | 0.54 |
| Skin (C43-44) | 0.00 | 0.00 | 0.00 | 0.00 | 1.35 | 0.00 |
| Breast (C50) | 0.00 | 0.59 | 0.63 | 1.14 | 2.41 | 2.56 |
| Uterus (C53-55) | 0.00 | 1.04 | 0.00 | 1.17 | 2.50 | 6.56 |
| Ovary (C56) | 0.00 | 0.00 | 1.18 | 1.30 | 4.08 | 4.35 |
| Urinary bladder (C67) | 0.00 | 0.00 | 0.00 | 0.00 | 0.00 | 4.17 |
| Kidney & ureter (C64-66, 68) | 0.00 | 0.00 | 0.00 | 5.00 | 1.33 | 5.41 |
| Thyroid gland (C73) | 0.00 | 0.00 | 0.00 | 0.00 | 0.00 | 0.00 |
| Lymphoma (C81-85, 96) | 0.00 | 0.00 | 0.00 | 0.00 | 5.45 | 2.13 |
| Myeloma (C88-90) | 0.00 | 0.00 | 0.00 | 11.11 | 6.67 | 20.00 |
| Leukemia (C91-95) | 6.67 | 0.00 | 0.00 | 0.00 | 7.69 | 50.00 |
| Other & unspecified primary sites | 6.67 | 0.00 | 0.00 | 0.00 | 0.00 | 3.95 |
|  |  |  |  |  |  |  |
| Prevalence of atrial fibrillation in male | | | | | | |
| Age groups | 0-14 | 15-44 | 45-54 | 55-64 | 65-74 | 75- |
| Oral cavity & pharynx (C0-14) | 0.00 | 0.00 | 0.00 | 2.00 | 8.00 | 6.25 |
| Esophagus (C15) | 0.00 | 0.00 | 0.00 | 2.52 | 7.41 | 6.06 |
| Stomach (C16) | 0.00 | 0.00 | 0.00 | 2.07 | 4.17 | 7.30 |
| Colon & rectum (C18-20) | 0.00 | 0.00 | 1.56 | 2.11 | 5.29 | 7.77 |
| Liver (C22) | 0.00 | 0.00 | 0.00 | 5.88 | 12.50 | 21.05 |
| Gallbladder & bile duct (C23-24) | 0.00 | 0.00 | 0.00 | 5.00 | 0.00 | 11.54 |
| Pancreas (C25) | 0.00 | 16.67 | 0.00 | 11.11 | 5.56 | 23.08 |
| Larynx (C32) | 0.00 | 0.00 | 0.00 | 4.17 | 4.41 | 0.00 |
| Lung (C33-34) | 0.00 | 0.00 | 2.70 | 4.11 | 5.76 | 7.26 |
| Skin (C43-44) | 0.00 | 0.00 | 0.00 | 3.51 | 3.49 | 6.59 |
| Prostate (C61) | 0.00 | 0.00 | 0.00 | 1.40 | 3.76 | 5.07 |
| Urinary bladder (C67) | 0.00 | 0.00 | 2.44 | 2.30 | 6.86 | 9.66 |
| Kidney & ureter (C64-66, 68) | 0.00 | 0.00 | 3.28 | 4.46 | 5.84 | 1.89 |
| Thyroid gland (C73) | 0.00 | 0.00 | 0.00 | 2.78 | 9.09 | 0.00 |
| Lymphoma (C81-85, 96) | 10.00 | 0.00 | 5.71 | 3.90 | 4.76 | 2.38 |
| Myeloma (C88-90) | 0.00 | 0.00 | 0.00 | 6.67 | 10.00 | 14.29 |
| Leukemia (C91-95) | 0.00 | 0.00 | 0.00 | 11.11 | 0.00 | 0.00 |
| Other & unspecified primary sites | 0.00 | 0.00 | 0.00 | 1.41 | 1.56 | 3.92 |
|  |  |  |  |  |  |  |
| Prevalence of atrial fibrillation in female | | | | | |  |
| Age groups | 0-14 | 15-44 | 45-54 | 55-64 | 65-74 | 75- |
| Oral cavity & pharynx (C0-14) | 0.00 | 0.00 | 0.00 | 0.00 | 0.00 | 0.00 |
| Esophagus (C15) | 0.00 | 0.00 | 0.00 | 0.00 | 7.32 | 10.00 |
| Stomach (C16) | 0.00 | 0.00 | 0.00 | 0.00 | 0.35 | 2.47 |
| Colon & rectum (C18-20) | 0.00 | 0.00 | 0.00 | 0.52 | 2.58 | 2.60 |
| Liver (C22) | 0.00 | 0.00 | 0.00 | 0.00 | 0.00 | 0.00 |
| Gallbladder & bile duct (C23-24) | 0.00 | 0.00 | 0.00 | 0.00 | 4.35 | 8.33 |
| Pancreas (C25) | 0.00 | 0.00 | 0.00 | 0.00 | 3.85 | 0.00 |
| Larynx (C32) | 0.00 | 0.00 | 0.00 | 0.00 | 16.67 | 0.00 |
| Lung (C33-34) | 0.00 | 0.00 | 0.00 | 0.99 | 2.99 | 2.70 |
| Skin (C43-44) | 0.00 | 0.00 | 0.00 | 2.33 | 2.70 | 0.92 |
| Breast (C50) | 0.00 | 0.00 | 0.13 | 0.25 | 3.06 | 1.92 |
| Uterus (C53-55) | 0.00 | 0.00 | 0.00 | 0.39 | 2.50 | 8.20 |
| Ovary (C56) | 0.00 | 0.00 | 1.18 | 0.00 | 2.04 | 4.35 |
| Urinary bladder (C67) | 0.00 | 0.00 | 0.00 | 0.00 | 1.96 | 2.08 |
| Kidney & ureter (C64-66, 68) | 0.00 | 0.00 | 0.00 | 0.00 | 0.00 | 2.70 |
| Thyroid gland (C73) | 0.00 | 0.00 | 0.00 | 0.00 | 1.56 | 5.00 |
| Lymphoma (C81-85, 96) | 25.00 | 0.00 | 0.00 | 1.39 | 5.45 | 4.26 |
| Myeloma (C88-90) | 0.00 | 0.00 | 0.00 | 0.00 | 0.00 | 0.00 |
| Leukemia (C91-95) | 0.00 | 0.00 | 0.00 | 0.00 | 0.00 | 16.67 |
| Other & unspecified primary sites | 6.67 | 0.00 | 0.00 | 0.00 | 1.05 | 2.63 |
|  |  |  |  |  |  |  |
| Prevalence of ischemic heart disease in male | | | | | | |
| Age groups | 0-14 | 15-44 | 45-54 | 55-64 | 65-74 | 75- |
| Oral cavity & pharynx (C0-14) | 0.00 | 0.00 | 0.00 | 0.00 | 0.00 | 6.25 |
| Esophagus (C15) | 0.00 | 0.00 | 7.69 | 6.72 | 4.32 | 6.06 |
| Stomach (C16) | 0.00 | 0.00 | 1.55 | 2.45 | 3.69 | 4.49 |
| Colon & rectum (C18-20) | 0.00 | 3.92 | 1.56 | 1.51 | 4.12 | 5.83 |
| Liver (C22) | 0.00 | 0.00 | 0.00 | 0.00 | 4.17 | 10.53 |
| Gallbladder & bile duct (C23-24) | 0.00 | 0.00 | 0.00 | 0.00 | 5.56 | 0.00 |
| Pancreas (C25) | 0.00 | 0.00 | 0.00 | 11.11 | 5.56 | 0.00 |
| Larynx (C32) | 0.00 | 0.00 | 0.00 | 4.17 | 7.35 | 3.45 |
| Lung (C33-34) | 0.00 | 0.00 | 0.00 | 2.93 | 4.38 | 2.42 |
| Skin (C43-44) | 0.00 | 0.00 | 0.00 | 0.00 | 1.16 | 0.00 |
| Prostate (C61) | 0.00 | 0.00 | 0.00 | 0.93 | 1.51 | 2.43 |
| Urinary bladder (C67) | 0.00 | 0.00 | 2.44 | 1.72 | 3.92 | 4.55 |
| Kidney & ureter (C64-66, 68) | 0.00 | 0.00 | 0.00 | 3.57 | 4.38 | 1.89 |
| Thyroid gland (C73) | 0.00 | 0.00 | 2.94 | 2.78 | 0.00 | 0.00 |
| Lymphoma (C81-85, 96) | 0.00 | 0.00 | 2.86 | 2.60 | 1.59 | 7.14 |
| Myeloma (C88-90) | 0.00 | 0.00 | 0.00 | 13.33 | 0.00 | 0.00 |
| Leukemia (C91-95) | 0.00 | 0.00 | 7.69 | 5.56 | 0.00 | 0.00 |
| Other & unspecified primary sites | 0.00 | 0.78 | 1.79 | 0.00 | 3.13 | 0.00 |
|  |  |  |  |  |  |  |
| Prevalence of ischemic heart disease in female | | | | | | |
| Age groups | 0-14 | 15-44 | 45-54 | 55-64 | 65-74 | 75- |
| Oral cavity & pharynx (C0-14) | 0.00 | 0.00 | 0.00 | 0.00 | 0.00 | 0.00 |
| Esophagus (C15) | 0.00 | 0.00 | 0.00 | 0.00 | 2.44 | 5.00 |
| Stomach (C16) | 0.00 | 0.00 | 0.00 | 0.00 | 0.00 | 0.82 |
| Colon & rectum (C18-20) | 0.00 | 0.00 | 0.00 | 0.00 | 0.86 | 1.95 |
| Liver (C22) | 0.00 | 0.00 | 0.00 | 0.00 | 0.00 | 0.00 |
| Gallbladder & bile duct (C23-24) | 0.00 | 0.00 | 0.00 | 0.00 | 4.35 | 0.00 |
| Pancreas (C25) | 0.00 | 0.00 | 0.00 | 0.00 | 3.85 | 0.00 |
| Larynx (C32) | 0.00 | 0.00 | 0.00 | 33.33 | 0.00 | 0.00 |
| Lung (C33-34) | 0.00 | 0.00 | 0.00 | 0.00 | 0.66 | 0.54 |
| Skin (C43-44) | 0.00 | 0.00 | 0.00 | 0.00 | 0.00 | 0.00 |
| Breast (C50) | 0.00 | 0.20 | 0.13 | 0.25 | 0.66 | 0.64 |
| Uterus (C53-55) | 0.00 | 0.00 | 0.00 | 0.78 | 0.83 | 1.64 |
| Ovary (C56) | 0.00 | 0.00 | 0.00 | 5.19 | 2.04 | 4.35 |
| Urinary bladder (C67) | 0.00 | 0.00 | 0.00 | 0.00 | 0.00 | 0.00 |
| Kidney & ureter (C64-66, 68) | 0.00 | 0.00 | 0.00 | 0.00 | 0.00 | 0.00 |
| Thyroid gland (C73) | 0.00 | 0.00 | 0.00 | 0.00 | 0.00 | 0.00 |
| Lymphoma (C81-85, 96) | 0.00 | 0.00 | 0.00 | 1.39 | 0.00 | 2.13 |
| Myeloma (C88-90) | 0.00 | 0.00 | 0.00 | 0.00 | 0.00 | 0.00 |
| Leukemia (C91-95) | 0.00 | 0.00 | 0.00 | 0.00 | 0.00 | 0.00 |
| Other & unspecified primary sites | 0.00 | 0.00 | 0.00 | 0.00 | 0.00 | 1.32 |
|  |  |  |  |  |  |  |
| Prevalence of aortic stenosis in male | | | | | | |
| Age groups | 0-14 | 15-44 | 45-54 | 55-64 | 65-74 | 75- |
| Oral cavity & pharynx (C0-14) | 0.00 | 0.00 | 0.00 | 0.00 | 0.00 | 0.00 |
| Esophagus (C15) | 0.00 | 0.00 | 0.00 | 0.00 | 0.00 | 0.00 |
| Stomach (C16) | 0.00 | 0.00 | 0.00 | 0.38 | 0.32 | 0.84 |
| Colon & rectum (C18-20) | 0.00 | 0.00 | 0.00 | 0.00 | 0.00 | 1.94 |
| Liver (C22) | 0.00 | 0.00 | 0.00 | 0.00 | 4.17 | 0.00 |
| Gallbladder & bile duct (C23-24) | 0.00 | 0.00 | 0.00 | 0.00 | 5.56 | 3.85 |
| Pancreas (C25) | 0.00 | 0.00 | 0.00 | 0.00 | 0.00 | 0.00 |
| Larynx (C32) | 0.00 | 0.00 | 0.00 | 0.00 | 1.47 | 0.00 |
| Lung (C33-34) | 0.00 | 0.00 | 0.00 | 0.00 | 0.69 | 0.81 |
| Skin (C43-44) | 0.00 | 0.00 | 0.00 | 0.00 | 1.16 | 0.00 |
| Prostate (C61) | 0.00 | 0.00 | 0.00 | 0.00 | 0.25 | 0.81 |
| Urinary bladder (C67) | 0.00 | 0.00 | 0.00 | 0.00 | 1.47 | 3.41 |
| Kidney & ureter (C64-66, 68) | 0.00 | 0.00 | 0.00 | 0.00 | 0.00 | 1.89 |
| Thyroid gland (C73) | 0.00 | 0.00 | 0.00 | 0.00 | 0.00 | 0.00 |
| Lymphoma (C81-85, 96) | 0.00 | 0.00 | 0.00 | 0.00 | 0.00 | 0.00 |
| Myeloma (C88-90) | 0.00 | 0.00 | 0.00 | 0.00 | 0.00 | 0.00 |
| Leukemia (C91-95) | 0.00 | 0.00 | 0.00 | 0.00 | 16.67 | 14.29 |
| Other & unspecified primary sites | 0.00 | 0.00 | 0.00 | 0.00 | 0.00 | 1.96 |
|  |  |  |  |  |  |  |
| Prevalence of aortic stenosis in female | | | | | | |
| Age groups | 0-14 | 15-44 | 45-54 | 55-64 | 65-74 | 75- |
| Oral cavity & pharynx (C0-14) | 0.00 | 0.00 | 0.00 | 0.00 | 0.00 | 0.00 |
| Esophagus (C15) | 0.00 | 0.00 | 0.00 | 0.00 | 0.00 | 5.00 |
| Stomach (C16) | 0.00 | 0.00 | 0.00 | 0.00 | 0.70 | 0.82 |
| Colon & rectum (C18-20) | 0.00 | 0.00 | 0.00 | 0.00 | 0.00 | 0.00 |
| Liver (C22) | 0.00 | 0.00 | 0.00 | 0.00 | 0.00 | 10.00 |
| Gallbladder & bile duct (C23-24) | 0.00 | 0.00 | 0.00 | 0.00 | 0.00 | 0.00 |
| Pancreas (C25) | 0.00 | 0.00 | 0.00 | 0.00 | 0.00 | 0.00 |
| Larynx (C32) | 0.00 | 0.00 | 0.00 | 0.00 | 0.00 | 0.00 |
| Lung (C33-34) | 0.00 | 0.00 | 0.00 | 0.00 | 0.66 | 2.16 |
| Skin (C43-44) | 0.00 | 0.00 | 0.00 | 0.00 | 1.35 | 4.59 |
| Breast (C50) | 0.00 | 0.00 | 0.00 | 0.00 | 1.09 | 2.56 |
| Uterus (C53-55) | 0.00 | 0.00 | 0.00 | 0.00 | 0.83 | 0.00 |
| Ovary (C56) | 0.00 | 0.00 | 0.00 | 0.00 | 0.00 | 0.00 |
| Urinary bladder (C67) | 0.00 | 0.00 | 0.00 | 0.00 | 1.96 | 0.00 |
| Kidney & ureter (C64-66, 68) | 0.00 | 0.00 | 0.00 | 2.50 | 1.33 | 2.70 |
| Thyroid gland (C73) | 0.00 | 0.00 | 0.00 | 0.00 | 0.00 | 0.00 |
| Lymphoma (C81-85, 96) | 0.00 | 0.00 | 0.00 | 1.39 | 1.82 | 4.26 |
| Myeloma (C88-90) | 0.00 | 0.00 | 0.00 | 0.00 | 0.00 | 0.00 |
| Leukemia (C91-95) | 0.00 | 0.00 | 0.00 | 0.00 | 0.00 | 0.00 |
| Other & unspecified primary sites | 0.00 | 0.00 | 0.00 | 0.00 | 1.05 | 2.63 |
|  |  |  |  |  |  |  |
| Prevalence of venous thromboembolism in male | | | | | | |
| Age groups | 0-14 | 15-44 | 45-54 | 55-64 | 65-74 | 75- |
| Oral cavity & pharynx (C0-14) | 0.00 | 0.00 | 0.00 | 2.00 | 0.00 | 6.25 |
| Esophagus (C15) | 0.00 | 0.00 | 3.85 | 3.36 | 1.23 | 3.03 |
| Stomach (C16) | 0.00 | 0.00 | 0.00 | 0.75 | 0.48 | 1.12 |
| Colon & rectum (C18-20) | 0.00 | 0.00 | 3.13 | 0.30 | 0.88 | 2.91 |
| Liver (C22) | 0.00 | 0.00 | 0.00 | 0.00 | 0.00 | 0.00 |
| Gallbladder & bile duct (C23-24) | 0.00 | 0.00 | 0.00 | 0.00 | 0.00 | 3.85 |
| Pancreas (C25) | 0.00 | 0.00 | 25.00 | 0.00 | 5.56 | 0.00 |
| Larynx (C32) | 0.00 | 0.00 | 0.00 | 0.00 | 2.94 | 0.00 |
| Lung (C33-34) | 0.00 | 7.14 | 0.00 | 0.59 | 1.38 | 0.81 |
| Skin (C43-44) | 0.00 | 0.00 | 0.00 | 0.00 | 0.00 | 0.00 |
| Prostate (C61) | 0.00 | 0.00 | 0.00 | 0.00 | 0.63 | 1.01 |
| Urinary bladder (C67) | 0.00 | 0.00 | 2.44 | 0.00 | 0.98 | 0.57 |
| Kidney & ureter (C64-66, 68) | 0.00 | 0.00 | 0.00 | 0.00 | 3.65 | 0.00 |
| Thyroid gland (C73) | 0.00 | 0.00 | 0.00 | 5.56 | 0.00 | 0.00 |
| Lymphoma (C81-85, 96) | 10.00 | 3.57 | 0.00 | 1.30 | 1.59 | 2.38 |
| Myeloma (C88-90) | 0.00 | 0.00 | 0.00 | 0.00 | 0.00 | 0.00 |
| Leukemia (C91-95) | 0.00 | 0.00 | 0.00 | 0.00 | 0.00 | 0.00 |
| Other & unspecified primary sites | 0.00 | 2.34 | 3.57 | 1.41 | 3.13 | 3.92 |
|  |  |  |  |  |  |  |
| Prevalence of venous thromboembolism in female | | | | | | |
| Age groups | 0-14 | 15-44 | 45-54 | 55-64 | 65-74 | 75- |
| Oral cavity & pharynx (C0-14) | 0.00 | 0.00 | 0.00 | 0.00 | 0.00 | 0.00 |
| Esophagus (C15) | 0.00 | 0.00 | 0.00 | 0.00 | 4.88 | 0.00 |
| Stomach (C16) | 0.00 | 3.13 | 0.00 | 0.93 | 0.00 | 0.00 |
| Colon & rectum (C18-20) | 0.00 | 0.00 | 0.00 | 3.13 | 3.00 | 2.60 |
| Liver (C22) | 0.00 | 0.00 | 0.00 | 0.00 | 0.00 | 0.00 |
| Gallbladder & bile duct (C23-24) | 0.00 | 0.00 | 0.00 | 0.00 | 0.00 | 0.00 |
| Pancreas (C25) | 0.00 | 0.00 | 0.00 | 0.00 | 0.00 | 0.00 |
| Larynx (C32) | 0.00 | 0.00 | 0.00 | 0.00 | 0.00 | 0.00 |
| Lung (C33-34) | 0.00 | 0.00 | 0.00 | 0.50 | 0.66 | 0.54 |
| Skin (C43-44) | 0.00 | 0.00 | 0.00 | 0.00 | 0.00 | 0.00 |
| Breast (C50) | 0.00 | 0.20 | 0.13 | 0.51 | 0.44 | 0.64 |
| Uterus (C53-55) | 0.00 | 1.04 | 0.59 | 3.50 | 1.67 | 3.28 |
| Ovary (C56) | 0.00 | 1.79 | 3.53 | 5.19 | 2.04 | 4.35 |
| Urinary bladder (C67) | 0.00 | 0.00 | 0.00 | 0.00 | 0.00 | 2.08 |
| Kidney & ureter (C64-66, 68) | 0.00 | 0.00 | 5.26 | 0.00 | 4.00 | 2.70 |
| Thyroid gland (C73) | 0.00 | 0.00 | 0.00 | 0.00 | 0.00 | 0.00 |
| Lymphoma (C81-85, 96) | 25.00 | 5.56 | 0.00 | 4.17 | 3.64 | 0.00 |
| Myeloma (C88-90) | 0.00 | 0.00 | 0.00 | 0.00 | 6.67 | 0.00 |
| Leukemia (C91-95) | 0.00 | 0.00 | 0.00 | 0.00 | 7.69 | 33.33 |
| Other & unspecified primary sites | 0.00 | 0.21 | 0.00 | 1.15 | 3.16 | 1.32 |
|  |  |  |  |  |  |  |
| Prevalence of NT-proBNP > 900pg/mL in male |  |  |  |  |  |  |
| Age groups | 0-14 | 15-44 | 45-54 | 55-64 | 65-74 | 75- |
| Oral cavity & pharynx (C0-14) | 0.00 | 0.00 | 0.00 | 0.00 | 2.00 | 6.25 |
| Esophagus (C15) | 0.00 | 0.00 | 0.00 | 1.68 | 3.09 | 7.58 |
| Stomach (C16) | 0.00 | 0.00 | 0.00 | 0.56 | 0.80 | 3.93 |
| Colon & rectum (C18-20) | 0.00 | 0.00 | 0.78 | 0.30 | 1.18 | 5.83 |
| Liver (C22) | 0.00 | 0.00 | 0.00 | 0.00 | 0.00 | 15.79 |
| Gallbladder & bile duct (C23-24) | 0.00 | 0.00 | 0.00 | 0.00 | 0.00 | 7.69 |
| Pancreas (C25) | 0.00 | 0.00 | 0.00 | 5.56 | 5.56 | 0.00 |
| Larynx (C32) | 0.00 | 0.00 | 0.00 | 2.08 | 0.00 | 0.00 |
| Lung (C33-34) | 0.00 | 0.00 | 2.70 | 1.47 | 1.84 | 2.42 |
| Skin (C43-44) | 0.00 | 0.00 | 0.00 | 0.00 | 0.00 | 4.40 |
| Prostate (C61) | 0.00 | 0.00 | 0.00 | 0.23 | 0.75 | 1.01 |
| Urinary bladder (C67) | 0.00 | 0.00 | 0.00 | 0.00 | 0.98 | 2.84 |
| Kidney & ureter (C64-66, 68) | 0.00 | 0.00 | 0.00 | 1.79 | 2.19 | 0.00 |
| Thyroid gland (C73) | 0.00 | 0.00 | 0.00 | 0.00 | 0.00 | 0.00 |
| Lymphoma (C81-85, 96) | 0.00 | 0.00 | 2.86 | 1.30 | 0.00 | 4.76 |
| Myeloma (C88-90) | 0.00 | 0.00 | 0.00 | 13.33 | 5.00 | 14.29 |
| Leukemia (C91-95) | 18.52 | 5.26 | 0.00 | 11.11 | 16.67 | 14.29 |
| Other & unspecified primary sites | 0.00 | 0.00 | 0.00 | 0.00 | 3.13 | 0.00 |
|  |  |  |  |  |  |  |
| Prevalence of NT-proBNP > 900pg/mL in female |  |  |  |  |  |  |
| Age groups | 0-14 | 15-44 | 45-54 | 55-64 | 65-74 | 75- |
| Oral cavity & pharynx (C0-14) | 0.00 | 0.00 | 0.00 | 0.00 | 0.00 | 0.00 |
| Esophagus (C15) | 0.00 | 0.00 | 0.00 | 0.00 | 0.00 | 5.00 |
| Stomach (C16) | 0.00 | 0.00 | 0.00 | 0.00 | 0.35 | 0.82 |
| Colon & rectum (C18-20) | 0.00 | 0.00 | 0.00 | 0.52 | 0.86 | 2.60 |
| Liver (C22) | 0.00 | 0.00 | 0.00 | 0.00 | 0.00 | 0.00 |
| Gallbladder & bile duct (C23-24) | 0.00 | 0.00 | 0.00 | 0.00 | 0.00 | 0.00 |
| Pancreas (C25) | 0.00 | 0.00 | 0.00 | 0.00 | 3.85 | 0.00 |
| Larynx (C32) | 0.00 | 0.00 | 0.00 | 0.00 | 0.00 | 0.00 |
| Lung (C33-34) | 0.00 | 0.00 | 0.00 | 0.50 | 1.33 | 0.00 |
| Skin (C43-44) | 0.00 | 0.00 | 0.00 | 0.00 | 0.00 | 0.00 |
| Breast (C50) | 0.00 | 0.20 | 0.13 | 0.25 | 1.09 | 2.56 |
| Uterus (C53-55) | 0.00 | 0.00 | 0.00 | 0.39 | 2.50 | 3.28 |
| Ovary (C56) | 0.00 | 0.00 | 3.53 | 1.30 | 4.08 | 8.70 |
| Urinary bladder (C67) | 0.00 | 0.00 | 0.00 | 0.00 | 0.00 | 0.00 |
| Kidney & ureter (C64-66, 68) | 0.00 | 0.00 | 0.00 | 0.00 | 0.00 | 2.70 |
| Thyroid gland (C73) | 0.00 | 0.00 | 0.00 | 0.00 | 0.00 | 0.00 |
| Lymphoma (C81-85, 96) | 0.00 | 0.00 | 0.00 | 1.39 | 3.64 | 12.77 |
| Myeloma (C88-90) | 0.00 | 0.00 | 0.00 | 0.00 | 0.00 | 40.00 |
| Leukemia (C91-95) | 10.00 | 0.00 | 0.00 | 0.00 | 7.69 | 16.67 |
| Other & unspecified primary sites | 6.67 | 0.00 | 0.00 | 0.00 | 0.00 | 1.32 |
|  |  |  |  |  |  |  |
| Prevalence of cardiovascular disease in male | | | | |  |  |
| Age groups | 0-14 | 15-44 | 45-54 | 55-64 | 65-74 | 75- |
| Oral cavity & pharynx (C0-14) | 0.00 | 0.00 | 0.00 | 10.00 | 14.00 | 18.75 |
| Esophagus (C15) | 0.00 | 0.00 | 11.54 | 14.29 | 14.81 | 19.70 |
| Stomach (C16) | 0.00 | 0.00 | 2.33 | 6.40 | 9.46 | 13.20 |
| Colon & rectum (C18-20) | 0.00 | 3.92 | 8.59 | 6.04 | 11.76 | 15.05 |
| Liver (C22) | 0.00 | 0.00 | 0.00 | 5.88 | 16.67 | 26.32 |
| Gallbladder & bile duct (C23-24) | 0.00 | 0.00 | 0.00 | 5.00 | 5.56 | 19.23 |
| Pancreas (C25) | 0.00 | 16.67 | 25.00 | 16.67 | 16.67 | 23.08 |
| Larynx (C32) | 0.00 | 0.00 | 0.00 | 10.42 | 16.18 | 3.45 |
| Lung (C33-34) | 0.00 | 7.14 | 5.41 | 7.92 | 12.67 | 12.10 |
| Skin (C43-44) | 0.00 | 0.00 | 0.00 | 3.51 | 5.81 | 10.99 |
| Prostate (C61) | 0.00 | 0.00 | 0.00 | 3.04 | 7.28 | 10.14 |
| Urinary bladder (C67) | 0.00 | 0.00 | 9.76 | 7.47 | 12.25 | 21.02 |
| Kidney & ureter (C64-66, 68) | 0.00 | 0.00 | 3.28 | 11.61 | 12.41 | 7.55 |
| Thyroid gland (C73) | 0.00 | 0.00 | 2.94 | 8.33 | 9.09 | 0.00 |
| Lymphoma (C81-85, 96) | 0.00 | 3.57 | 8.57 | 9.09 | 12.70 | 14.29 |
| Myeloma (C88-90) | 0.00 | 0.00 | 0.00 | 33.33 | 25.00 | 14.29 |
| Leukemia (C91-95) | 20.37 | 5.26 | 15.38 | 16.67 | 16.67 | 14.29 |
| Other & unspecified primary sites | 0.00 | 3.91 | 3.57 | 5.63 | 6.25 | 5.88 |
|  |  |  |  |  |  |  |
| Prevalence of cardiovascular disease in female | | | | | | |
| Age groups | 0-14 | 15-44 | 45-54 | 55-64 | 65-74 | 75- |
| All (C0-96) | 19.23 | 0.87 | 0.89 | 2.97 | 6.00 | 7.92 |
| Oral cavity & pharynx (C0-14) | 0.00 | 0.00 | 0.00 | 0.00 | 0.00 | 0.00 |
| Esophagus (C15) | 0.00 | 0.00 | 0.00 | 0.00 | 14.63 | 15.00 |
| Stomach (C16) | 0.00 | 3.13 | 0.00 | 0.93 | 2.46 | 5.35 |
| Colon & rectum (C18-20) | 0.00 | 0.00 | 0.00 | 4.17 | 7.73 | 9.74 |
| Liver (C22) | 0.00 | 0.00 | 0.00 | 0.00 | 16.67 | 0.00 |
| Gallbladder & bile duct (C23-24) | 0.00 | 0.00 | 0.00 | 0.00 | 4.35 | 8.33 |
| Pancreas (C25) | 0.00 | 0.00 | 0.00 | 7.14 | 3.85 | 0.00 |
| Larynx (C32) | 0.00 | 0.00 | 0.00 | 33.33 | 16.67 | 0.00 |
| Lung (C33-34) | 0.00 | 0.00 | 1.59 | 2.48 | 5.65 | 4.86 |
| Skin (C43-44) | 0.00 | 0.00 | 0.00 | 2.33 | 4.05 | 1.83 |
| Breast (C50) | 0.00 | 0.98 | 0.63 | 2.03 | 7.22 | 8.97 |
| Uterus (C53-55) | 0.00 | 2.08 | 0.00 | 5.06 | 6.67 | 14.75 |
| Ovary (C56) | 0.00 | 1.79 | 8.24 | 10.39 | 10.20 | 13.04 |
| Urinary bladder (C67) | 0.00 | 0.00 | 0.00 | 0.00 | 3.92 | 8.33 |
| Kidney & ureter (C64-66, 68) | 0.00 | 0.00 | 5.26 | 5.00 | 6.67 | 8.11 |
| Thyroid gland (C73) | 0.00 | 0.00 | 0.00 | 0.00 | 1.56 | 5.00 |
| Lymphoma (C81-85, 96) | 0.00 | 2.78 | 0.00 | 8.33 | 9.09 | 17.02 |
| Myeloma (C88-90) | 0.00 | 0.00 | 0.00 | 11.11 | 13.33 | 40.00 |
| Leukemia (C91-95) | 23.33 | 11.11 | 0.00 | 0.00 | 15.38 | 50.00 |
| Other & unspecified primary sites | 20.00 | 0.00 | 0.00 | 1.15 | 2.11 | 10.53 |
