# Supplementary material for: Future projection of cancer patients with cardiovascular disease in Japan by the year 2039: a pilot study
Source: Int J Clin Oncol. 2019 Mar 22;24(8):983–94. doi: 10.1007/s10147-019-01426-w (PMC6597732; doi:10.1007/s10147-019-01426-w)
Supplement: Supplementary file 3 — Supplementary material 3 (DOCX 159 KB) [file 10147_2019_1426_MOESM3_ESM.docx]

| **Supplementary Table 3.** | | | | |  |  |  |  |  |
| --- | --- | --- | --- | --- | --- | --- | --- | --- | --- |
| Left ventricular systolic dysfunction in male | | | | |  |  |  |  |  |
| Sites | Year | Age groups | | | | | | | |
|  |  | 0-14 | | 15-44 | 45-54 | 55-64 | 65-74 | 75+ | Total |
| Oral cavity | 2015-2019 | 0 | | 0 | 0 | 389 | 0 | 0 | 389 |
| & pharynx | 2020-2024 | 0 | | 0 | 0 | 389 | 0 | 0 | 389 |
| C00-C14 | 2025-2029 | 0 | | 0 | 0 | 452 | 0 | 0 | 452 |
|  | 2030-2034 | 0 | | 0 | 0 | 514 | 0 | 0 | 514 |
|  | 2035-2039 | 0 | | 0 | 0 | 471 | 0 | 0 | 471 |
| Esophagus | 2015-2019 | 0 | | 0 | 0 | 84 | 129 | 659 | 872 |
| C15 | 2020-2024 | 0 | | 0 | 0 | 81 | 126 | 790 | 997 |
|  | 2025-2029 | 0 | | 0 | 0 | 90 | 107 | 961 | 1,158 |
|  | 2030-2034 | 0 | | 0 | 0 | 105 | 106 | 964 | 1,175 |
|  | 2035-2039 | 0 | | 0 | 0 | 103 | 120 | 918 | 1,141 |
| Stomach | 2015-2019 | 0 | | 0 | 0 | 0 | 1,689 | 2,268 | 3,957 |
| C16 | 2020-2024 | 0 | | 0 | 0 | 0 | 1,660 | 2,508 | 4,168 |
|  | 2025-2029 | 0 | | 0 | 0 | 0 | 1,320 | 2,890 | 4,210 |
|  | 2030-2034 | 0 | | 0 | 0 | 0 | 1,171 | 2,851 | 4,022 |
|  | 2035-2039 | 0 | | 0 | 0 | 0 | 1,175 | 2,644 | 3,819 |
| Colon | 2015-2019 | 0 | | 0 | 153 | 317 | 969 | 4,206 | 5,645 |
| & rectum | 2020-2024 | 0 | | 0 | 169 | 308 | 967 | 4,856 | 6,301 |
| C18-C20 | 2025-2029 | 0 | | 0 | 160 | 332 | 799 | 5,689 | 6,981 |
|  | 2030-2034 | 0 | | 0 | 136 | 355 | 770 | 5,649 | 6,911 |
|  | 2035-2039 | 0 | | 0 | 126 | 329 | 829 | 5,384 | 6,668 |
| Liver | 2015-2019 | 0 | | 0 | 0 | 0 | 0 | 1,392 | 1,392 |
| C22 | 2020-2024 | 0 | | 0 | 0 | 0 | 0 | 1,334 | 1,334 |
|  | 2025-2029 | 0 | | 0 | 0 | 0 | 0 | 1,342 | 1,342 |
|  | 2030-2034 | 0 | | 0 | 0 | 0 | 0 | 1,230 | 1,230 |
|  | 2035-2039 | 0 | | 0 | 0 | 0 | 0 | 1,065 | 1,065 |
| Gallbladder | 2015-2019 | 0 | | 0 | 0 | 0 | 0 | 0 | 0 |
| & bile duct | 2020-2024 | 0 | | 0 | 0 | 0 | 0 | 0 | 0 |
| C23-C24 | 2025-2029 | 0 | | 0 | 0 | 0 | 0 | 0 | 0 |
|  | 2030-2034 | 0 | | 0 | 0 | 0 | 0 | 0 | 0 |
|  | 2035-2039 | 0 | | 0 | 0 | 0 | 0 | 0 | 0 |
| Pancreas | 2015-2019 | 0 | | 0 | 0 | 0 | 0 | 0 | 0 |
| C25 | 2020-2024 | 0 | | 0 | 0 | 0 | 0 | 0 | 0 |
|  | 2025-2029 | 0 | | 0 | 0 | 0 | 0 | 0 | 0 |
|  | 2030-2034 | 0 | | 0 | 0 | 0 | 0 | 0 | 0 |
|  | 2035-2039 | 0 | | 0 | 0 | 0 | 0 | 0 | 0 |
| Larynx | 2015-2019 | 0 | | 0 | 0 | 0 | 233 | 0 | 233 |
| C32 | 2020-2024 | 0 | | 0 | 0 | 0 | 223 | 0 | 223 |
|  | 2025-2029 | 0 | | 0 | 0 | 0 | 178 | 0 | 178 |
|  | 2030-2034 | 0 | | 0 | 0 | 0 | 159 | 0 | 159 |
|  | 2035-2039 | 0 | | 0 | 0 | 0 | 176 | 0 | 176 |
| Lung | 2015-2019 | 0 | | 0 | 204 | 223 | 1,760 | 743 | 2,930 |
| C33-C34 | 2020-2024 | 0 | | 0 | 219 | 213 | 1,835 | 870 | 3,137 |
|  | 2025-2029 | 0 | | 0 | 206 | 222 | 1,540 | 1,053 | 3,020 |
|  | 2030-2034 | 0 | | 0 | 191 | 227 | 1,438 | 1,061 | 2,916 |
|  | 2035-2039 | 0 | | 0 | 176 | 209 | 1,493 | 1,003 | 2,880 |
| Skin | 2015-2019 | 0 | | 0 | 0 | 0 | 0 | 532 | 532 |
| C43-C44 | 2020-2024 | 0 | | 0 | 0 | 0 | 0 | 684 | 684 |
|  | 2025-2029 | 0 | | 0 | 0 | 0 | 0 | 839 | 839 |
|  | 2030-2034 | 0 | | 0 | 0 | 0 | 0 | 885 | 885 |
|  | 2035-2039 | 0 | | 0 | 0 | 0 | 0 | 917 | 917 |
| Prostate | 2015-2019 | 0 | | 0 | 0 | 235 | 2,441 | 3,267 | 5,943 |
| C61 | 2020-2024 | 0 | | 0 | 0 | 238 | 2,938 | 4,681 | 7,857 |
|  | 2025-2029 | 0 | | 0 | 0 | 230 | 2,750 | 6,497 | 9,477 |
|  | 2030-2034 | 0 | | 0 | 0 | 202 | 2,682 | 7,422 | 10,306 |
|  | 2035-2039 | 0 | | 0 | 0 | 179 | 2,598 | 7,806 | 10,583 |
| Urinary | 2015-2019 | 0 | | 0 | 57 | 47 | 422 | 1,444 | 1,969 |
| bladder | 2020-2024 | 0 | | 0 | 51 | 43 | 422 | 1,655 | 2,171 |
| C67 | 2025-2029 | 0 | | 0 | 43 | 42 | 334 | 1,988 | 2,407 |
|  | 2030-2034 | 0 | | 0 | 36 | 39 | 312 | 2,062 | 2,449 |
|  | 2035-2039 | 0 | | 0 | 30 | 33 | 309 | 1,997 | 2,369 |
| Kidney | 2015-2019 | 0 | | 0 | 0 | 248 | 345 | 376 | 969 |
| & ureter | 2020-2024 | 0 | | 0 | 0 | 276 | 403 | 476 | 1,155 |
| C64-C66, | 2025-2029 | 0 | | 0 | 0 | 332 | 371 | 619 | 1,321 |
| C68 | 2030-2034 | 0 | | 0 | 0 | 357 | 376 | 656 | 1,389 |
|  | 2035-2039 | 0 | | 0 | 0 | 316 | 432 | 657 | 1,405 |
| Thyroid | 2015-2019 | 0 | | 0 | 0 | 113 | 0 | 0 | 113 |
| gland | 2020-2024 | 0 | | 0 | 0 | 131 | 0 | 0 | 131 |
| C73 | 2025-2029 | 0 | | 0 | 0 | 164 | 0 | 0 | 164 |
|  | 2030-2034 | 0 | | 0 | 0 | 188 | 0 | 0 | 188 |
|  | 2035-2039 | 0 | | 0 | 0 | 182 | 0 | 0 | 182 |
| Lymphoma | 2015-2019 | 0 | | 0 | 171 | 0 | 567 | 0 | 738 |
| C81-C85, | 2020-2024 | 0 | | 0 | 203 | 0 | 652 | 0 | 855 |
| C96 | 2025-2029 | 0 | | 0 | 205 | 0 | 622 | 0 | 828 |
|  | 2030-2034 | 0 | | 0 | 185 | 0 | 610 | 0 | 796 |
|  | 2035-2039 | 0 | | 0 | 166 | 0 | 645 | 0 | 811 |
| Myeloma | 2015-2019 | 0 | | 0 | 0 | 360 | 187 | 0 | 547 |
| C88-C90 | 2020-2024 | 0 | | 0 | 0 | 358 | 198 | 0 | 556 |
|  | 2025-2029 | 0 | | 0 | 0 | 384 | 165 | 0 | 549 |
|  | 2030-2034 | 0 | | 0 | 0 | 404 | 151 | 0 | 555 |
|  | 2035-2039 | 0 | | 0 | 0 | 360 | 155 | 0 | 515 |
| Leukemia | 2015-2019 | 78 | | 0 | 149 | 141 | 675 | 0 | 1,043 |
| C91-C95 | 2020-2024 | 73 | | 0 | 181 | 145 | 688 | 0 | 1,087 |
|  | 2025-2029 | 68 | | 0 | 179 | 159 | 578 | 0 | 985 |
|  | 2030-2034 | 65 | | 0 | 162 | 182 | 572 | 0 | 981 |
|  | 2035-2039 | 62 | | 0 | 161 | 174 | 618 | 0 | 1,016 |
| Total | 2015-2019 | 78 | | 0 | 733 | 2,157 | 9,416 | 14,886 | 27,271 |
|  | 2020-2024 | 73 | | 0 | 823 | 2,183 | 10,112 | 17,855 | 31,046 |
|  | 2025-2029 | 68 | | 0 | 794 | 2,406 | 8,764 | 21,877 | 33,910 |
|  | 2030-2034 | 65 | | 0 | 710 | 2,572 | 8,347 | 22,780 | 34,474 |
|  | 2035-2039 | 62 | | 0 | 658 | 2,357 | 8,552 | 22,390 | 34,019 |
|  |  |  | |  |  |  |  |  |  |
| Left ventricular systolic dysfunction in female | | | | |  |  |  |  |  |
| Sites | Year | Age groups | | | | | | | |
|  |  | 0-14 | | 15-44 | 45-54 | 55-64 | 65-74 | 75+ | Total |
| Oral cavity | 2015-2019 | 0 | | 0 | 0 | 0 | 0 | 0 | 0 |
| & pharynx | 2020-2024 | 0 | | 0 | 0 | 0 | 0 | 0 | 0 |
| C00-C14 | 2025-2029 | 0 | | 0 | 0 | 0 | 0 | 0 | 0 |
|  | 2030-2034 | 0 | | 0 | 0 | 0 | 0 | 0 | 0 |
|  | 2035-2039 | 0 | | 0 | 0 | 0 | 0 | 0 | 0 |
| Esophagus | 2015-2019 | 0 | | 0 | 0 | 0 | 0 | 175 | 175 |
| C15 | 2020-2024 | 0 | | 0 | 0 | 0 | 0 | 205 | 205 |
|  | 2025-2029 | 0 | | 0 | 0 | 0 | 0 | 246 | 246 |
|  | 2030-2034 | 0 | | 0 | 0 | 0 | 0 | 255 | 255 |
|  | 2035-2039 | 0 | | 0 | 0 | 0 | 0 | 257 | 257 |
| Stomach | 2015-2019 | 0 | | 0 | 0 | 0 | 148 | 0 | 148 |
| C16 | 2020-2024 | 0 | | 0 | 0 | 0 | 146 | 0 | 146 |
|  | 2025-2029 | 0 | | 0 | 0 | 0 | 116 | 0 | 116 |
|  | 2030-2034 | 0 | | 0 | 0 | 0 | 104 | 0 | 104 |
|  | 2035-2039 | 0 | | 0 | 0 | 0 | 103 | 0 | 103 |
| Colon | 2015-2019 | 0 | | 0 | 0 | 0 | 276 | 1,914 | 2,190 |
| & rectum | 2020-2024 | 0 | | 0 | 0 | 0 | 277 | 2,201 | 2,478 |
| C18-C20 | 2025-2029 | 0 | | 0 | 0 | 0 | 230 | 2,508 | 2,737 |
|  | 2030-2034 | 0 | | 0 | 0 | 0 | 220 | 2,523 | 2,743 |
|  | 2035-2039 | 0 | | 0 | 0 | 0 | 240 | 2,441 | 2,681 |
| Liver | 2015-2019 | 0 | | 0 | 0 | 0 | 0 | 0 | 0 |
| C22 | 2020-2024 | 0 | | 0 | 0 | 0 | 0 | 0 | 0 |
|  | 2025-2029 | 0 | | 0 | 0 | 0 | 0 | 0 | 0 |
|  | 2030-2034 | 0 | | 0 | 0 | 0 | 0 | 0 | 0 |
|  | 2035-2039 | 0 | | 0 | 0 | 0 | 0 | 0 | 0 |
| Gallbladder | 2015-2019 | 0 | | 0 | 0 | 0 | 209 | 0 | 209 |
| & bile duct | 2020-2024 | 0 | | 0 | 0 | 0 | 200 | 0 | 200 |
| C23-C24 | 2025-2029 | 0 | | 0 | 0 | 0 | 158 | 0 | 158 |
|  | 2030-2034 | 0 | | 0 | 0 | 0 | 137 | 0 | 137 |
|  | 2035-2039 | 0 | | 0 | 0 | 0 | 135 | 0 | 135 |
| Pancreas | 2015-2019 | 0 | | 0 | 0 | 0 | 0 | 0 | 0 |
| C25 | 2020-2024 | 0 | | 0 | 0 | 0 | 0 | 0 | 0 |
|  | 2025-2029 | 0 | | 0 | 0 | 0 | 0 | 0 | 0 |
|  | 2030-2034 | 0 | | 0 | 0 | 0 | 0 | 0 | 0 |
|  | 2035-2039 | 0 | | 0 | 0 | 0 | 0 | 0 | 0 |
| Larynx | 2015-2019 | 0 | | 0 | 0 | 0 | 0 | 0 | 0 |
| C32 | 2020-2024 | 0 | | 0 | 0 | 0 | 0 | 0 | 0 |
|  | 2025-2029 | 0 | | 0 | 0 | 0 | 0 | 0 | 0 |
|  | 2030-2034 | 0 | | 0 | 0 | 0 | 0 | 0 | 0 |
|  | 2035-2039 | 0 | | 0 | 0 | 0 | 0 | 0 | 0 |
| Lung | 2015-2019 | 0 | | 0 | 0 | 87 | 0 | 242 | 329 |
| C33-C34 | 2020-2024 | 0 | | 0 | 0 | 86 | 0 | 300 | 386 |
|  | 2025-2029 | 0 | | 0 | 0 | 90 | 0 | 370 | 459 |
|  | 2030-2034 | 0 | | 0 | 0 | 96 | 0 | 373 | 469 |
|  | 2035-2039 | 0 | | 0 | 0 | 92 | 0 | 358 | 450 |
| Skin | 2015-2019 | 0 | | 0 | 0 | 0 | 0 | 0 | 0 |
| C43-C44 | 2020-2024 | 0 | | 0 | 0 | 0 | 0 | 0 | 0 |
|  | 2025-2029 | 0 | | 0 | 0 | 0 | 0 | 0 | 0 |
|  | 2030-2034 | 0 | | 0 | 0 | 0 | 0 | 0 | 0 |
|  | 2035-2039 | 0 | | 0 | 0 | 0 | 0 | 0 | 0 |
| Breast | 2015-2019 | 0 | | 196 | 342 | 453 | 229 | 0 | 1,220 |
| C50 | 2020-2024 | 0 | | 177 | 382 | 513 | 261 | 0 | 1,333 |
|  | 2025-2029 | 0 | | 157 | 354 | 587 | 244 | 0 | 1,342 |
|  | 2030-2034 | 0 | | 143 | 287 | 602 | 254 | 0 | 1,285 |
|  | 2035-2039 | 0 | | 148 | 234 | 537 | 278 | 0 | 1,196 |
| Uterus | 2015-2019 | 0 | | 0 | 0 | 0 | 191 | 0 | 191 |
| C53-C55 | 2020-2024 | 0 | | 0 | 0 | 0 | 210 | 0 | 210 |
|  | 2025-2029 | 0 | | 0 | 0 | 0 | 208 | 0 | 208 |
|  | 2030-2034 | 0 | | 0 | 0 | 0 | 243 | 0 | 243 |
|  | 2035-2039 | 0 | | 0 | 0 | 0 | 300 | 0 | 300 |
| Ovary | 2015-2019 | 0 | | 0 | 0 | 101 | 164 | 211 | 476 |
| C56 | 2020-2024 | 0 | | 0 | 0 | 106 | 164 | 251 | 522 |
|  | 2025-2029 | 0 | | 0 | 0 | 116 | 141 | 310 | 567 |
|  | 2030-2034 | 0 | | 0 | 0 | 115 | 144 | 311 | 571 |
|  | 2035-2039 | 0 | | 0 | 0 | 105 | 155 | 300 | 561 |
| Urinary | 2015-2019 | 0 | | 0 | 0 | 0 | 0 | 221 | 221 |
| bladder | 2020-2024 | 0 | | 0 | 0 | 0 | 0 | 248 | 248 |
| C67 | 2025-2029 | 0 | | 0 | 0 | 0 | 0 | 287 | 287 |
|  | 2030-2034 | 0 | | 0 | 0 | 0 | 0 | 305 | 305 |
|  | 2035-2039 | 0 | | 0 | 0 | 0 | 0 | 318 | 318 |
| Kidney | 2015-2019 | 0 | | 0 | 0 | 0 | 122 | 0 | 122 |
| & ureter | 2020-2024 | 0 | | 0 | 0 | 0 | 140 | 0 | 140 |
| C64-C66, | 2025-2029 | 0 | | 0 | 0 | 0 | 120 | 0 | 120 |
| C68 | 2030-2034 | 0 | | 0 | 0 | 0 | 112 | 0 | 112 |
|  | 2035-2039 | 0 | | 0 | 0 | 0 | 120 | 0 | 120 |
| Thyroid | 2015-2019 | 0 | | 0 | 0 | 0 | 0 | 0 | 0 |
| gland | 2020-2024 | 0 | | 0 | 0 | 0 | 0 | 0 | 0 |
| C73 | 2025-2029 | 0 | | 0 | 0 | 0 | 0 | 0 | 0 |
|  | 2030-2034 | 0 | | 0 | 0 | 0 | 0 | 0 | 0 |
|  | 2035-2039 | 0 | | 0 | 0 | 0 | 0 | 0 | 0 |
| Lymphoma | 2015-2019 | 0 | | 0 | 0 | 0 | 231 | 0 | 231 |
| C81-C85, | 2020-2024 | 0 | | 0 | 0 | 0 | 245 | 0 | 245 |
| C96 | 2025-2029 | 0 | | 0 | 0 | 0 | 220 | 0 | 220 |
|  | 2030-2034 | 0 | | 0 | 0 | 0 | 205 | 0 | 205 |
|  | 2035-2039 | 0 | | 0 | 0 | 0 | 200 | 0 | 200 |
| Myeloma | 2015-2019 | 0 | | 0 | 0 | 0 | 0 | 0 | 0 |
| C88-C90 | 2020-2024 | 0 | | 0 | 0 | 0 | 0 | 0 | 0 |
|  | 2025-2029 | 0 | | 0 | 0 | 0 | 0 | 0 | 0 |
|  | 2030-2034 | 0 | | 0 | 0 | 0 | 0 | 0 | 0 |
|  | 2035-2039 | 0 | | 0 | 0 | 0 | 0 | 0 | 0 |
| Leukemia | 2015-2019 | 73 | | 0 | 0 | 0 | 0 | 1,033 | 1,106 |
| C91-C95 | 2020-2024 | 68 | | 0 | 0 | 0 | 0 | 1,187 | 1,255 |
|  | 2025-2029 | 63 | | 0 | 0 | 0 | 0 | 1,377 | 1,440 |
|  | 2030-2034 | 60 | | 0 | 0 | 0 | 0 | 1,373 | 1,433 |
|  | 2035-2039 | 57 | | 0 | 0 | 0 | 0 | 1,347 | 1,404 |
| Total | 2015-2019 | 73 | | 196 | 342 | 641 | 1,568 | 3,796 | 6,616 |
|  | 2020-2024 | 68 | | 177 | 382 | 706 | 1,644 | 4,391 | 7,368 |
|  | 2025-2029 | 63 | | 157 | 354 | 792 | 1,436 | 5,097 | 7,900 |
|  | 2030-2034 | 60 | | 143 | 287 | 813 | 1,419 | 5,141 | 7,862 |
|  | 2035-2039 | 57 | | 148 | 234 | 733 | 1,531 | 5,022 | 7,726 |
|  |  |  | |  |  |  |  |  |  |
| Left ventricular diastolic dysfunction in male | | | | |  |  |  |  |  |
| Sites | Year | Age groups | | | | | | | |
|  |  | 0-14 | | 15-44 | 45-54 | 55-64 | 65-74 | 75+ | Total |
| Oral cavity | 2015-2019 | 0 | | 0 | 0 | 195 | 1,632 | 0 | 1,827 |
| & pharynx | 2020-2024 | 0 | | 0 | 0 | 195 | 1,699 | 0 | 1,894 |
| C00-C14 | 2025-2029 | 0 | | 0 | 0 | 226 | 1,430 | 0 | 1,656 |
|  | 2030-2034 | 0 | | 0 | 0 | 257 | 1,363 | 0 | 1,620 |
|  | 2035-2039 | 0 | | 0 | 0 | 236 | 1,556 | 0 | 1,792 |
| Esophagus | 2015-2019 | 0 | | 0 | 0 | 84 | 515 | 0 | 599 |
| C15 | 2020-2024 | 0 | | 0 | 0 | 81 | 506 | 0 | 587 |
|  | 2025-2029 | 0 | | 0 | 0 | 90 | 427 | 0 | 517 |
|  | 2030-2034 | 0 | | 0 | 0 | 105 | 423 | 0 | 528 |
|  | 2035-2039 | 0 | | 0 | 0 | 103 | 482 | 0 | 585 |
| Stomach | 2015-2019 | 0 | | 0 | 123 | 692 | 1,877 | 3,564 | 6,256 |
| C16 | 2020-2024 | 0 | | 0 | 131 | 598 | 1,844 | 3,942 | 6,515 |
|  | 2025-2029 | 0 | | 0 | 127 | 583 | 1,467 | 4,542 | 6,719 |
|  | 2030-2034 | 0 | | 0 | 114 | 621 | 1,301 | 4,480 | 6,515 |
|  | 2035-2039 | 0 | | 0 | 100 | 598 | 1,306 | 4,155 | 6,158 |
| Colon | 2015-2019 | 0 | | 141 | 306 | 792 | 3,553 | 2,629 | 7,421 |
| & rectum | 2020-2024 | 0 | | 125 | 339 | 771 | 3,544 | 3,035 | 7,814 |
| C18-C20 | 2025-2029 | 0 | | 122 | 320 | 830 | 2,931 | 3,556 | 7,759 |
|  | 2030-2034 | 0 | | 109 | 273 | 888 | 2,824 | 3,531 | 7,625 |
|  | 2035-2039 | 0 | | 109 | 252 | 821 | 3,039 | 3,365 | 7,587 |
| Liver | 2015-2019 | 0 | | 0 | 0 | 0 | 0 | 1,392 | 1,392 |
| C22 | 2020-2024 | 0 | | 0 | 0 | 0 | 0 | 1,334 | 1,334 |
|  | 2025-2029 | 0 | | 0 | 0 | 0 | 0 | 1,342 | 1,342 |
|  | 2030-2034 | 0 | | 0 | 0 | 0 | 0 | 1,230 | 1,230 |
|  | 2035-2039 | 0 | | 0 | 0 | 0 | 0 | 1,065 | 1,065 |
| Gallbladder | 2015-2019 | 0 | | 0 | 0 | 0 | 444 | 415 | 859 |
| & bile duct | 2020-2024 | 0 | | 0 | 0 | 0 | 444 | 469 | 913 |
| C23-C24 | 2025-2029 | 0 | | 0 | 0 | 0 | 352 | 550 | 902 |
|  | 2030-2034 | 0 | | 0 | 0 | 0 | 321 | 550 | 871 |
|  | 2035-2039 | 0 | | 0 | 0 | 0 | 349 | 518 | 867 |
| Pancreas | 2015-2019 | 0 | | 0 | 0 | 197 | 438 | 500 | 1,136 |
| C25 | 2020-2024 | 0 | | 0 | 0 | 186 | 436 | 595 | 1,217 |
|  | 2025-2029 | 0 | | 0 | 0 | 196 | 353 | 717 | 1,266 |
|  | 2030-2034 | 0 | | 0 | 0 | 204 | 329 | 702 | 1,235 |
|  | 2035-2039 | 0 | | 0 | 0 | 189 | 349 | 645 | 1,183 |
| Larynx | 2015-2019 | 0 | | 0 | 0 | 158 | 117 | 0 | 275 |
| C32 | 2020-2024 | 0 | | 0 | 0 | 141 | 111 | 0 | 253 |
|  | 2025-2029 | 0 | | 0 | 0 | 153 | 89 | 0 | 242 |
|  | 2030-2034 | 0 | | 0 | 0 | 168 | 80 | 0 | 247 |
|  | 2035-2039 | 0 | | 0 | 0 | 190 | 88 | 0 | 278 |
| Lung | 2015-2019 | 0 | | 0 | 0 | 372 | 880 | 743 | 1,995 |
| C33-C34 | 2020-2024 | 0 | | 0 | 0 | 356 | 917 | 870 | 2,143 |
|  | 2025-2029 | 0 | | 0 | 0 | 369 | 770 | 1,053 | 2,192 |
|  | 2030-2034 | 0 | | 0 | 0 | 378 | 719 | 1,061 | 2,158 |
|  | 2035-2039 | 0 | | 0 | 0 | 349 | 746 | 1,003 | 2,097 |
| Skin | 2015-2019 | 0 | | 0 | 0 | 0 | 142 | 266 | 408 |
| C43-C44 | 2020-2024 | 0 | | 0 | 0 | 0 | 166 | 342 | 508 |
|  | 2025-2029 | 0 | | 0 | 0 | 0 | 155 | 419 | 574 |
|  | 2030-2034 | 0 | | 0 | 0 | 0 | 165 | 443 | 608 |
|  | 2035-2039 | 0 | | 0 | 0 | 0 | 193 | 459 | 652 |
| Prostate | 2015-2019 | 0 | | 0 | 0 | 117 | 1,465 | 2,858 | 4,440 |
| C61 | 2020-2024 | 0 | | 0 | 0 | 119 | 1,763 | 4,095 | 5,978 |
|  | 2025-2029 | 0 | | 0 | 0 | 115 | 1,650 | 5,685 | 7,450 |
|  | 2030-2034 | 0 | | 0 | 0 | 101 | 1,609 | 6,494 | 8,204 |
|  | 2035-2039 | 0 | | 0 | 0 | 90 | 1,559 | 6,830 | 8,479 |
| Urinary | 2015-2019 | 0 | | 0 | 0 | 235 | 527 | 2,246 | 3,008 |
| bladder | 2020-2024 | 0 | | 0 | 0 | 216 | 527 | 2,575 | 3,318 |
| C67 | 2025-2029 | 0 | | 0 | 0 | 210 | 417 | 3,093 | 3,720 |
|  | 2030-2034 | 0 | | 0 | 0 | 195 | 389 | 3,208 | 3,792 |
|  | 2035-2039 | 0 | | 0 | 0 | 167 | 387 | 3,106 | 3,660 |
| Kidney | 2015-2019 | 0 | | 0 | 0 | 496 | 861 | 376 | 1,734 |
| & ureter | 2020-2024 | 0 | | 0 | 0 | 552 | 1,007 | 476 | 2,035 |
| C64-C66, | 2025-2029 | 0 | | 0 | 0 | 663 | 927 | 619 | 2,209 |
| C68 | 2030-2034 | 0 | | 0 | 0 | 713 | 941 | 656 | 2,310 |
|  | 2035-2039 | 0 | | 0 | 0 | 631 | 1,081 | 657 | 2,369 |
| Thyroid | 2015-2019 | 0 | | 0 | 0 | 113 | 0 | 0 | 113 |
| gland | 2020-2024 | 0 | | 0 | 0 | 131 | 0 | 0 | 131 |
| C73 | 2025-2029 | 0 | | 0 | 0 | 164 | 0 | 0 | 164 |
|  | 2030-2034 | 0 | | 0 | 0 | 188 | 0 | 0 | 188 |
|  | 2035-2039 | 0 | | 0 | 0 | 182 | 0 | 0 | 182 |
| Lymphoma | 2015-2019 | 0 | | 0 | 0 | 451 | 283 | 390 | 1,125 |
| C81-C85, | 2020-2024 | 0 | | 0 | 0 | 480 | 326 | 486 | 1,292 |
| C96 | 2025-2029 | 0 | | 0 | 0 | 521 | 311 | 607 | 1,439 |
|  | 2030-2034 | 0 | | 0 | 0 | 559 | 305 | 645 | 1,509 |
|  | 2035-2039 | 0 | | 0 | 0 | 535 | 323 | 655 | 1,513 |
| Myeloma | 2015-2019 | 0 | | 0 | 0 | 120 | 373 | 0 | 493 |
| C88-C90 | 2020-2024 | 0 | | 0 | 0 | 119 | 396 | 0 | 515 |
|  | 2025-2029 | 0 | | 0 | 0 | 128 | 330 | 0 | 458 |
|  | 2030-2034 | 0 | | 0 | 0 | 135 | 301 | 0 | 436 |
|  | 2035-2039 | 0 | | 0 | 0 | 120 | 310 | 0 | 430 |
| Leukemia | 2015-2019 | 0 | | 0 | 149 | 0 | 0 | 0 | 149 |
| C91-C95 | 2020-2024 | 0 | | 0 | 181 | 0 | 0 | 0 | 181 |
|  | 2025-2029 | 0 | | 0 | 179 | 0 | 0 | 0 | 179 |
|  | 2030-2034 | 0 | | 0 | 162 | 0 | 0 | 0 | 162 |
|  | 2035-2039 | 0 | | 0 | 161 | 0 | 0 | 0 | 161 |
| Total | 2015-2019 | 0 | | 141 | 579 | 4,023 | 13,107 | 15,378 | 33,229 |
|  | 2020-2024 | 0 | | 125 | 651 | 3,944 | 13,687 | 18,220 | 36,627 |
|  | 2025-2029 | 0 | | 122 | 627 | 4,249 | 11,608 | 22,181 | 38,787 |
|  | 2030-2034 | 0 | | 109 | 548 | 4,510 | 11,072 | 22,998 | 39,238 |
|  | 2035-2039 | 0 | | 109 | 513 | 4,210 | 11,767 | 22,456 | 39,056 |
|  |  |  | |  |  |  |  |  |  |
| Left ventricular diastolic dysfunction in female | | | | |  |  |  |  |  |
| Sites | Year | Age groups | | | | | | | |
|  |  | 0-14 | | 15-44 | 45-54 | 55-64 | 65-74 | 75+ | Total |
| Oral cavity | 2015-2019 | 0 | | 0 | 0 | 0 | 0 | 0 | 0 |
| & pharynx | 2020-2024 | 0 | | 0 | 0 | 0 | 0 | 0 | 0 |
| C00-C14 | 2025-2029 | 0 | | 0 | 0 | 0 | 0 | 0 | 0 |
|  | 2030-2034 | 0 | | 0 | 0 | 0 | 0 | 0 | 0 |
|  | 2035-2039 | 0 | | 0 | 0 | 0 | 0 | 0 | 0 |
| Esophagus | 2015-2019 | 0 | | 0 | 0 | 0 | 0 | 175 | 175 |
| C15 | 2020-2024 | 0 | | 0 | 0 | 0 | 0 | 205 | 205 |
|  | 2025-2029 | 0 | | 0 | 0 | 0 | 0 | 246 | 246 |
|  | 2030-2034 | 0 | | 0 | 0 | 0 | 0 | 255 | 255 |
|  | 2035-2039 | 0 | | 0 | 0 | 0 | 0 | 257 | 257 |
| Stomach | 2015-2019 | 0 | | 0 | 0 | 0 | 592 | 783 | 1,375 |
| C16 | 2020-2024 | 0 | | 0 | 0 | 0 | 584 | 854 | 1,439 |
|  | 2025-2029 | 0 | | 0 | 0 | 0 | 463 | 967 | 1,430 |
|  | 2030-2034 | 0 | | 0 | 0 | 0 | 417 | 971 | 1,388 |
|  | 2035-2039 | 0 | | 0 | 0 | 0 | 413 | 928 | 1,341 |
| Colon | 2015-2019 | 0 | | 0 | 0 | 162 | 828 | 1,914 | 2,905 |
| & rectum | 2020-2024 | 0 | | 0 | 0 | 160 | 831 | 2,201 | 3,192 |
| C18-C20 | 2025-2029 | 0 | | 0 | 0 | 177 | 689 | 2,508 | 3,373 |
|  | 2030-2034 | 0 | | 0 | 0 | 183 | 661 | 2,523 | 3,367 |
|  | 2035-2039 | 0 | | 0 | 0 | 166 | 719 | 2,441 | 3,326 |
| Liver | 2015-2019 | 0 | | 0 | 0 | 0 | 1,818 | 0 | 1,818 |
| C22 | 2020-2024 | 0 | | 0 | 0 | 0 | 1,602 | 0 | 1,602 |
|  | 2025-2029 | 0 | | 0 | 0 | 0 | 1,212 | 0 | 1,212 |
|  | 2030-2034 | 0 | | 0 | 0 | 0 | 1,112 | 0 | 1,112 |
|  | 2035-2039 | 0 | | 0 | 0 | 0 | 1,183 | 0 | 1,183 |
| Gallbladder | 2015-2019 | 0 | | 0 | 0 | 0 | 0 | 842 | 842 |
| & bile duct | 2020-2024 | 0 | | 0 | 0 | 0 | 0 | 884 | 884 |
| C23-C24 | 2025-2029 | 0 | | 0 | 0 | 0 | 0 | 938 | 938 |
|  | 2030-2034 | 0 | | 0 | 0 | 0 | 0 | 908 | 908 |
|  | 2035-2039 | 0 | | 0 | 0 | 0 | 0 | 854 | 854 |
| Pancreas | 2015-2019 | 0 | | 0 | 0 | 188 | 0 | 0 | 188 |
| C25 | 2020-2024 | 0 | | 0 | 0 | 187 | 0 | 0 | 187 |
|  | 2025-2029 | 0 | | 0 | 0 | 213 | 0 | 0 | 213 |
|  | 2030-2034 | 0 | | 0 | 0 | 231 | 0 | 0 | 231 |
|  | 2035-2039 | 0 | | 0 | 0 | 206 | 0 | 0 | 206 |
| Larynx | 2015-2019 | 0 | | 0 | 0 | 0 | 0 | 0 | 0 |
| C32 | 2020-2024 | 0 | | 0 | 0 | 0 | 0 | 0 | 0 |
|  | 2025-2029 | 0 | | 0 | 0 | 0 | 0 | 0 | 0 |
|  | 2030-2034 | 0 | | 0 | 0 | 0 | 0 | 0 | 0 |
|  | 2035-2039 | 0 | | 0 | 0 | 0 | 0 | 0 | 0 |
| Lung | 2015-2019 | 0 | | 0 | 0 | 87 | 401 | 0 | 488 |
| C33-C34 | 2020-2024 | 0 | | 0 | 0 | 86 | 429 | 0 | 515 |
|  | 2025-2029 | 0 | | 0 | 0 | 90 | 359 | 0 | 448 |
|  | 2030-2034 | 0 | | 0 | 0 | 96 | 334 | 0 | 430 |
|  | 2035-2039 | 0 | | 0 | 0 | 92 | 340 | 0 | 431 |
| Skin | 2015-2019 | 0 | | 0 | 0 | 0 | 128 | 0 | 128 |
| C43-C44 | 2020-2024 | 0 | | 0 | 0 | 0 | 148 | 0 | 148 |
|  | 2025-2029 | 0 | | 0 | 0 | 0 | 137 | 0 | 137 |
|  | 2030-2034 | 0 | | 0 | 0 | 0 | 144 | 0 | 144 |
|  | 2035-2039 | 0 | | 0 | 0 | 0 | 160 | 0 | 160 |
| Breast | 2015-2019 | 0 | | 98 | 228 | 566 | 2,289 | 2,059 | 5,240 |
| C50 | 2020-2024 | 0 | | 88 | 255 | 642 | 2,605 | 2,847 | 6,438 |
|  | 2025-2029 | 0 | | 79 | 236 | 733 | 2,439 | 3,857 | 7,344 |
|  | 2030-2034 | 0 | | 71 | 191 | 752 | 2,537 | 4,244 | 7,795 |
|  | 2035-2039 | 0 | | 74 | 156 | 671 | 2,777 | 4,469 | 8,147 |
| Uterus | 2015-2019 | 0 | | 337 | 0 | 313 | 381 | 991 | 2,023 |
| C53-C55 | 2020-2024 | 0 | | 365 | 0 | 395 | 420 | 1,241 | 2,421 |
|  | 2025-2029 | 0 | | 372 | 0 | 504 | 416 | 1,579 | 2,871 |
|  | 2030-2034 | 0 | | 340 | 0 | 570 | 486 | 1,666 | 3,061 |
|  | 2035-2039 | 0 | | 344 | 0 | 580 | 599 | 1,779 | 3,302 |
| Ovary | 2015-2019 | 0 | | 0 | 95 | 0 | 164 | 0 | 258 |
| C56 | 2020-2024 | 0 | | 0 | 98 | 0 | 164 | 0 | 262 |
|  | 2025-2029 | 0 | | 0 | 91 | 0 | 141 | 0 | 231 |
|  | 2030-2034 | 0 | | 0 | 83 | 0 | 144 | 0 | 227 |
|  | 2035-2039 | 0 | | 0 | 78 | 0 | 155 | 0 | 233 |
| Urinary | 2015-2019 | 0 | | 0 | 0 | 0 | 0 | 221 | 221 |
| bladder | 2020-2024 | 0 | | 0 | 0 | 0 | 0 | 248 | 248 |
| C67 | 2025-2029 | 0 | | 0 | 0 | 0 | 0 | 287 | 287 |
|  | 2030-2034 | 0 | | 0 | 0 | 0 | 0 | 305 | 305 |
|  | 2035-2039 | 0 | | 0 | 0 | 0 | 0 | 318 | 318 |
| Kidney | 2015-2019 | 0 | | 0 | 0 | 245 | 0 | 669 | 914 |
| & ureter | 2020-2024 | 0 | | 0 | 0 | 250 | 0 | 800 | 1,050 |
| C64-C66, | 2025-2029 | 0 | | 0 | 0 | 278 | 0 | 975 | 1,253 |
| C68 | 2030-2034 | 0 | | 0 | 0 | 294 | 0 | 1,016 | 1,310 |
|  | 2035-2039 | 0 | | 0 | 0 | 260 | 0 | 1,019 | 1,279 |
| Thyroid | 2015-2019 | 0 | | 0 | 0 | 0 | 0 | 0 | 0 |
| gland | 2020-2024 | 0 | | 0 | 0 | 0 | 0 | 0 | 0 |
| C73 | 2025-2029 | 0 | | 0 | 0 | 0 | 0 | 0 | 0 |
|  | 2030-2034 | 0 | | 0 | 0 | 0 | 0 | 0 | 0 |
|  | 2035-2039 | 0 | | 0 | 0 | 0 | 0 | 0 | 0 |
| Lymphoma | 2015-2019 | 0 | | 0 | 0 | 0 | 461 | 305 | 766 |
| C81-C85, | 2020-2024 | 0 | | 0 | 0 | 0 | 491 | 360 | 851 |
| C96 | 2025-2029 | 0 | | 0 | 0 | 0 | 440 | 426 | 866 |
|  | 2030-2034 | 0 | | 0 | 0 | 0 | 410 | 437 | 847 |
|  | 2035-2039 | 0 | | 0 | 0 | 0 | 401 | 434 | 835 |
| Myeloma | 2015-2019 | 0 | | 0 | 0 | 173 | 221 | 974 | 1,368 |
| C88-C90 | 2020-2024 | 0 | | 0 | 0 | 202 | 232 | 1,170 | 1,604 |
|  | 2025-2029 | 0 | | 0 | 0 | 247 | 208 | 1,360 | 1,815 |
|  | 2030-2034 | 0 | | 0 | 0 | 247 | 219 | 1,400 | 1,866 |
|  | 2035-2039 | 0 | | 0 | 0 | 260 | 251 | 1,362 | 1,873 |
| Leukemia | 2015-2019 | 0 | | 0 | 0 | 0 | 215 | 517 | 731 |
| C91-C95 | 2020-2024 | 0 | | 0 | 0 | 0 | 219 | 593 | 813 |
|  | 2025-2029 | 0 | | 0 | 0 | 0 | 186 | 688 | 874 |
|  | 2030-2034 | 0 | | 0 | 0 | 0 | 178 | 687 | 864 |
|  | 2035-2039 | 0 | | 0 | 0 | 0 | 195 | 673 | 868 |
| Total | 2015-2019 | 0 | | 435 | 323 | 1,735 | 7,498 | 9,449 | 19,440 |
|  | 2020-2024 | 0 | | 453 | 353 | 1,921 | 7,727 | 11,403 | 21,857 |
|  | 2025-2029 | 0 | | 450 | 327 | 2,241 | 6,689 | 13,831 | 23,538 |
|  | 2030-2034 | 0 | | 411 | 274 | 2,372 | 6,641 | 14,411 | 24,109 |
|  | 2035-2039 | 0 | | 418 | 234 | 2,234 | 7,194 | 14,534 | 24,614 |
|  |  |  | |  |  |  |  |  |  |
| Left ventricular dysfunction in male | | | |  |  |  |  |  |  |
| Sites | Year | Age groups | | | | | | | |
|  |  | 0-14 | | 15-44 | 45-54 | 55-64 | 65-74 | 75+ | Total |
| Oral cavity | 2015-2019 | 0 | | 0 | 0 | 584 | 1,632 | 0 | 2,216 |
| & pharynx | 2020-2024 | 0 | | 0 | 0 | 584 | 1,699 | 0 | 2,283 |
| C00-C14 | 2025-2029 | 0 | | 0 | 0 | 679 | 1,430 | 0 | 2,109 |
|  | 2030-2034 | 0 | | 0 | 0 | 771 | 1,363 | 0 | 2,134 |
|  | 2035-2039 | 0 | | 0 | 0 | 707 | 1,556 | 0 | 2,263 |
| Esophagus | 2015-2019 | 0 | | 0 | 0 | 169 | 644 | 659 | 1,471 |
| C15 | 2020-2024 | 0 | | 0 | 0 | 162 | 632 | 790 | 1,583 |
|  | 2025-2029 | 0 | | 0 | 0 | 180 | 533 | 961 | 1,675 |
|  | 2030-2034 | 0 | | 0 | 0 | 210 | 529 | 964 | 1,703 |
|  | 2035-2039 | 0 | | 0 | 0 | 207 | 602 | 918 | 1,726 |
| Stomach | 2015-2019 | 0 | | 0 | 123 | 692 | 3,566 | 5,831 | 10,213 |
| C16 | 2020-2024 | 0 | | 0 | 131 | 598 | 3,503 | 6,450 | 10,683 |
|  | 2025-2029 | 0 | | 0 | 127 | 583 | 2,787 | 7,432 | 10,929 |
|  | 2030-2034 | 0 | | 0 | 114 | 621 | 2,472 | 7,330 | 10,537 |
|  | 2035-2039 | 0 | | 0 | 100 | 598 | 2,481 | 6,799 | 9,977 |
| Colon | 2015-2019 | 0 | | 141 | 459 | 1,109 | 4,522 | 6,834 | 13,066 |
| & rectum | 2020-2024 | 0 | | 125 | 508 | 1,079 | 4,510 | 7,892 | 14,115 |
| C18-C20 | 2025-2029 | 0 | | 122 | 480 | 1,162 | 3,731 | 9,245 | 14,740 |
|  | 2030-2034 | 0 | | 109 | 409 | 1,243 | 3,595 | 9,180 | 14,536 |
|  | 2035-2039 | 0 | | 109 | 378 | 1,150 | 3,868 | 8,749 | 14,254 |
| Liver | 2015-2019 | 0 | | 0 | 0 | 0 | 0 | 2,783 | 2,783 |
| C22 | 2020-2024 | 0 | | 0 | 0 | 0 | 0 | 2,668 | 2,668 |
|  | 2025-2029 | 0 | | 0 | 0 | 0 | 0 | 2,683 | 2,683 |
|  | 2030-2034 | 0 | | 0 | 0 | 0 | 0 | 2,460 | 2,460 |
|  | 2035-2039 | 0 | | 0 | 0 | 0 | 0 | 2,129 | 2,129 |
| Gallbladder | 2015-2019 | 0 | | 0 | 0 | 0 | 444 | 415 | 859 |
| & bile duct | 2020-2024 | 0 | | 0 | 0 | 0 | 444 | 469 | 913 |
| C23-C24 | 2025-2029 | 0 | | 0 | 0 | 0 | 352 | 550 | 902 |
|  | 2030-2034 | 0 | | 0 | 0 | 0 | 321 | 550 | 871 |
|  | 2035-2039 | 0 | | 0 | 0 | 0 | 349 | 518 | 867 |
| Pancreas | 2015-2019 | 0 | | 0 | 0 | 197 | 438 | 500 | 1,136 |
| C25 | 2020-2024 | 0 | | 0 | 0 | 186 | 436 | 595 | 1,217 |
|  | 2025-2029 | 0 | | 0 | 0 | 196 | 353 | 717 | 1,266 |
|  | 2030-2034 | 0 | | 0 | 0 | 204 | 329 | 702 | 1,235 |
|  | 2035-2039 | 0 | | 0 | 0 | 189 | 349 | 645 | 1,183 |
| Larynx | 2015-2019 | 0 | | 0 | 0 | 158 | 350 | 0 | 508 |
| C32 | 2020-2024 | 0 | | 0 | 0 | 141 | 334 | 0 | 476 |
|  | 2025-2029 | 0 | | 0 | 0 | 153 | 267 | 0 | 421 |
|  | 2030-2034 | 0 | | 0 | 0 | 168 | 239 | 0 | 407 |
|  | 2035-2039 | 0 | | 0 | 0 | 190 | 264 | 0 | 454 |
| Lung | 2015-2019 | 0 | | 0 | 204 | 595 | 2,639 | 1,487 | 4,925 |
| C33-C34 | 2020-2024 | 0 | | 0 | 219 | 569 | 2,752 | 1,740 | 5,280 |
|  | 2025-2029 | 0 | | 0 | 206 | 591 | 2,309 | 2,105 | 5,212 |
|  | 2030-2034 | 0 | | 0 | 191 | 605 | 2,156 | 2,123 | 5,075 |
|  | 2035-2039 | 0 | | 0 | 176 | 558 | 2,239 | 2,005 | 4,977 |
| Skin | 2015-2019 | 0 | | 0 | 0 | 0 | 142 | 798 | 940 |
| C43-C44 | 2020-2024 | 0 | | 0 | 0 | 0 | 166 | 1,026 | 1,192 |
|  | 2025-2029 | 0 | | 0 | 0 | 0 | 155 | 1,258 | 1,413 |
|  | 2030-2034 | 0 | | 0 | 0 | 0 | 165 | 1,328 | 1,493 |
|  | 2035-2039 | 0 | | 0 | 0 | 0 | 193 | 1,376 | 1,569 |
| Prostate | 2015-2019 | 0 | | 0 | 0 | 352 | 3,906 | 6,125 | 10,383 |
| C61 | 2020-2024 | 0 | | 0 | 0 | 358 | 4,701 | 8,776 | 13,835 |
|  | 2025-2029 | 0 | | 0 | 0 | 345 | 4,400 | 12,182 | 16,926 |
|  | 2030-2034 | 0 | | 0 | 0 | 303 | 4,291 | 13,916 | 18,510 |
|  | 2035-2039 | 0 | | 0 | 0 | 269 | 4,158 | 14,636 | 19,062 |
| Urinary | 2015-2019 | 0 | | 0 | 57 | 282 | 949 | 3,689 | 4,977 |
| bladder | 2020-2024 | 0 | | 0 | 51 | 259 | 949 | 4,230 | 5,490 |
| C67 | 2025-2029 | 0 | | 0 | 43 | 252 | 751 | 5,081 | 6,127 |
|  | 2030-2034 | 0 | | 0 | 36 | 234 | 701 | 5,270 | 6,241 |
|  | 2035-2039 | 0 | | 0 | 30 | 201 | 696 | 5,103 | 6,029 |
| Kidney | 2015-2019 | 0 | | 0 | 0 | 745 | 1,206 | 753 | 2,703 |
| & ureter | 2020-2024 | 0 | | 0 | 0 | 828 | 1,410 | 952 | 3,191 |
| C64-C66, | 2025-2029 | 0 | | 0 | 0 | 995 | 1,297 | 1,237 | 3,529 |
| C68 | 2030-2034 | 0 | | 0 | 0 | 1,070 | 1,317 | 1,312 | 3,699 |
|  | 2035-2039 | 0 | | 0 | 0 | 947 | 1,513 | 1,314 | 3,774 |
| Thyroid | 2015-2019 | 0 | | 0 | 0 | 226 | 0 | 0 | 226 |
| gland | 2020-2024 | 0 | | 0 | 0 | 262 | 0 | 0 | 262 |
| C73 | 2025-2029 | 0 | | 0 | 0 | 327 | 0 | 0 | 327 |
|  | 2030-2034 | 0 | | 0 | 0 | 375 | 0 | 0 | 375 |
|  | 2035-2039 | 0 | | 0 | 0 | 364 | 0 | 0 | 364 |
| Lymphoma | 2015-2019 | 0 | | 0 | 171 | 451 | 850 | 390 | 1,862 |
| C81-C85, | 2020-2024 | 0 | | 0 | 203 | 480 | 978 | 486 | 2,147 |
| C96 | 2025-2029 | 0 | | 0 | 205 | 521 | 933 | 607 | 2,267 |
|  | 2030-2034 | 0 | | 0 | 185 | 559 | 916 | 645 | 2,304 |
|  | 2035-2039 | 0 | | 0 | 166 | 535 | 968 | 655 | 2,324 |
| Myeloma | 2015-2019 | 0 | | 0 | 0 | 480 | 560 | 0 | 1,040 |
| C88-C90 | 2020-2024 | 0 | | 0 | 0 | 477 | 594 | 0 | 1,071 |
|  | 2025-2029 | 0 | | 0 | 0 | 512 | 495 | 0 | 1,007 |
|  | 2030-2034 | 0 | | 0 | 0 | 539 | 452 | 0 | 990 |
|  | 2035-2039 | 0 | | 0 | 0 | 480 | 465 | 0 | 945 |
| Leukemia | 2015-2019 | 78 | | 0 | 298 | 141 | 675 | 0 | 1,192 |
| C91-C95 | 2020-2024 | 73 | | 0 | 362 | 145 | 688 | 0 | 1,268 |
|  | 2025-2029 | 68 | | 0 | 358 | 159 | 578 | 0 | 1,165 |
|  | 2030-2034 | 65 | | 0 | 325 | 182 | 572 | 0 | 1,143 |
|  | 2035-2039 | 62 | | 0 | 322 | 174 | 618 | 0 | 1,177 |
| Total | 2015-2019 | 78 | | 141 | 1,312 | 6,180 | 22,524 | 30,264 | 60,500 |
|  | 2020-2024 | 73 | | 125 | 1,474 | 6,127 | 23,799 | 36,075 | 67,673 |
|  | 2025-2029 | 68 | | 122 | 1,421 | 6,655 | 20,372 | 44,058 | 72,697 |
|  | 2030-2034 | 65 | | 109 | 1,258 | 7,083 | 19,419 | 45,779 | 73,712 |
|  | 2035-2039 | 62 | | 109 | 1,171 | 6,567 | 20,319 | 44,846 | 73,074 |
|  |  |  | |  |  |  |  |  |  |
| Left ventricular dysfunction in female | | | |  |  |  |  |  |  |
| Sites | Year | Age groups | | | | | | | |
|  |  | 0-14 | | 15-44 | 45-54 | 55-64 | 65-74 | 75+ | Total |
| Oral cavity | 2015-2019 | 0 | | 0 | 0 | 0 | 0 | 0 | 0 |
| & pharynx | 2020-2024 | 0 | | 0 | 0 | 0 | 0 | 0 | 0 |
| C00-C14 | 2025-2029 | 0 | | 0 | 0 | 0 | 0 | 0 | 0 |
|  | 2030-2034 | 0 | | 0 | 0 | 0 | 0 | 0 | 0 |
|  | 2035-2039 | 0 | | 0 | 0 | 0 | 0 | 0 | 0 |
| Esophagus | 2015-2019 | 0 | | 0 | 0 | 0 | 0 | 349 | 349 |
| C15 | 2020-2024 | 0 | | 0 | 0 | 0 | 0 | 410 | 410 |
|  | 2025-2029 | 0 | | 0 | 0 | 0 | 0 | 491 | 491 |
|  | 2030-2034 | 0 | | 0 | 0 | 0 | 0 | 510 | 510 |
|  | 2035-2039 | 0 | | 0 | 0 | 0 | 0 | 513 | 513 |
| Stomach | 2015-2019 | 0 | | 0 | 0 | 0 | 740 | 783 | 1,523 |
| C16 | 2020-2024 | 0 | | 0 | 0 | 0 | 731 | 854 | 1,585 |
|  | 2025-2029 | 0 | | 0 | 0 | 0 | 578 | 967 | 1,546 |
|  | 2030-2034 | 0 | | 0 | 0 | 0 | 521 | 971 | 1,492 |
|  | 2035-2039 | 0 | | 0 | 0 | 0 | 517 | 928 | 1,445 |
| Colon | 2015-2019 | 0 | | 0 | 0 | 162 | 1,105 | 3,828 | 5,095 |
| & rectum | 2020-2024 | 0 | | 0 | 0 | 160 | 1,108 | 4,402 | 5,670 |
| C18-C20 | 2025-2029 | 0 | | 0 | 0 | 177 | 919 | 5,015 | 6,110 |
|  | 2030-2034 | 0 | | 0 | 0 | 183 | 882 | 5,046 | 6,111 |
|  | 2035-2039 | 0 | | 0 | 0 | 166 | 959 | 4,882 | 6,007 |
| Liver | 2015-2019 | 0 | | 0 | 0 | 0 | 1,818 | 0 | 1,818 |
| C22 | 2020-2024 | 0 | | 0 | 0 | 0 | 1,602 | 0 | 1,602 |
|  | 2025-2029 | 0 | | 0 | 0 | 0 | 1,212 | 0 | 1,212 |
|  | 2030-2034 | 0 | | 0 | 0 | 0 | 1,112 | 0 | 1,112 |
|  | 2035-2039 | 0 | | 0 | 0 | 0 | 1,183 | 0 | 1,183 |
| Gallbladder | 2015-2019 | 0 | | 0 | 0 | 0 | 209 | 842 | 1,050 |
| & bile duct | 2020-2024 | 0 | | 0 | 0 | 0 | 200 | 884 | 1,085 |
| C23-C24 | 2025-2029 | 0 | | 0 | 0 | 0 | 158 | 938 | 1,097 |
|  | 2030-2034 | 0 | | 0 | 0 | 0 | 137 | 908 | 1,044 |
|  | 2035-2039 | 0 | | 0 | 0 | 0 | 135 | 854 | 989 |
| Pancreas | 2015-2019 | 0 | | 0 | 0 | 188 | 0 | 0 | 188 |
| C25 | 2020-2024 | 0 | | 0 | 0 | 187 | 0 | 0 | 187 |
|  | 2025-2029 | 0 | | 0 | 0 | 213 | 0 | 0 | 213 |
|  | 2030-2034 | 0 | | 0 | 0 | 231 | 0 | 0 | 231 |
|  | 2035-2039 | 0 | | 0 | 0 | 206 | 0 | 0 | 206 |
| Larynx | 2015-2019 | 0 | | 0 | 0 | 0 | 0 | 0 | 0 |
| C32 | 2020-2024 | 0 | | 0 | 0 | 0 | 0 | 0 | 0 |
|  | 2025-2029 | 0 | | 0 | 0 | 0 | 0 | 0 | 0 |
|  | 2030-2034 | 0 | | 0 | 0 | 0 | 0 | 0 | 0 |
|  | 2035-2039 | 0 | | 0 | 0 | 0 | 0 | 0 | 0 |
| Lung | 2015-2019 | 0 | | 0 | 0 | 174 | 401 | 242 | 817 |
| C33-C34 | 2020-2024 | 0 | | 0 | 0 | 172 | 429 | 300 | 901 |
|  | 2025-2029 | 0 | | 0 | 0 | 179 | 359 | 370 | 907 |
|  | 2030-2034 | 0 | | 0 | 0 | 191 | 334 | 373 | 899 |
|  | 2035-2039 | 0 | | 0 | 0 | 184 | 340 | 358 | 882 |
| Skin | 2015-2019 | 0 | | 0 | 0 | 0 | 128 | 0 | 128 |
| C43-C44 | 2020-2024 | 0 | | 0 | 0 | 0 | 148 | 0 | 148 |
|  | 2025-2029 | 0 | | 0 | 0 | 0 | 137 | 0 | 137 |
|  | 2030-2034 | 0 | | 0 | 0 | 0 | 144 | 0 | 144 |
|  | 2035-2039 | 0 | | 0 | 0 | 0 | 160 | 0 | 160 |
| Breast | 2015-2019 | 0 | | 293 | 571 | 1,019 | 2,518 | 2,059 | 6,460 |
| C50 | 2020-2024 | 0 | | 265 | 637 | 1,155 | 2,866 | 2,847 | 7,771 |
|  | 2025-2029 | 0 | | 236 | 590 | 1,320 | 2,683 | 3,857 | 8,685 |
|  | 2030-2034 | 0 | | 214 | 478 | 1,353 | 2,790 | 4,244 | 9,080 |
|  | 2035-2039 | 0 | | 221 | 391 | 1,207 | 3,055 | 4,469 | 9,343 |
| Uterus | 2015-2019 | 0 | | 337 | 0 | 313 | 572 | 991 | 2,213 |
| C53-C55 | 2020-2024 | 0 | | 365 | 0 | 395 | 631 | 1,241 | 2,631 |
|  | 2025-2029 | 0 | | 372 | 0 | 504 | 625 | 1,579 | 3,080 |
|  | 2030-2034 | 0 | | 340 | 0 | 570 | 728 | 1,666 | 3,304 |
|  | 2035-2039 | 0 | | 344 | 0 | 580 | 899 | 1,779 | 3,602 |
| Ovary | 2015-2019 | 0 | | 0 | 95 | 101 | 327 | 211 | 734 |
| C56 | 2020-2024 | 0 | | 0 | 98 | 106 | 329 | 251 | 784 |
|  | 2025-2029 | 0 | | 0 | 91 | 116 | 281 | 310 | 799 |
|  | 2030-2034 | 0 | | 0 | 83 | 115 | 288 | 311 | 798 |
|  | 2035-2039 | 0 | | 0 | 78 | 105 | 311 | 300 | 794 |
| Urinary | 2015-2019 | 0 | | 0 | 0 | 0 | 0 | 441 | 441 |
| bladder | 2020-2024 | 0 | | 0 | 0 | 0 | 0 | 496 | 496 |
| C67 | 2025-2029 | 0 | | 0 | 0 | 0 | 0 | 574 | 574 |
|  | 2030-2034 | 0 | | 0 | 0 | 0 | 0 | 610 | 610 |
|  | 2035-2039 | 0 | | 0 | 0 | 0 | 0 | 637 | 637 |
| Kidney | 2015-2019 | 0 | | 0 | 0 | 245 | 122 | 669 | 1,036 |
| & ureter | 2020-2024 | 0 | | 0 | 0 | 250 | 140 | 800 | 1,189 |
| C64-C66, | 2025-2029 | 0 | | 0 | 0 | 278 | 120 | 975 | 1,373 |
| C68 | 2030-2034 | 0 | | 0 | 0 | 294 | 112 | 1,016 | 1,422 |
|  | 2035-2039 | 0 | | 0 | 0 | 260 | 120 | 1,019 | 1,399 |
| Thyroid | 2015-2019 | 0 | | 0 | 0 | 0 | 0 | 0 | 0 |
| gland | 2020-2024 | 0 | | 0 | 0 | 0 | 0 | 0 | 0 |
| C73 | 2025-2029 | 0 | | 0 | 0 | 0 | 0 | 0 | 0 |
|  | 2030-2034 | 0 | | 0 | 0 | 0 | 0 | 0 | 0 |
|  | 2035-2039 | 0 | | 0 | 0 | 0 | 0 | 0 | 0 |
| Lymphoma | 2015-2019 | 0 | | 0 | 0 | 0 | 692 | 305 | 997 |
| C81-C85, | 2020-2024 | 0 | | 0 | 0 | 0 | 736 | 360 | 1,096 |
| C96 | 2025-2029 | 0 | | 0 | 0 | 0 | 661 | 426 | 1,086 |
|  | 2030-2034 | 0 | | 0 | 0 | 0 | 615 | 437 | 1,052 |
|  | 2035-2039 | 0 | | 0 | 0 | 0 | 601 | 434 | 1,035 |
| Myeloma | 2015-2019 | 0 | | 0 | 0 | 173 | 221 | 974 | 1,368 |
| C88-C90 | 2020-2024 | 0 | | 0 | 0 | 202 | 232 | 1,170 | 1,604 |
|  | 2025-2029 | 0 | | 0 | 0 | 247 | 208 | 1,360 | 1,815 |
|  | 2030-2034 | 0 | | 0 | 0 | 247 | 219 | 1,400 | 1,866 |
|  | 2035-2039 | 0 | | 0 | 0 | 260 | 251 | 1,362 | 1,873 |
| Leukemia | 2015-2019 | 73 | | 0 | 0 | 0 | 215 | 1,550 | 1,837 |
| C91-C95 | 2020-2024 | 68 | | 0 | 0 | 0 | 219 | 1,780 | 2,067 |
|  | 2025-2029 | 63 | | 0 | 0 | 0 | 186 | 2,065 | 2,314 |
|  | 2030-2034 | 60 | | 0 | 0 | 0 | 178 | 2,060 | 2,298 |
|  | 2035-2039 | 57 | | 0 | 0 | 0 | 195 | 2,020 | 2,272 |
| Total | 2015-2019 | 73 | | 631 | 665 | 2,375 | 9,067 | 13,245 | 26,056 |
|  | 2020-2024 | 68 | | 630 | 735 | 2,627 | 9,371 | 15,795 | 29,225 |
|  | 2025-2029 | 63 | | 607 | 681 | 3,033 | 8,126 | 18,927 | 31,438 |
|  | 2030-2034 | 60 | | 554 | 561 | 3,185 | 8,059 | 19,552 | 31,971 |
|  | 2035-2039 | 57 | | 566 | 468 | 2,967 | 8,725 | 19,555 | 32,340 |
|  |  |  | |  |  |  |  |  |  |
| Atrial fibrillation in male | | |  |  |  |  |  |  |  |
| Sites | Year | Age groups | | | | | | | |
|  |  | 0-14 | | 15-44 | 45-54 | 55-64 | 65-74 | 75+ | Total |
| Oral cavity | 2015-2019 | 0 | | 0 | 0 | 195 | 1,306 | 701 | 2,201 |
| & pharynx | 2020-2024 | 0 | | 0 | 0 | 195 | 1,359 | 871 | 2,424 |
| C00-C14 | 2025-2029 | 0 | | 0 | 0 | 226 | 1,144 | 1,092 | 2,462 |
|  | 2030-2034 | 0 | | 0 | 0 | 257 | 1,090 | 1,083 | 2,430 |
|  | 2035-2039 | 0 | | 0 | 0 | 236 | 1,245 | 1,031 | 2,511 |
| Esophagus | 2015-2019 | 0 | | 0 | 0 | 253 | 1,545 | 878 | 2,676 |
| C15 | 2020-2024 | 0 | | 0 | 0 | 243 | 1,517 | 1,053 | 2,812 |
|  | 2025-2029 | 0 | | 0 | 0 | 270 | 1,280 | 1,282 | 2,832 |
|  | 2030-2034 | 0 | | 0 | 0 | 315 | 1,270 | 1,285 | 2,870 |
|  | 2035-2039 | 0 | | 0 | 0 | 310 | 1,445 | 1,224 | 2,979 |
| Stomach | 2015-2019 | 0 | | 0 | 0 | 1,088 | 4,880 | 8,423 | 14,391 |
| C16 | 2020-2024 | 0 | | 0 | 0 | 940 | 4,794 | 9,317 | 15,051 |
|  | 2025-2029 | 0 | | 0 | 0 | 916 | 3,813 | 10,734 | 15,464 |
|  | 2030-2034 | 0 | | 0 | 0 | 975 | 3,383 | 10,588 | 14,947 |
|  | 2035-2039 | 0 | | 0 | 0 | 939 | 3,395 | 9,821 | 14,154 |
| Colon | 2015-2019 | 0 | | 0 | 306 | 1,109 | 5,815 | 8,412 | 15,641 |
| & rectum | 2020-2024 | 0 | | 0 | 339 | 1,079 | 5,799 | 9,713 | 16,930 |
| C18-C20 | 2025-2029 | 0 | | 0 | 320 | 1,162 | 4,796 | 11,379 | 17,658 |
|  | 2030-2034 | 0 | | 0 | 273 | 1,243 | 4,622 | 11,299 | 17,436 |
|  | 2035-2039 | 0 | | 0 | 252 | 1,150 | 4,973 | 10,768 | 17,143 |
| Liver | 2015-2019 | 0 | | 0 | 0 | 696 | 3,129 | 5,566 | 9,391 |
| C22 | 2020-2024 | 0 | | 0 | 0 | 588 | 2,741 | 5,337 | 8,666 |
|  | 2025-2029 | 0 | | 0 | 0 | 599 | 2,043 | 5,366 | 8,008 |
|  | 2030-2034 | 0 | | 0 | 0 | 642 | 1,830 | 4,920 | 7,392 |
|  | 2035-2039 | 0 | | 0 | 0 | 605 | 1,928 | 4,259 | 6,791 |
| Gallbladder | 2015-2019 | 0 | | 0 | 0 | 140 | 0 | 1,244 | 1,383 |
| & bile duct | 2020-2024 | 0 | | 0 | 0 | 127 | 0 | 1,408 | 1,535 |
| C23-C24 | 2025-2029 | 0 | | 0 | 0 | 137 | 0 | 1,651 | 1,788 |
|  | 2030-2034 | 0 | | 0 | 0 | 153 | 0 | 1,649 | 1,802 |
|  | 2035-2039 | 0 | | 0 | 0 | 147 | 0 | 1,553 | 1,700 |
| Pancreas | 2015-2019 | 0 | | 55 | 0 | 394 | 438 | 1,500 | 2,388 |
| C25 | 2020-2024 | 0 | | 50 | 0 | 371 | 436 | 1,786 | 2,643 |
|  | 2025-2029 | 0 | | 37 | 0 | 391 | 353 | 2,151 | 2,932 |
|  | 2030-2034 | 0 | | 30 | 0 | 408 | 329 | 2,105 | 2,872 |
|  | 2035-2039 | 0 | | 30 | 0 | 379 | 349 | 1,934 | 2,692 |
| Larynx | 2015-2019 | 0 | | 0 | 0 | 158 | 350 | 0 | 508 |
| C32 | 2020-2024 | 0 | | 0 | 0 | 141 | 334 | 0 | 476 |
|  | 2025-2029 | 0 | | 0 | 0 | 153 | 267 | 0 | 421 |
|  | 2030-2034 | 0 | | 0 | 0 | 168 | 239 | 0 | 407 |
|  | 2035-2039 | 0 | | 0 | 0 | 190 | 264 | 0 | 454 |
| Lung | 2015-2019 | 0 | | 0 | 204 | 1,041 | 3,666 | 4,460 | 9,371 |
| C33-C34 | 2020-2024 | 0 | | 0 | 219 | 996 | 3,822 | 5,221 | 10,257 |
|  | 2025-2029 | 0 | | 0 | 206 | 1,034 | 3,207 | 6,315 | 10,763 |
|  | 2030-2034 | 0 | | 0 | 191 | 1,059 | 2,995 | 6,368 | 10,612 |
|  | 2035-2039 | 0 | | 0 | 176 | 976 | 3,109 | 6,015 | 10,277 |
| Skin | 2015-2019 | 0 | | 0 | 0 | 189 | 425 | 1,596 | 2,210 |
| C43-C44 | 2020-2024 | 0 | | 0 | 0 | 235 | 498 | 2,053 | 2,786 |
|  | 2025-2029 | 0 | | 0 | 0 | 298 | 464 | 2,516 | 3,278 |
|  | 2030-2034 | 0 | | 0 | 0 | 362 | 495 | 2,655 | 3,512 |
|  | 2035-2039 | 0 | | 0 | 0 | 354 | 580 | 2,751 | 3,686 |
| Prostate | 2015-2019 | 0 | | 0 | 0 | 704 | 7,324 | 10,208 | 18,236 |
| C61 | 2020-2024 | 0 | | 0 | 0 | 715 | 8,815 | 14,627 | 24,157 |
|  | 2025-2029 | 0 | | 0 | 0 | 689 | 8,250 | 20,303 | 29,242 |
|  | 2030-2034 | 0 | | 0 | 0 | 607 | 8,046 | 23,193 | 31,845 |
|  | 2035-2039 | 0 | | 0 | 0 | 537 | 7,795 | 24,393 | 32,726 |
| Urinary | 2015-2019 | 0 | | 0 | 57 | 188 | 1,476 | 2,727 | 4,448 |
| bladder | 2020-2024 | 0 | | 0 | 51 | 173 | 1,477 | 3,127 | 4,827 |
| C67 | 2025-2029 | 0 | | 0 | 43 | 168 | 1,169 | 3,755 | 5,135 |
|  | 2030-2034 | 0 | | 0 | 36 | 156 | 1,090 | 3,896 | 5,178 |
|  | 2035-2039 | 0 | | 0 | 30 | 134 | 1,082 | 3,772 | 5,017 |
| Kidney | 2015-2019 | 0 | | 0 | 255 | 621 | 1,378 | 376 | 2,630 |
| & ureter | 2020-2024 | 0 | | 0 | 311 | 690 | 1,612 | 476 | 3,089 |
| C64-C66, | 2025-2029 | 0 | | 0 | 289 | 829 | 1,483 | 619 | 3,220 |
| C68 | 2030-2034 | 0 | | 0 | 216 | 892 | 1,505 | 656 | 3,269 |
|  | 2035-2039 | 0 | | 0 | 158 | 789 | 1,729 | 657 | 3,333 |
| Thyroid | 2015-2019 | 0 | | 0 | 0 | 113 | 476 | 0 | 589 |
| gland | 2020-2024 | 0 | | 0 | 0 | 131 | 546 | 0 | 677 |
| C73 | 2025-2029 | 0 | | 0 | 0 | 164 | 499 | 0 | 663 |
|  | 2030-2034 | 0 | | 0 | 0 | 188 | 525 | 0 | 712 |
|  | 2035-2039 | 0 | | 0 | 0 | 182 | 625 | 0 | 807 |
| Lymphoma | 2015-2019 | 39 | | 0 | 341 | 451 | 850 | 390 | 2,072 |
| C81-C85, | 2020-2024 | 37 | | 0 | 406 | 480 | 978 | 486 | 2,387 |
| C96 | 2025-2029 | 35 | | 0 | 411 | 521 | 933 | 607 | 2,507 |
|  | 2030-2034 | 33 | | 0 | 370 | 559 | 916 | 645 | 2,522 |
|  | 2035-2039 | 31 | | 0 | 332 | 535 | 968 | 655 | 2,521 |
| Myeloma | 2015-2019 | 0 | | 0 | 0 | 120 | 373 | 566 | 1,059 |
| C88-C90 | 2020-2024 | 0 | | 0 | 0 | 119 | 396 | 676 | 1,191 |
|  | 2025-2029 | 0 | | 0 | 0 | 128 | 330 | 796 | 1,254 |
|  | 2030-2034 | 0 | | 0 | 0 | 135 | 301 | 791 | 1,227 |
|  | 2035-2039 | 0 | | 0 | 0 | 120 | 310 | 720 | 1,150 |
| Leukemia | 2015-2019 | 0 | | 0 | 0 | 281 | 0 | 0 | 281 |
| C91-C95 | 2020-2024 | 0 | | 0 | 0 | 290 | 0 | 0 | 290 |
|  | 2025-2029 | 0 | | 0 | 0 | 319 | 0 | 0 | 319 |
|  | 2030-2034 | 0 | | 0 | 0 | 363 | 0 | 0 | 363 |
|  | 2035-2039 | 0 | | 0 | 0 | 349 | 0 | 0 | 349 |
| Total | 2015-2019 | 39 | | 55 | 1,163 | 7,740 | 33,431 | 47,048 | 89,476 |
|  | 2020-2024 | 37 | | 50 | 1,326 | 7,512 | 35,125 | 56,148 | 100,198 |
|  | 2025-2029 | 35 | | 37 | 1,270 | 8,004 | 30,032 | 68,566 | 107,944 |
|  | 2030-2034 | 33 | | 30 | 1,086 | 8,480 | 28,637 | 71,132 | 109,398 |
|  | 2035-2039 | 31 | | 30 | 947 | 7,930 | 29,799 | 69,553 | 108,290 |
|  |  |  | |  |  |  |  |  |  |
| Atrial fibrillation in female | | |  |  |  |  |  |  |  |
| Sites | Year | Age groups | | | | | | | |
|  |  | 0-14 | | 15-44 | 45-54 | 55-64 | 65-74 | 75+ | Total |
| Oral cavity | 2015-2019 | 0 | | 0 | 0 | 0 | 0 | 0 | 0 |
| & pharynx | 2020-2024 | 0 | | 0 | 0 | 0 | 0 | 0 | 0 |
| C00-C14 | 2025-2029 | 0 | | 0 | 0 | 0 | 0 | 0 | 0 |
|  | 2030-2034 | 0 | | 0 | 0 | 0 | 0 | 0 | 0 |
|  | 2035-2039 | 0 | | 0 | 0 | 0 | 0 | 0 | 0 |
| Esophagus | 2015-2019 | 0 | | 0 | 0 | 0 | 245 | 349 | 594 |
| C15 | 2020-2024 | 0 | | 0 | 0 | 0 | 248 | 410 | 658 |
|  | 2025-2029 | 0 | | 0 | 0 | 0 | 214 | 491 | 705 |
|  | 2030-2034 | 0 | | 0 | 0 | 0 | 228 | 510 | 738 |
|  | 2035-2039 | 0 | | 0 | 0 | 0 | 252 | 513 | 765 |
| Stomach | 2015-2019 | 0 | | 0 | 0 | 0 | 148 | 1,567 | 1,715 |
| C16 | 2020-2024 | 0 | | 0 | 0 | 0 | 146 | 1,708 | 1,855 |
|  | 2025-2029 | 0 | | 0 | 0 | 0 | 116 | 1,935 | 2,050 |
|  | 2030-2034 | 0 | | 0 | 0 | 0 | 104 | 1,943 | 2,047 |
|  | 2035-2039 | 0 | | 0 | 0 | 0 | 103 | 1,856 | 1,959 |
| Colon | 2015-2019 | 0 | | 0 | 0 | 162 | 1,657 | 2,552 | 4,371 |
| & rectum | 2020-2024 | 0 | | 0 | 0 | 160 | 1,662 | 2,935 | 4,756 |
| C18-C20 | 2025-2029 | 0 | | 0 | 0 | 177 | 1,378 | 3,343 | 4,898 |
|  | 2030-2034 | 0 | | 0 | 0 | 183 | 1,323 | 3,364 | 4,870 |
|  | 2035-2039 | 0 | | 0 | 0 | 166 | 1,439 | 3,255 | 4,859 |
| Liver | 2015-2019 | 0 | | 0 | 0 | 0 | 0 | 0 | 0 |
| C22 | 2020-2024 | 0 | | 0 | 0 | 0 | 0 | 0 | 0 |
|  | 2025-2029 | 0 | | 0 | 0 | 0 | 0 | 0 | 0 |
|  | 2030-2034 | 0 | | 0 | 0 | 0 | 0 | 0 | 0 |
|  | 2035-2039 | 0 | | 0 | 0 | 0 | 0 | 0 | 0 |
| Gallbladder | 2015-2019 | 0 | | 0 | 0 | 0 | 209 | 842 | 1,050 |
| & bile duct | 2020-2024 | 0 | | 0 | 0 | 0 | 200 | 884 | 1,085 |
| C23-C24 | 2025-2029 | 0 | | 0 | 0 | 0 | 158 | 938 | 1,097 |
|  | 2030-2034 | 0 | | 0 | 0 | 0 | 137 | 908 | 1,044 |
|  | 2035-2039 | 0 | | 0 | 0 | 0 | 135 | 854 | 989 |
| Pancreas | 2015-2019 | 0 | | 0 | 0 | 0 | 230 | 0 | 230 |
| C25 | 2020-2024 | 0 | | 0 | 0 | 0 | 245 | 0 | 245 |
|  | 2025-2029 | 0 | | 0 | 0 | 0 | 209 | 0 | 209 |
|  | 2030-2034 | 0 | | 0 | 0 | 0 | 197 | 0 | 197 |
|  | 2035-2039 | 0 | | 0 | 0 | 0 | 217 | 0 | 217 |
| Larynx | 2015-2019 | 0 | | 0 | 0 | 0 | 75 | 0 | 75 |
| C32 | 2020-2024 | 0 | | 0 | 0 | 0 | 70 | 0 | 70 |
|  | 2025-2029 | 0 | | 0 | 0 | 0 | 75 | 0 | 75 |
|  | 2030-2034 | 0 | | 0 | 0 | 0 | 67 | 0 | 67 |
|  | 2035-2039 | 0 | | 0 | 0 | 0 | 75 | 0 | 75 |
| Lung | 2015-2019 | 0 | | 0 | 0 | 174 | 1,202 | 1,212 | 2,588 |
| C33-C34 | 2020-2024 | 0 | | 0 | 0 | 172 | 1,287 | 1,499 | 2,958 |
|  | 2025-2029 | 0 | | 0 | 0 | 179 | 1,076 | 1,849 | 3,103 |
|  | 2030-2034 | 0 | | 0 | 0 | 191 | 1,002 | 1,866 | 3,060 |
|  | 2035-2039 | 0 | | 0 | 0 | 184 | 1,019 | 1,792 | 2,995 |
| Skin | 2015-2019 | 0 | | 0 | 0 | 93 | 257 | 259 | 609 |
| C43-C44 | 2020-2024 | 0 | | 0 | 0 | 112 | 297 | 334 | 743 |
|  | 2025-2029 | 0 | | 0 | 0 | 133 | 274 | 404 | 811 |
|  | 2030-2034 | 0 | | 0 | 0 | 151 | 288 | 439 | 878 |
|  | 2035-2039 | 0 | | 0 | 0 | 165 | 319 | 471 | 955 |
| Breast | 2015-2019 | 0 | | 0 | 114 | 226 | 3,205 | 1,544 | 5,090 |
| C50 | 2020-2024 | 0 | | 0 | 127 | 257 | 3,648 | 2,135 | 6,167 |
|  | 2025-2029 | 0 | | 0 | 118 | 293 | 3,415 | 2,893 | 6,719 |
|  | 2030-2034 | 0 | | 0 | 96 | 301 | 3,551 | 3,183 | 7,130 |
|  | 2035-2039 | 0 | | 0 | 78 | 268 | 3,888 | 3,352 | 7,587 |
| Uterus | 2015-2019 | 0 | | 0 | 0 | 104 | 572 | 1,239 | 1,914 |
| C53-C55 | 2020-2024 | 0 | | 0 | 0 | 132 | 631 | 1,551 | 2,313 |
|  | 2025-2029 | 0 | | 0 | 0 | 168 | 625 | 1,974 | 2,766 |
|  | 2030-2034 | 0 | | 0 | 0 | 190 | 728 | 2,082 | 3,000 |
|  | 2035-2039 | 0 | | 0 | 0 | 193 | 899 | 2,224 | 3,316 |
| Ovary | 2015-2019 | 0 | | 0 | 95 | 0 | 164 | 211 | 470 |
| C56 | 2020-2024 | 0 | | 0 | 98 | 0 | 164 | 251 | 513 |
|  | 2025-2029 | 0 | | 0 | 91 | 0 | 141 | 310 | 542 |
|  | 2030-2034 | 0 | | 0 | 83 | 0 | 144 | 311 | 538 |
|  | 2035-2039 | 0 | | 0 | 78 | 0 | 155 | 300 | 534 |
| Urinary | 2015-2019 | 0 | | 0 | 0 | 0 | 95 | 221 | 316 |
| bladder | 2020-2024 | 0 | | 0 | 0 | 0 | 103 | 248 | 351 |
| C67 | 2025-2029 | 0 | | 0 | 0 | 0 | 85 | 287 | 372 |
|  | 2030-2034 | 0 | | 0 | 0 | 0 | 82 | 305 | 388 |
|  | 2035-2039 | 0 | | 0 | 0 | 0 | 89 | 318 | 407 |
| Kidney | 2015-2019 | 0 | | 0 | 0 | 0 | 0 | 334 | 334 |
| & ureter | 2020-2024 | 0 | | 0 | 0 | 0 | 0 | 400 | 400 |
| C64-C66, | 2025-2029 | 0 | | 0 | 0 | 0 | 0 | 488 | 488 |
| C68 | 2030-2034 | 0 | | 0 | 0 | 0 | 0 | 508 | 508 |
|  | 2035-2039 | 0 | | 0 | 0 | 0 | 0 | 509 | 509 |
| Thyroid | 2015-2019 | 0 | | 0 | 0 | 0 | 210 | 432 | 641 |
| gland | 2020-2024 | 0 | | 0 | 0 | 0 | 215 | 546 | 761 |
| C73 | 2025-2029 | 0 | | 0 | 0 | 0 | 190 | 679 | 869 |
|  | 2030-2034 | 0 | | 0 | 0 | 0 | 194 | 683 | 877 |
|  | 2035-2039 | 0 | | 0 | 0 | 0 | 219 | 664 | 883 |
| Lymphoma | 2015-2019 | 60 | | 0 | 0 | 109 | 692 | 610 | 1,471 |
| C81-C85, | 2020-2024 | 55 | | 0 | 0 | 107 | 736 | 720 | 1,618 |
| C96 | 2025-2029 | 53 | | 0 | 0 | 106 | 661 | 851 | 1,670 |
|  | 2030-2034 | 50 | | 0 | 0 | 114 | 615 | 874 | 1,653 |
|  | 2035-2039 | 48 | | 0 | 0 | 116 | 601 | 868 | 1,633 |
| Myeloma | 2015-2019 | 0 | | 0 | 0 | 0 | 0 | 0 | 0 |
| C88-C90 | 2020-2024 | 0 | | 0 | 0 | 0 | 0 | 0 | 0 |
|  | 2025-2029 | 0 | | 0 | 0 | 0 | 0 | 0 | 0 |
|  | 2030-2034 | 0 | | 0 | 0 | 0 | 0 | 0 | 0 |
|  | 2035-2039 | 0 | | 0 | 0 | 0 | 0 | 0 | 0 |
| Leukemia | 2015-2019 | 0 | | 0 | 0 | 0 | 0 | 517 | 517 |
| C91-C95 | 2020-2024 | 0 | | 0 | 0 | 0 | 0 | 593 | 593 |
|  | 2025-2029 | 0 | | 0 | 0 | 0 | 0 | 688 | 688 |
|  | 2030-2034 | 0 | | 0 | 0 | 0 | 0 | 687 | 687 |
|  | 2035-2039 | 0 | | 0 | 0 | 0 | 0 | 673 | 673 |
| Total | 2015-2019 | 60 | | 0 | 209 | 869 | 8,959 | 11,888 | 21,985 |
|  | 2020-2024 | 55 | | 0 | 225 | 940 | 9,652 | 14,214 | 25,085 |
|  | 2025-2029 | 53 | | 0 | 209 | 1,056 | 8,615 | 17,130 | 27,063 |
|  | 2030-2034 | 50 | | 0 | 178 | 1,130 | 8,658 | 17,663 | 27,680 |
|  | 2035-2039 | 48 | | 0 | 156 | 1,092 | 9,410 | 17,650 | 28,355 |
|  |  |  | |  |  |  |  |  |  |
| Ischemic heart disease in male | | | |  |  |  |  |  |  |
| Sites | Year | Age groups | | | | | | | |
|  |  | 0-14 | | 15-44 | 45-54 | 55-64 | 65-74 | 75+ | Total |
| Oral cavity | 2015-2019 | 0 | | 0 | 0 | 0 | 0 | 701 | 701 |
| & pharynx | 2020-2024 | 0 | | 0 | 0 | 0 | 0 | 871 | 871 |
| C00-C14 | 2025-2029 | 0 | | 0 | 0 | 0 | 0 | 1,092 | 1,092 |
|  | 2030-2034 | 0 | | 0 | 0 | 0 | 0 | 1,083 | 1,083 |
|  | 2035-2039 | 0 | | 0 | 0 | 0 | 0 | 1,031 | 1,031 |
| Esophagus | 2015-2019 | 0 | | 0 | 196 | 674 | 901 | 878 | 2,650 |
| C15 | 2020-2024 | 0 | | 0 | 232 | 647 | 885 | 1,053 | 2,816 |
|  | 2025-2029 | 0 | | 0 | 235 | 721 | 747 | 1,282 | 2,984 |
|  | 2030-2034 | 0 | | 0 | 199 | 840 | 741 | 1,285 | 3,065 |
|  | 2035-2039 | 0 | | 0 | 145 | 826 | 843 | 1,224 | 3,038 |
| Stomach | 2015-2019 | 0 | | 0 | 247 | 1,286 | 4,317 | 5,183 | 11,033 |
| C16 | 2020-2024 | 0 | | 0 | 262 | 1,111 | 4,241 | 5,733 | 11,347 |
|  | 2025-2029 | 0 | | 0 | 255 | 1,083 | 3,373 | 6,606 | 11,317 |
|  | 2030-2034 | 0 | | 0 | 227 | 1,153 | 2,993 | 6,516 | 10,889 |
|  | 2035-2039 | 0 | | 0 | 200 | 1,110 | 3,003 | 6,044 | 10,356 |
| Colon | 2015-2019 | 0 | | 283 | 306 | 792 | 4,522 | 6,309 | 12,212 |
| & rectum | 2020-2024 | 0 | | 251 | 339 | 771 | 4,510 | 7,284 | 13,155 |
| C18-C20 | 2025-2029 | 0 | | 244 | 320 | 830 | 3,731 | 8,534 | 13,658 |
|  | 2030-2034 | 0 | | 218 | 273 | 888 | 3,595 | 8,474 | 13,447 |
|  | 2035-2039 | 0 | | 218 | 252 | 821 | 3,868 | 8,076 | 13,235 |
| Liver | 2015-2019 | 0 | | 0 | 0 | 0 | 1,043 | 2,783 | 3,826 |
| C22 | 2020-2024 | 0 | | 0 | 0 | 0 | 914 | 2,668 | 3,582 |
|  | 2025-2029 | 0 | | 0 | 0 | 0 | 681 | 2,683 | 3,364 |
|  | 2030-2034 | 0 | | 0 | 0 | 0 | 610 | 2,460 | 3,070 |
|  | 2035-2039 | 0 | | 0 | 0 | 0 | 643 | 2,129 | 2,772 |
| Gallbladder | 2015-2019 | 0 | | 0 | 0 | 0 | 444 | 0 | 444 |
| & bile duct | 2020-2024 | 0 | | 0 | 0 | 0 | 444 | 0 | 444 |
| C23-C24 | 2025-2029 | 0 | | 0 | 0 | 0 | 352 | 0 | 352 |
|  | 2030-2034 | 0 | | 0 | 0 | 0 | 321 | 0 | 321 |
|  | 2035-2039 | 0 | | 0 | 0 | 0 | 349 | 0 | 349 |
| Pancreas | 2015-2019 | 0 | | 0 | 0 | 394 | 438 | 0 | 833 |
| C25 | 2020-2024 | 0 | | 0 | 0 | 371 | 436 | 0 | 807 |
|  | 2025-2029 | 0 | | 0 | 0 | 391 | 353 | 0 | 744 |
|  | 2030-2034 | 0 | | 0 | 0 | 408 | 329 | 0 | 737 |
|  | 2035-2039 | 0 | | 0 | 0 | 379 | 349 | 0 | 728 |
| Larynx | 2015-2019 | 0 | | 0 | 0 | 158 | 583 | 216 | 957 |
| C32 | 2020-2024 | 0 | | 0 | 0 | 141 | 557 | 248 | 947 |
|  | 2025-2029 | 0 | | 0 | 0 | 153 | 446 | 288 | 887 |
|  | 2030-2034 | 0 | | 0 | 0 | 168 | 399 | 277 | 843 |
|  | 2035-2039 | 0 | | 0 | 0 | 190 | 440 | 249 | 879 |
| Lung | 2015-2019 | 0 | | 0 | 0 | 744 | 2,786 | 1,487 | 5,016 |
| C33-C34 | 2020-2024 | 0 | | 0 | 0 | 711 | 2,905 | 1,740 | 5,356 |
|  | 2025-2029 | 0 | | 0 | 0 | 738 | 2,438 | 2,105 | 5,281 |
|  | 2030-2034 | 0 | | 0 | 0 | 756 | 2,276 | 2,123 | 5,155 |
|  | 2035-2039 | 0 | | 0 | 0 | 697 | 2,363 | 2,005 | 5,065 |
| Skin | 2015-2019 | 0 | | 0 | 0 | 0 | 142 | 0 | 142 |
| C43-C44 | 2020-2024 | 0 | | 0 | 0 | 0 | 166 | 0 | 166 |
|  | 2025-2029 | 0 | | 0 | 0 | 0 | 155 | 0 | 155 |
|  | 2030-2034 | 0 | | 0 | 0 | 0 | 165 | 0 | 165 |
|  | 2035-2039 | 0 | | 0 | 0 | 0 | 193 | 0 | 193 |
| Prostate | 2015-2019 | 0 | | 0 | 0 | 469 | 2,930 | 4,900 | 8,299 |
| C61 | 2020-2024 | 0 | | 0 | 0 | 477 | 3,526 | 7,021 | 11,024 |
|  | 2025-2029 | 0 | | 0 | 0 | 459 | 3,300 | 9,745 | 13,505 |
|  | 2030-2034 | 0 | | 0 | 0 | 404 | 3,218 | 11,132 | 14,755 |
|  | 2035-2039 | 0 | | 0 | 0 | 358 | 3,118 | 11,709 | 15,185 |
| Urinary | 2015-2019 | 0 | | 0 | 57 | 141 | 844 | 1,283 | 2,324 |
| bladder | 2020-2024 | 0 | | 0 | 51 | 130 | 844 | 1,471 | 2,496 |
| C67 | 2025-2029 | 0 | | 0 | 43 | 126 | 668 | 1,767 | 2,604 |
|  | 2030-2034 | 0 | | 0 | 36 | 117 | 623 | 1,833 | 2,609 |
|  | 2035-2039 | 0 | | 0 | 30 | 100 | 618 | 1,775 | 2,523 |
| Kidney | 2015-2019 | 0 | | 0 | 0 | 496 | 1,034 | 376 | 1,906 |
| & ureter | 2020-2024 | 0 | | 0 | 0 | 552 | 1,209 | 476 | 2,237 |
| C64-C66, | 2025-2029 | 0 | | 0 | 0 | 663 | 1,112 | 619 | 2,394 |
| C68 | 2030-2034 | 0 | | 0 | 0 | 713 | 1,129 | 656 | 2,498 |
|  | 2035-2039 | 0 | | 0 | 0 | 631 | 1,297 | 657 | 2,585 |
| Thyroid | 2015-2019 | 0 | | 0 | 98 | 113 | 0 | 0 | 211 |
| gland | 2020-2024 | 0 | | 0 | 127 | 131 | 0 | 0 | 258 |
| C73 | 2025-2029 | 0 | | 0 | 130 | 164 | 0 | 0 | 294 |
|  | 2030-2034 | 0 | | 0 | 114 | 188 | 0 | 0 | 301 |
|  | 2035-2039 | 0 | | 0 | 117 | 182 | 0 | 0 | 299 |
| Lymphoma | 2015-2019 | 0 | | 0 | 171 | 301 | 283 | 1,171 | 1,926 |
| C81-C85, | 2020-2024 | 0 | | 0 | 203 | 320 | 326 | 1,458 | 2,307 |
| C96 | 2025-2029 | 0 | | 0 | 205 | 348 | 311 | 1,821 | 2,685 |
|  | 2030-2034 | 0 | | 0 | 185 | 372 | 305 | 1,934 | 2,797 |
|  | 2035-2039 | 0 | | 0 | 166 | 357 | 323 | 1,965 | 2,810 |
| Myeloma | 2015-2019 | 0 | | 0 | 0 | 240 | 0 | 0 | 240 |
| C88-C90 | 2020-2024 | 0 | | 0 | 0 | 239 | 0 | 0 | 239 |
|  | 2025-2029 | 0 | | 0 | 0 | 256 | 0 | 0 | 256 |
|  | 2030-2034 | 0 | | 0 | 0 | 269 | 0 | 0 | 269 |
|  | 2035-2039 | 0 | | 0 | 0 | 240 | 0 | 0 | 240 |
| Leukemia | 2015-2019 | 0 | | 0 | 149 | 141 | 0 | 0 | 290 |
| C91-C95 | 2020-2024 | 0 | | 0 | 181 | 145 | 0 | 0 | 326 |
|  | 2025-2029 | 0 | | 0 | 179 | 159 | 0 | 0 | 339 |
|  | 2030-2034 | 0 | | 0 | 162 | 182 | 0 | 0 | 344 |
|  | 2035-2039 | 0 | | 0 | 161 | 174 | 0 | 0 | 335 |
| Total | 2015-2019 | 0 | | 283 | 1,224 | 5,949 | 20,267 | 25,287 | 53,010 |
|  | 2020-2024 | 0 | | 251 | 1,394 | 5,745 | 20,963 | 30,024 | 58,376 |
|  | 2025-2029 | 0 | | 244 | 1,367 | 6,092 | 17,665 | 36,542 | 61,910 |
|  | 2030-2034 | 0 | | 218 | 1,196 | 6,458 | 16,704 | 37,773 | 62,350 |
|  | 2035-2039 | 0 | | 218 | 1,071 | 6,066 | 17,408 | 36,863 | 61,624 |
|  |  |  | |  |  |  |  |  |  |
| Ischemic heart disease in female | | | |  |  |  |  |  |  |
| Sites | Year | Age groups | | | | | | | |
|  |  | 0-14 | | 15-44 | 45-54 | 55-64 | 65-74 | 75+ | Total |
| Oral cavity | 2015-2019 | 0 | | 0 | 0 | 0 | 0 | 0 | 0 |
| & pharynx | 2020-2024 | 0 | | 0 | 0 | 0 | 0 | 0 | 0 |
| C00-C14 | 2025-2029 | 0 | | 0 | 0 | 0 | 0 | 0 | 0 |
|  | 2030-2034 | 0 | | 0 | 0 | 0 | 0 | 0 | 0 |
|  | 2035-2039 | 0 | | 0 | 0 | 0 | 0 | 0 | 0 |
| Esophagus | 2015-2019 | 0 | | 0 | 0 | 0 | 82 | 175 | 256 |
| C15 | 2020-2024 | 0 | | 0 | 0 | 0 | 83 | 205 | 288 |
|  | 2025-2029 | 0 | | 0 | 0 | 0 | 71 | 246 | 317 |
|  | 2030-2034 | 0 | | 0 | 0 | 0 | 76 | 255 | 331 |
|  | 2035-2039 | 0 | | 0 | 0 | 0 | 84 | 257 | 340 |
| Stomach | 2015-2019 | 0 | | 0 | 0 | 0 | 0 | 522 | 522 |
| C16 | 2020-2024 | 0 | | 0 | 0 | 0 | 0 | 569 | 569 |
|  | 2025-2029 | 0 | | 0 | 0 | 0 | 0 | 645 | 645 |
|  | 2030-2034 | 0 | | 0 | 0 | 0 | 0 | 648 | 648 |
|  | 2035-2039 | 0 | | 0 | 0 | 0 | 0 | 619 | 619 |
| Colon | 2015-2019 | 0 | | 0 | 0 | 0 | 552 | 1,914 | 2,466 |
| & rectum | 2020-2024 | 0 | | 0 | 0 | 0 | 554 | 2,201 | 2,755 |
| C18-C20 | 2025-2029 | 0 | | 0 | 0 | 0 | 459 | 2,508 | 2,967 |
|  | 2030-2034 | 0 | | 0 | 0 | 0 | 441 | 2,523 | 2,964 |
|  | 2035-2039 | 0 | | 0 | 0 | 0 | 480 | 2,441 | 2,921 |
| Liver | 2015-2019 | 0 | | 0 | 0 | 0 | 0 | 0 | 0 |
| C22 | 2020-2024 | 0 | | 0 | 0 | 0 | 0 | 0 | 0 |
|  | 2025-2029 | 0 | | 0 | 0 | 0 | 0 | 0 | 0 |
|  | 2030-2034 | 0 | | 0 | 0 | 0 | 0 | 0 | 0 |
|  | 2035-2039 | 0 | | 0 | 0 | 0 | 0 | 0 | 0 |
| Gallbladder | 2015-2019 | 0 | | 0 | 0 | 0 | 209 | 0 | 209 |
| & bile duct | 2020-2024 | 0 | | 0 | 0 | 0 | 200 | 0 | 200 |
| C23-C24 | 2025-2029 | 0 | | 0 | 0 | 0 | 158 | 0 | 158 |
|  | 2030-2034 | 0 | | 0 | 0 | 0 | 137 | 0 | 137 |
|  | 2035-2039 | 0 | | 0 | 0 | 0 | 135 | 0 | 135 |
| Pancreas | 2015-2019 | 0 | | 0 | 0 | 0 | 230 | 0 | 230 |
| C25 | 2020-2024 | 0 | | 0 | 0 | 0 | 245 | 0 | 245 |
|  | 2025-2029 | 0 | | 0 | 0 | 0 | 209 | 0 | 209 |
|  | 2030-2034 | 0 | | 0 | 0 | 0 | 197 | 0 | 197 |
|  | 2035-2039 | 0 | | 0 | 0 | 0 | 217 | 0 | 217 |
| Larynx | 2015-2019 | 0 | | 0 | 0 | 107 | 0 | 0 | 107 |
| C32 | 2020-2024 | 0 | | 0 | 0 | 87 | 0 | 0 | 87 |
|  | 2025-2029 | 0 | | 0 | 0 | 93 | 0 | 0 | 93 |
|  | 2030-2034 | 0 | | 0 | 0 | 113 | 0 | 0 | 113 |
|  | 2035-2039 | 0 | | 0 | 0 | 107 | 0 | 0 | 107 |
| Lung | 2015-2019 | 0 | | 0 | 0 | 0 | 267 | 242 | 510 |
| C33-C34 | 2020-2024 | 0 | | 0 | 0 | 0 | 286 | 300 | 586 |
|  | 2025-2029 | 0 | | 0 | 0 | 0 | 239 | 370 | 609 |
|  | 2030-2034 | 0 | | 0 | 0 | 0 | 223 | 373 | 596 |
|  | 2035-2039 | 0 | | 0 | 0 | 0 | 226 | 358 | 585 |
| Skin | 2015-2019 | 0 | | 0 | 0 | 0 | 0 | 0 | 0 |
| C43-C44 | 2020-2024 | 0 | | 0 | 0 | 0 | 0 | 0 | 0 |
|  | 2025-2029 | 0 | | 0 | 0 | 0 | 0 | 0 | 0 |
|  | 2030-2034 | 0 | | 0 | 0 | 0 | 0 | 0 | 0 |
|  | 2035-2039 | 0 | | 0 | 0 | 0 | 0 | 0 | 0 |
| Breast | 2015-2019 | 0 | | 98 | 114 | 226 | 687 | 515 | 1,640 |
| C50 | 2020-2024 | 0 | | 88 | 127 | 257 | 782 | 712 | 1,966 |
|  | 2025-2029 | 0 | | 79 | 118 | 293 | 732 | 964 | 2,186 |
|  | 2030-2034 | 0 | | 71 | 96 | 301 | 761 | 1,061 | 2,290 |
|  | 2035-2039 | 0 | | 74 | 78 | 268 | 833 | 1,117 | 2,371 |
| Uterus | 2015-2019 | 0 | | 0 | 0 | 209 | 191 | 248 | 647 |
| C53-C55 | 2020-2024 | 0 | | 0 | 0 | 263 | 210 | 310 | 783 |
|  | 2025-2029 | 0 | | 0 | 0 | 336 | 208 | 395 | 939 |
|  | 2030-2034 | 0 | | 0 | 0 | 380 | 243 | 416 | 1,039 |
|  | 2035-2039 | 0 | | 0 | 0 | 386 | 300 | 445 | 1,131 |
| Ovary | 2015-2019 | 0 | | 0 | 0 | 404 | 164 | 211 | 779 |
| C56 | 2020-2024 | 0 | | 0 | 0 | 425 | 164 | 251 | 840 |
|  | 2025-2029 | 0 | | 0 | 0 | 465 | 141 | 310 | 916 |
|  | 2030-2034 | 0 | | 0 | 0 | 462 | 144 | 311 | 917 |
|  | 2035-2039 | 0 | | 0 | 0 | 421 | 155 | 300 | 877 |
| Urinary | 2015-2019 | 0 | | 0 | 0 | 0 | 0 | 0 | 0 |
| bladder | 2020-2024 | 0 | | 0 | 0 | 0 | 0 | 0 | 0 |
| C67 | 2025-2029 | 0 | | 0 | 0 | 0 | 0 | 0 | 0 |
|  | 2030-2034 | 0 | | 0 | 0 | 0 | 0 | 0 | 0 |
|  | 2035-2039 | 0 | | 0 | 0 | 0 | 0 | 0 | 0 |
| Kidney | 2015-2019 | 0 | | 0 | 0 | 0 | 0 | 0 | 0 |
| & ureter | 2020-2024 | 0 | | 0 | 0 | 0 | 0 | 0 | 0 |
| C64-C66, | 2025-2029 | 0 | | 0 | 0 | 0 | 0 | 0 | 0 |
| C68 | 2030-2034 | 0 | | 0 | 0 | 0 | 0 | 0 | 0 |
|  | 2035-2039 | 0 | | 0 | 0 | 0 | 0 | 0 | 0 |
| Thyroid | 2015-2019 | 0 | | 0 | 0 | 0 | 0 | 0 | 0 |
| gland | 2020-2024 | 0 | | 0 | 0 | 0 | 0 | 0 | 0 |
| C73 | 2025-2029 | 0 | | 0 | 0 | 0 | 0 | 0 | 0 |
|  | 2030-2034 | 0 | | 0 | 0 | 0 | 0 | 0 | 0 |
|  | 2035-2039 | 0 | | 0 | 0 | 0 | 0 | 0 | 0 |
| Lymphoma | 2015-2019 | 0 | | 0 | 0 | 109 | 0 | 305 | 414 |
| C81-C85, | 2020-2024 | 0 | | 0 | 0 | 107 | 0 | 360 | 467 |
| C96 | 2025-2029 | 0 | | 0 | 0 | 106 | 0 | 426 | 532 |
|  | 2030-2034 | 0 | | 0 | 0 | 114 | 0 | 437 | 551 |
|  | 2035-2039 | 0 | | 0 | 0 | 116 | 0 | 434 | 550 |
| Myeloma | 2015-2019 | 0 | | 0 | 0 | 0 | 0 | 0 | 0 |
| C88-C90 | 2020-2024 | 0 | | 0 | 0 | 0 | 0 | 0 | 0 |
|  | 2025-2029 | 0 | | 0 | 0 | 0 | 0 | 0 | 0 |
|  | 2030-2034 | 0 | | 0 | 0 | 0 | 0 | 0 | 0 |
|  | 2035-2039 | 0 | | 0 | 0 | 0 | 0 | 0 | 0 |
| Leukemia | 2015-2019 | 0 | | 0 | 0 | 0 | 0 | 0 | 0 |
| C91-C95 | 2020-2024 | 0 | | 0 | 0 | 0 | 0 | 0 | 0 |
|  | 2025-2029 | 0 | | 0 | 0 | 0 | 0 | 0 | 0 |
|  | 2030-2034 | 0 | | 0 | 0 | 0 | 0 | 0 | 0 |
|  | 2035-2039 | 0 | | 0 | 0 | 0 | 0 | 0 | 0 |
| Total | 2015-2019 | 0 | | 98 | 114 | 1,055 | 2,381 | 4,132 | 7,781 |
|  | 2020-2024 | 0 | | 88 | 127 | 1,139 | 2,524 | 4,908 | 8,786 |
|  | 2025-2029 | 0 | | 79 | 118 | 1,294 | 2,218 | 5,863 | 9,571 |
|  | 2030-2034 | 0 | | 71 | 96 | 1,370 | 2,221 | 6,024 | 9,782 |
|  | 2035-2039 | 0 | | 74 | 78 | 1,298 | 2,430 | 5,971 | 9,851 |
|  |  |  | |  |  |  |  |  |  |
| Aortic stenosis in male | | |  |  |  |  |  |  |  |
| Sites | Year | Age groups | | | | | | | |
|  |  | 0-14 | | 15-44 | 45-54 | 55-64 | 65-74 | 75+ | Total |
| Oral cavity | 2015-2019 | 0 | | 0 | 0 | 0 | 0 | 0 | 0 |
| & pharynx | 2020-2024 | 0 | | 0 | 0 | 0 | 0 | 0 | 0 |
| C00-C14 | 2025-2029 | 0 | | 0 | 0 | 0 | 0 | 0 | 0 |
|  | 2030-2034 | 0 | | 0 | 0 | 0 | 0 | 0 | 0 |
|  | 2035-2039 | 0 | | 0 | 0 | 0 | 0 | 0 | 0 |
| Esophagus | 2015-2019 | 0 | | 0 | 0 | 0 | 0 | 0 | 0 |
| C15 | 2020-2024 | 0 | | 0 | 0 | 0 | 0 | 0 | 0 |
|  | 2025-2029 | 0 | | 0 | 0 | 0 | 0 | 0 | 0 |
|  | 2030-2034 | 0 | | 0 | 0 | 0 | 0 | 0 | 0 |
|  | 2035-2039 | 0 | | 0 | 0 | 0 | 0 | 0 | 0 |
| Stomach | 2015-2019 | 0 | | 0 | 0 | 198 | 375 | 972 | 1,545 |
| C16 | 2020-2024 | 0 | | 0 | 0 | 171 | 369 | 1,075 | 1,615 |
|  | 2025-2029 | 0 | | 0 | 0 | 167 | 293 | 1,239 | 1,699 |
|  | 2030-2034 | 0 | | 0 | 0 | 177 | 260 | 1,222 | 1,659 |
|  | 2035-2039 | 0 | | 0 | 0 | 171 | 261 | 1,133 | 1,565 |
| Colon | 2015-2019 | 0 | | 0 | 0 | 0 | 0 | 2,103 | 2,103 |
| & rectum | 2020-2024 | 0 | | 0 | 0 | 0 | 0 | 2,428 | 2,428 |
| C18-C20 | 2025-2029 | 0 | | 0 | 0 | 0 | 0 | 2,845 | 2,845 |
|  | 2030-2034 | 0 | | 0 | 0 | 0 | 0 | 2,825 | 2,825 |
|  | 2035-2039 | 0 | | 0 | 0 | 0 | 0 | 2,692 | 2,692 |
| Liver | 2015-2019 | 0 | | 0 | 0 | 0 | 1,043 | 0 | 1,043 |
| C22 | 2020-2024 | 0 | | 0 | 0 | 0 | 914 | 0 | 914 |
|  | 2025-2029 | 0 | | 0 | 0 | 0 | 681 | 0 | 681 |
|  | 2030-2034 | 0 | | 0 | 0 | 0 | 610 | 0 | 610 |
|  | 2035-2039 | 0 | | 0 | 0 | 0 | 643 | 0 | 643 |
| Gallbladder | 2015-2019 | 0 | | 0 | 0 | 0 | 444 | 415 | 859 |
| & bile duct | 2020-2024 | 0 | | 0 | 0 | 0 | 444 | 469 | 913 |
| C23-C24 | 2025-2029 | 0 | | 0 | 0 | 0 | 352 | 550 | 902 |
|  | 2030-2034 | 0 | | 0 | 0 | 0 | 321 | 550 | 871 |
|  | 2035-2039 | 0 | | 0 | 0 | 0 | 349 | 518 | 867 |
| Pancreas | 2015-2019 | 0 | | 0 | 0 | 0 | 0 | 0 | 0 |
| C25 | 2020-2024 | 0 | | 0 | 0 | 0 | 0 | 0 | 0 |
|  | 2025-2029 | 0 | | 0 | 0 | 0 | 0 | 0 | 0 |
|  | 2030-2034 | 0 | | 0 | 0 | 0 | 0 | 0 | 0 |
|  | 2035-2039 | 0 | | 0 | 0 | 0 | 0 | 0 | 0 |
| Larynx | 2015-2019 | 0 | | 0 | 0 | 0 | 117 | 0 | 117 |
| C32 | 2020-2024 | 0 | | 0 | 0 | 0 | 111 | 0 | 111 |
|  | 2025-2029 | 0 | | 0 | 0 | 0 | 89 | 0 | 89 |
|  | 2030-2034 | 0 | | 0 | 0 | 0 | 80 | 0 | 80 |
|  | 2035-2039 | 0 | | 0 | 0 | 0 | 88 | 0 | 88 |
| Lung | 2015-2019 | 0 | | 0 | 0 | 0 | 440 | 496 | 935 |
| C33-C34 | 2020-2024 | 0 | | 0 | 0 | 0 | 459 | 580 | 1,039 |
|  | 2025-2029 | 0 | | 0 | 0 | 0 | 385 | 702 | 1,087 |
|  | 2030-2034 | 0 | | 0 | 0 | 0 | 359 | 708 | 1,067 |
|  | 2035-2039 | 0 | | 0 | 0 | 0 | 373 | 668 | 1,042 |
| Skin | 2015-2019 | 0 | | 0 | 0 | 0 | 142 | 0 | 142 |
| C43-C44 | 2020-2024 | 0 | | 0 | 0 | 0 | 166 | 0 | 166 |
|  | 2025-2029 | 0 | | 0 | 0 | 0 | 155 | 0 | 155 |
|  | 2030-2034 | 0 | | 0 | 0 | 0 | 165 | 0 | 165 |
|  | 2035-2039 | 0 | | 0 | 0 | 0 | 193 | 0 | 193 |
| Prostate | 2015-2019 | 0 | | 0 | 0 | 0 | 488 | 1,633 | 2,122 |
| C61 | 2020-2024 | 0 | | 0 | 0 | 0 | 588 | 2,340 | 2,928 |
|  | 2025-2029 | 0 | | 0 | 0 | 0 | 550 | 3,248 | 3,798 |
|  | 2030-2034 | 0 | | 0 | 0 | 0 | 536 | 3,711 | 4,247 |
|  | 2035-2039 | 0 | | 0 | 0 | 0 | 520 | 3,903 | 4,423 |
| Urinary | 2015-2019 | 0 | | 0 | 0 | 0 | 316 | 962 | 1,279 |
| bladder | 2020-2024 | 0 | | 0 | 0 | 0 | 316 | 1,104 | 1,420 |
| C67 | 2025-2029 | 0 | | 0 | 0 | 0 | 250 | 1,325 | 1,576 |
|  | 2030-2034 | 0 | | 0 | 0 | 0 | 234 | 1,375 | 1,609 |
|  | 2035-2039 | 0 | | 0 | 0 | 0 | 232 | 1,331 | 1,563 |
| Kidney | 2015-2019 | 0 | | 0 | 0 | 0 | 0 | 376 | 376 |
| & ureter | 2020-2024 | 0 | | 0 | 0 | 0 | 0 | 476 | 476 |
| C64-C66, | 2025-2029 | 0 | | 0 | 0 | 0 | 0 | 619 | 619 |
| C68 | 2030-2034 | 0 | | 0 | 0 | 0 | 0 | 656 | 656 |
|  | 2035-2039 | 0 | | 0 | 0 | 0 | 0 | 657 | 657 |
| Thyroid | 2015-2019 | 0 | | 0 | 0 | 0 | 0 | 0 | 0 |
| gland | 2020-2024 | 0 | | 0 | 0 | 0 | 0 | 0 | 0 |
| C73 | 2025-2029 | 0 | | 0 | 0 | 0 | 0 | 0 | 0 |
|  | 2030-2034 | 0 | | 0 | 0 | 0 | 0 | 0 | 0 |
|  | 2035-2039 | 0 | | 0 | 0 | 0 | 0 | 0 | 0 |
| Lymphoma | 2015-2019 | 0 | | 0 | 0 | 0 | 0 | 0 | 0 |
| C81-C85, | 2020-2024 | 0 | | 0 | 0 | 0 | 0 | 0 | 0 |
| C96 | 2025-2029 | 0 | | 0 | 0 | 0 | 0 | 0 | 0 |
|  | 2030-2034 | 0 | | 0 | 0 | 0 | 0 | 0 | 0 |
|  | 2035-2039 | 0 | | 0 | 0 | 0 | 0 | 0 | 0 |
| Myeloma | 2015-2019 | 0 | | 0 | 0 | 0 | 0 | 0 | 0 |
| C88-C90 | 2020-2024 | 0 | | 0 | 0 | 0 | 0 | 0 | 0 |
|  | 2025-2029 | 0 | | 0 | 0 | 0 | 0 | 0 | 0 |
|  | 2030-2034 | 0 | | 0 | 0 | 0 | 0 | 0 | 0 |
|  | 2035-2039 | 0 | | 0 | 0 | 0 | 0 | 0 | 0 |
| Leukemia | 2015-2019 | 0 | | 0 | 0 | 0 | 675 | 470 | 1,145 |
| C91-C95 | 2020-2024 | 0 | | 0 | 0 | 0 | 688 | 539 | 1,227 |
|  | 2025-2029 | 0 | | 0 | 0 | 0 | 578 | 639 | 1,217 |
|  | 2030-2034 | 0 | | 0 | 0 | 0 | 572 | 630 | 1,202 |
|  | 2035-2039 | 0 | | 0 | 0 | 0 | 618 | 603 | 1,221 |
| Total | 2015-2019 | 0 | | 0 | 0 | 198 | 4,040 | 7,427 | 11,665 |
|  | 2020-2024 | 0 | | 0 | 0 | 171 | 4,055 | 9,011 | 13,237 |
|  | 2025-2029 | 0 | | 0 | 0 | 167 | 3,333 | 11,166 | 14,666 |
|  | 2030-2034 | 0 | | 0 | 0 | 177 | 3,137 | 11,675 | 14,990 |
|  | 2035-2039 | 0 | | 0 | 0 | 171 | 3,277 | 11,505 | 14,953 |
|  |  |  | |  |  |  |  |  |  |
| Aortic stenosis in female | | |  |  |  |  |  |  |  |
| Sites | Year | Age groups | | | | | | | |
|  |  | 0-14 | | 15-44 | 45-54 | 55-64 | 65-74 | 75+ | Total |
| Oral cavity | 2015-2019 | 0 | | 0 | 0 | 0 | 0 | 0 | 0 |
| & pharynx | 2020-2024 | 0 | | 0 | 0 | 0 | 0 | 0 | 0 |
| C00-C14 | 2025-2029 | 0 | | 0 | 0 | 0 | 0 | 0 | 0 |
|  | 2030-2034 | 0 | | 0 | 0 | 0 | 0 | 0 | 0 |
|  | 2035-2039 | 0 | | 0 | 0 | 0 | 0 | 0 | 0 |
| Esophagus | 2015-2019 | 0 | | 0 | 0 | 0 | 0 | 175 | 175 |
| C15 | 2020-2024 | 0 | | 0 | 0 | 0 | 0 | 205 | 205 |
|  | 2025-2029 | 0 | | 0 | 0 | 0 | 0 | 246 | 246 |
|  | 2030-2034 | 0 | | 0 | 0 | 0 | 0 | 255 | 255 |
|  | 2035-2039 | 0 | | 0 | 0 | 0 | 0 | 257 | 257 |
| Stomach | 2015-2019 | 0 | | 0 | 0 | 0 | 296 | 522 | 818 |
| C16 | 2020-2024 | 0 | | 0 | 0 | 0 | 292 | 569 | 862 |
|  | 2025-2029 | 0 | | 0 | 0 | 0 | 231 | 645 | 876 |
|  | 2030-2034 | 0 | | 0 | 0 | 0 | 208 | 648 | 856 |
|  | 2035-2039 | 0 | | 0 | 0 | 0 | 207 | 619 | 825 |
| Colon | 2015-2019 | 0 | | 0 | 0 | 0 | 0 | 0 | 0 |
| & rectum | 2020-2024 | 0 | | 0 | 0 | 0 | 0 | 0 | 0 |
| C18-C20 | 2025-2029 | 0 | | 0 | 0 | 0 | 0 | 0 | 0 |
|  | 2030-2034 | 0 | | 0 | 0 | 0 | 0 | 0 | 0 |
|  | 2035-2039 | 0 | | 0 | 0 | 0 | 0 | 0 | 0 |
| Liver | 2015-2019 | 0 | | 0 | 0 | 0 | 0 | 1,823 | 1,823 |
| C22 | 2020-2024 | 0 | | 0 | 0 | 0 | 0 | 1,725 | 1,725 |
|  | 2025-2029 | 0 | | 0 | 0 | 0 | 0 | 1,678 | 1,678 |
|  | 2030-2034 | 0 | | 0 | 0 | 0 | 0 | 1,534 | 1,534 |
|  | 2035-2039 | 0 | | 0 | 0 | 0 | 0 | 1,371 | 1,371 |
| Gallbladder | 2015-2019 | 0 | | 0 | 0 | 0 | 0 | 0 | 0 |
| & bile duct | 2020-2024 | 0 | | 0 | 0 | 0 | 0 | 0 | 0 |
| C23-C24 | 2025-2029 | 0 | | 0 | 0 | 0 | 0 | 0 | 0 |
|  | 2030-2034 | 0 | | 0 | 0 | 0 | 0 | 0 | 0 |
|  | 2035-2039 | 0 | | 0 | 0 | 0 | 0 | 0 | 0 |
| Pancreas | 2015-2019 | 0 | | 0 | 0 | 0 | 0 | 0 | 0 |
| C25 | 2020-2024 | 0 | | 0 | 0 | 0 | 0 | 0 | 0 |
|  | 2025-2029 | 0 | | 0 | 0 | 0 | 0 | 0 | 0 |
|  | 2030-2034 | 0 | | 0 | 0 | 0 | 0 | 0 | 0 |
|  | 2035-2039 | 0 | | 0 | 0 | 0 | 0 | 0 | 0 |
| Larynx | 2015-2019 | 0 | | 0 | 0 | 0 | 0 | 0 | 0 |
| C32 | 2020-2024 | 0 | | 0 | 0 | 0 | 0 | 0 | 0 |
|  | 2025-2029 | 0 | | 0 | 0 | 0 | 0 | 0 | 0 |
|  | 2030-2034 | 0 | | 0 | 0 | 0 | 0 | 0 | 0 |
|  | 2035-2039 | 0 | | 0 | 0 | 0 | 0 | 0 | 0 |
| Lung | 2015-2019 | 0 | | 0 | 0 | 0 | 267 | 970 | 1,237 |
| C33-C34 | 2020-2024 | 0 | | 0 | 0 | 0 | 286 | 1,199 | 1,485 |
|  | 2025-2029 | 0 | | 0 | 0 | 0 | 239 | 1,479 | 1,718 |
|  | 2030-2034 | 0 | | 0 | 0 | 0 | 223 | 1,493 | 1,716 |
|  | 2035-2039 | 0 | | 0 | 0 | 0 | 226 | 1,434 | 1,660 |
| Skin | 2015-2019 | 0 | | 0 | 0 | 0 | 128 | 1,293 | 1,421 |
| C43-C44 | 2020-2024 | 0 | | 0 | 0 | 0 | 148 | 1,672 | 1,820 |
|  | 2025-2029 | 0 | | 0 | 0 | 0 | 137 | 2,021 | 2,158 |
|  | 2030-2034 | 0 | | 0 | 0 | 0 | 144 | 2,197 | 2,341 |
|  | 2035-2039 | 0 | | 0 | 0 | 0 | 160 | 2,356 | 2,516 |
| Breast | 2015-2019 | 0 | | 0 | 0 | 0 | 1,145 | 2,059 | 3,204 |
| C50 | 2020-2024 | 0 | | 0 | 0 | 0 | 1,303 | 2,847 | 4,150 |
|  | 2025-2029 | 0 | | 0 | 0 | 0 | 1,219 | 3,857 | 5,076 |
|  | 2030-2034 | 0 | | 0 | 0 | 0 | 1,268 | 4,244 | 5,512 |
|  | 2035-2039 | 0 | | 0 | 0 | 0 | 1,389 | 4,469 | 5,858 |
| Uterus | 2015-2019 | 0 | | 0 | 0 | 0 | 191 | 0 | 191 |
| C53-C55 | 2020-2024 | 0 | | 0 | 0 | 0 | 210 | 0 | 210 |
|  | 2025-2029 | 0 | | 0 | 0 | 0 | 208 | 0 | 208 |
|  | 2030-2034 | 0 | | 0 | 0 | 0 | 243 | 0 | 243 |
|  | 2035-2039 | 0 | | 0 | 0 | 0 | 300 | 0 | 300 |
| Ovary | 2015-2019 | 0 | | 0 | 0 | 0 | 0 | 0 | 0 |
| C56 | 2020-2024 | 0 | | 0 | 0 | 0 | 0 | 0 | 0 |
|  | 2025-2029 | 0 | | 0 | 0 | 0 | 0 | 0 | 0 |
|  | 2030-2034 | 0 | | 0 | 0 | 0 | 0 | 0 | 0 |
|  | 2035-2039 | 0 | | 0 | 0 | 0 | 0 | 0 | 0 |
| Urinary | 2015-2019 | 0 | | 0 | 0 | 0 | 95 | 0 | 95 |
| bladder | 2020-2024 | 0 | | 0 | 0 | 0 | 103 | 0 | 103 |
| C67 | 2025-2029 | 0 | | 0 | 0 | 0 | 85 | 0 | 85 |
|  | 2030-2034 | 0 | | 0 | 0 | 0 | 82 | 0 | 82 |
|  | 2035-2039 | 0 | | 0 | 0 | 0 | 89 | 0 | 89 |
| Kidney | 2015-2019 | 0 | | 0 | 0 | 123 | 122 | 334 | 579 |
| & ureter | 2020-2024 | 0 | | 0 | 0 | 125 | 140 | 400 | 664 |
| C64-C66, | 2025-2029 | 0 | | 0 | 0 | 139 | 120 | 488 | 746 |
| C68 | 2030-2034 | 0 | | 0 | 0 | 147 | 112 | 508 | 767 |
|  | 2035-2039 | 0 | | 0 | 0 | 130 | 120 | 509 | 759 |
| Thyroid | 2015-2019 | 0 | | 0 | 0 | 0 | 0 | 0 | 0 |
| gland | 2020-2024 | 0 | | 0 | 0 | 0 | 0 | 0 | 0 |
| C73 | 2025-2029 | 0 | | 0 | 0 | 0 | 0 | 0 | 0 |
|  | 2030-2034 | 0 | | 0 | 0 | 0 | 0 | 0 | 0 |
|  | 2035-2039 | 0 | | 0 | 0 | 0 | 0 | 0 | 0 |
| Lymphoma | 2015-2019 | 0 | | 0 | 0 | 109 | 231 | 610 | 950 |
| C81-C85, | 2020-2024 | 0 | | 0 | 0 | 107 | 245 | 720 | 1,072 |
| C96 | 2025-2029 | 0 | | 0 | 0 | 106 | 220 | 851 | 1,177 |
|  | 2030-2034 | 0 | | 0 | 0 | 114 | 205 | 874 | 1,193 |
|  | 2035-2039 | 0 | | 0 | 0 | 116 | 200 | 868 | 1,184 |
| Myeloma | 2015-2019 | 0 | | 0 | 0 | 0 | 0 | 0 | 0 |
| C88-C90 | 2020-2024 | 0 | | 0 | 0 | 0 | 0 | 0 | 0 |
|  | 2025-2029 | 0 | | 0 | 0 | 0 | 0 | 0 | 0 |
|  | 2030-2034 | 0 | | 0 | 0 | 0 | 0 | 0 | 0 |
|  | 2035-2039 | 0 | | 0 | 0 | 0 | 0 | 0 | 0 |
| Leukemia | 2015-2019 | 0 | | 0 | 0 | 0 | 0 | 0 | 0 |
| C91-C95 | 2020-2024 | 0 | | 0 | 0 | 0 | 0 | 0 | 0 |
|  | 2025-2029 | 0 | | 0 | 0 | 0 | 0 | 0 | 0 |
|  | 2030-2034 | 0 | | 0 | 0 | 0 | 0 | 0 | 0 |
|  | 2035-2039 | 0 | | 0 | 0 | 0 | 0 | 0 | 0 |
| Total | 2015-2019 | 0 | | 0 | 0 | 231 | 2,474 | 7,786 | 10,492 |
|  | 2020-2024 | 0 | | 0 | 0 | 232 | 2,728 | 9,337 | 12,297 |
|  | 2025-2029 | 0 | | 0 | 0 | 245 | 2,460 | 11,264 | 13,969 |
|  | 2030-2034 | 0 | | 0 | 0 | 261 | 2,485 | 11,752 | 14,498 |
|  | 2035-2039 | 0 | | 0 | 0 | 246 | 2,690 | 11,882 | 14,819 |
|  |  |  | |  |  |  |  |  |  |
| Venous thromboembolism in male | | | |  |  |  |  |  |  |
| Sites | Year | Age groups | | | | | | | |
|  |  | 0-14 | | 15-44 | 45-54 | 55-64 | 65-74 | 75+ | Total |
| Oral cavity | 2015-2019 | 0 | | 0 | 0 | 195 | 0 | 701 | 896 |
| & pharynx | 2020-2024 | 0 | | 0 | 0 | 195 | 0 | 871 | 1,065 |
| C00-C14 | 2025-2029 | 0 | | 0 | 0 | 226 | 0 | 1,092 | 1,318 |
|  | 2030-2034 | 0 | | 0 | 0 | 257 | 0 | 1,083 | 1,340 |
|  | 2035-2039 | 0 | | 0 | 0 | 236 | 0 | 1,031 | 1,266 |
| Esophagus | 2015-2019 | 0 | | 0 | 98 | 337 | 258 | 439 | 1,132 |
| C15 | 2020-2024 | 0 | | 0 | 116 | 323 | 253 | 526 | 1,218 |
|  | 2025-2029 | 0 | | 0 | 117 | 360 | 213 | 641 | 1,332 |
|  | 2030-2034 | 0 | | 0 | 100 | 420 | 212 | 642 | 1,374 |
|  | 2035-2039 | 0 | | 0 | 73 | 413 | 241 | 612 | 1,338 |
| Stomach | 2015-2019 | 0 | | 0 | 0 | 396 | 563 | 1,296 | 2,255 |
| C16 | 2020-2024 | 0 | | 0 | 0 | 342 | 553 | 1,433 | 2,328 |
|  | 2025-2029 | 0 | | 0 | 0 | 333 | 440 | 1,651 | 2,425 |
|  | 2030-2034 | 0 | | 0 | 0 | 355 | 390 | 1,629 | 2,374 |
|  | 2035-2039 | 0 | | 0 | 0 | 341 | 392 | 1,511 | 2,244 |
| Colon | 2015-2019 | 0 | | 0 | 612 | 158 | 969 | 3,154 | 4,894 |
| & rectum | 2020-2024 | 0 | | 0 | 678 | 154 | 967 | 3,642 | 5,441 |
| C18-C20 | 2025-2029 | 0 | | 0 | 641 | 166 | 799 | 4,267 | 5,873 |
|  | 2030-2034 | 0 | | 0 | 545 | 178 | 770 | 4,237 | 5,730 |
|  | 2035-2039 | 0 | | 0 | 504 | 164 | 829 | 4,038 | 5,536 |
| Liver | 2015-2019 | 0 | | 0 | 0 | 0 | 0 | 0 | 0 |
| C22 | 2020-2024 | 0 | | 0 | 0 | 0 | 0 | 0 | 0 |
|  | 2025-2029 | 0 | | 0 | 0 | 0 | 0 | 0 | 0 |
|  | 2030-2034 | 0 | | 0 | 0 | 0 | 0 | 0 | 0 |
|  | 2035-2039 | 0 | | 0 | 0 | 0 | 0 | 0 | 0 |
| Gallbladder | 2015-2019 | 0 | | 0 | 0 | 0 | 0 | 415 | 415 |
| & bile duct | 2020-2024 | 0 | | 0 | 0 | 0 | 0 | 469 | 469 |
| C23-C24 | 2025-2029 | 0 | | 0 | 0 | 0 | 0 | 550 | 550 |
|  | 2030-2034 | 0 | | 0 | 0 | 0 | 0 | 550 | 550 |
|  | 2035-2039 | 0 | | 0 | 0 | 0 | 0 | 518 | 518 |
| Pancreas | 2015-2019 | 0 | | 0 | 305 | 0 | 438 | 0 | 743 |
| C25 | 2020-2024 | 0 | | 0 | 328 | 0 | 436 | 0 | 764 |
|  | 2025-2029 | 0 | | 0 | 310 | 0 | 353 | 0 | 663 |
|  | 2030-2034 | 0 | | 0 | 263 | 0 | 329 | 0 | 592 |
|  | 2035-2039 | 0 | | 0 | 190 | 0 | 349 | 0 | 539 |
| Larynx | 2015-2019 | 0 | | 0 | 0 | 0 | 233 | 0 | 233 |
| C32 | 2020-2024 | 0 | | 0 | 0 | 0 | 223 | 0 | 223 |
|  | 2025-2029 | 0 | | 0 | 0 | 0 | 178 | 0 | 178 |
|  | 2030-2034 | 0 | | 0 | 0 | 0 | 159 | 0 | 159 |
|  | 2035-2039 | 0 | | 0 | 0 | 0 | 176 | 0 | 176 |
| Lung | 2015-2019 | 0 | | 155 | 0 | 149 | 880 | 496 | 1,679 |
| C33-C34 | 2020-2024 | 0 | | 161 | 0 | 142 | 917 | 580 | 1,801 |
|  | 2025-2029 | 0 | | 159 | 0 | 148 | 770 | 702 | 1,778 |
|  | 2030-2034 | 0 | | 156 | 0 | 151 | 719 | 708 | 1,734 |
|  | 2035-2039 | 0 | | 159 | 0 | 139 | 746 | 668 | 1,713 |
| Skin | 2015-2019 | 0 | | 0 | 0 | 0 | 0 | 0 | 0 |
| C43-C44 | 2020-2024 | 0 | | 0 | 0 | 0 | 0 | 0 | 0 |
|  | 2025-2029 | 0 | | 0 | 0 | 0 | 0 | 0 | 0 |
|  | 2030-2034 | 0 | | 0 | 0 | 0 | 0 | 0 | 0 |
|  | 2035-2039 | 0 | | 0 | 0 | 0 | 0 | 0 | 0 |
| Prostate | 2015-2019 | 0 | | 0 | 0 | 0 | 1,221 | 2,042 | 3,262 |
| C61 | 2020-2024 | 0 | | 0 | 0 | 0 | 1,469 | 2,925 | 4,394 |
|  | 2025-2029 | 0 | | 0 | 0 | 0 | 1,375 | 4,061 | 5,436 |
|  | 2030-2034 | 0 | | 0 | 0 | 0 | 1,341 | 4,639 | 5,980 |
|  | 2035-2039 | 0 | | 0 | 0 | 0 | 1,299 | 4,879 | 6,178 |
| Urinary | 2015-2019 | 0 | | 0 | 57 | 0 | 211 | 160 | 428 |
| bladder | 2020-2024 | 0 | | 0 | 51 | 0 | 211 | 184 | 446 |
| C67 | 2025-2029 | 0 | | 0 | 43 | 0 | 167 | 221 | 431 |
|  | 2030-2034 | 0 | | 0 | 36 | 0 | 156 | 229 | 421 |
|  | 2035-2039 | 0 | | 0 | 30 | 0 | 155 | 222 | 406 |
| Kidney | 2015-2019 | 0 | | 0 | 0 | 0 | 861 | 0 | 861 |
| & ureter | 2020-2024 | 0 | | 0 | 0 | 0 | 1,007 | 0 | 1,007 |
| C64-C66, | 2025-2029 | 0 | | 0 | 0 | 0 | 927 | 0 | 927 |
| C68 | 2030-2034 | 0 | | 0 | 0 | 0 | 941 | 0 | 941 |
|  | 2035-2039 | 0 | | 0 | 0 | 0 | 1,081 | 0 | 1,081 |
| Thyroid | 2015-2019 | 0 | | 0 | 0 | 226 | 0 | 0 | 226 |
| gland | 2020-2024 | 0 | | 0 | 0 | 262 | 0 | 0 | 262 |
| C73 | 2025-2029 | 0 | | 0 | 0 | 327 | 0 | 0 | 327 |
|  | 2030-2034 | 0 | | 0 | 0 | 375 | 0 | 0 | 375 |
|  | 2035-2039 | 0 | | 0 | 0 | 364 | 0 | 0 | 364 |
| Lymphoma | 2015-2019 | 39 | | 173 | 0 | 150 | 283 | 390 | 1,036 |
| C81-C85, | 2020-2024 | 37 | | 182 | 0 | 160 | 326 | 486 | 1,191 |
| C96 | 2025-2029 | 35 | | 184 | 0 | 174 | 311 | 607 | 1,311 |
|  | 2030-2034 | 33 | | 180 | 0 | 186 | 305 | 645 | 1,349 |
|  | 2035-2039 | 31 | | 184 | 0 | 178 | 323 | 655 | 1,371 |
| Myeloma | 2015-2019 | 0 | | 0 | 0 | 0 | 0 | 0 | 0 |
| C88-C90 | 2020-2024 | 0 | | 0 | 0 | 0 | 0 | 0 | 0 |
|  | 2025-2029 | 0 | | 0 | 0 | 0 | 0 | 0 | 0 |
|  | 2030-2034 | 0 | | 0 | 0 | 0 | 0 | 0 | 0 |
|  | 2035-2039 | 0 | | 0 | 0 | 0 | 0 | 0 | 0 |
| Leukemia | 2015-2019 | 0 | | 0 | 0 | 0 | 0 | 0 | 0 |
| C91-C95 | 2020-2024 | 0 | | 0 | 0 | 0 | 0 | 0 | 0 |
|  | 2025-2029 | 0 | | 0 | 0 | 0 | 0 | 0 | 0 |
|  | 2030-2034 | 0 | | 0 | 0 | 0 | 0 | 0 | 0 |
|  | 2035-2039 | 0 | | 0 | 0 | 0 | 0 | 0 | 0 |
| Total | 2015-2019 | 39 | | 328 | 1,072 | 1,611 | 5,918 | 9,093 | 18,060 |
|  | 2020-2024 | 37 | | 344 | 1,172 | 1,578 | 6,362 | 11,117 | 20,610 |
|  | 2025-2029 | 35 | | 343 | 1,111 | 1,734 | 5,534 | 13,792 | 22,548 |
|  | 2030-2034 | 33 | | 336 | 943 | 1,922 | 5,323 | 14,361 | 22,918 |
|  | 2035-2039 | 31 | | 344 | 797 | 1,836 | 5,590 | 14,133 | 22,730 |
|  |  |  | |  |  |  |  |  |  |
| Venous thromboembolism in female | | | |  |  |  |  |  |  |
| Sites | Year | Age groups | | | | | | | |
|  |  | 0-14 | | 15-44 | 45-54 | 55-64 | 65-74 | 75+ | Total |
| Oral cavity | 2015-2019 | 0 | | 0 | 0 | 0 | 0 | 0 | 0 |
| & pharynx | 2020-2024 | 0 | | 0 | 0 | 0 | 0 | 0 | 0 |
| C00-C14 | 2025-2029 | 0 | | 0 | 0 | 0 | 0 | 0 | 0 |
|  | 2030-2034 | 0 | | 0 | 0 | 0 | 0 | 0 | 0 |
|  | 2035-2039 | 0 | | 0 | 0 | 0 | 0 | 0 | 0 |
| Esophagus | 2015-2019 | 0 | | 0 | 0 | 0 | 163 | 0 | 163 |
| C15 | 2020-2024 | 0 | | 0 | 0 | 0 | 165 | 0 | 165 |
|  | 2025-2029 | 0 | | 0 | 0 | 0 | 143 | 0 | 143 |
|  | 2030-2034 | 0 | | 0 | 0 | 0 | 152 | 0 | 152 |
|  | 2035-2039 | 0 | | 0 | 0 | 0 | 168 | 0 | 168 |
| Stomach | 2015-2019 | 0 | | 141 | 0 | 169 | 0 | 0 | 309 |
| C16 | 2020-2024 | 0 | | 130 | 0 | 149 | 0 | 0 | 279 |
|  | 2025-2029 | 0 | | 128 | 0 | 146 | 0 | 0 | 273 |
|  | 2030-2034 | 0 | | 127 | 0 | 147 | 0 | 0 | 274 |
|  | 2035-2039 | 0 | | 124 | 0 | 137 | 0 | 0 | 261 |
| Colon | 2015-2019 | 0 | | 0 | 0 | 972 | 1,933 | 2,552 | 5,457 |
| & rectum | 2020-2024 | 0 | | 0 | 0 | 959 | 1,939 | 2,935 | 5,832 |
| C18-C20 | 2025-2029 | 0 | | 0 | 0 | 1,060 | 1,608 | 3,343 | 6,011 |
|  | 2030-2034 | 0 | | 0 | 0 | 1,098 | 1,543 | 3,364 | 6,005 |
|  | 2035-2039 | 0 | | 0 | 0 | 993 | 1,678 | 3,255 | 5,927 |
| Liver | 2015-2019 | 0 | | 0 | 0 | 0 | 0 | 0 | 0 |
| C22 | 2020-2024 | 0 | | 0 | 0 | 0 | 0 | 0 | 0 |
|  | 2025-2029 | 0 | | 0 | 0 | 0 | 0 | 0 | 0 |
|  | 2030-2034 | 0 | | 0 | 0 | 0 | 0 | 0 | 0 |
|  | 2035-2039 | 0 | | 0 | 0 | 0 | 0 | 0 | 0 |
| Gallbladder | 2015-2019 | 0 | | 0 | 0 | 0 | 0 | 0 | 0 |
| & bile duct | 2020-2024 | 0 | | 0 | 0 | 0 | 0 | 0 | 0 |
| C23-C24 | 2025-2029 | 0 | | 0 | 0 | 0 | 0 | 0 | 0 |
|  | 2030-2034 | 0 | | 0 | 0 | 0 | 0 | 0 | 0 |
|  | 2035-2039 | 0 | | 0 | 0 | 0 | 0 | 0 | 0 |
| Pancreas | 2015-2019 | 0 | | 0 | 0 | 0 | 0 | 0 | 0 |
| C25 | 2020-2024 | 0 | | 0 | 0 | 0 | 0 | 0 | 0 |
|  | 2025-2029 | 0 | | 0 | 0 | 0 | 0 | 0 | 0 |
|  | 2030-2034 | 0 | | 0 | 0 | 0 | 0 | 0 | 0 |
|  | 2035-2039 | 0 | | 0 | 0 | 0 | 0 | 0 | 0 |
| Larynx | 2015-2019 | 0 | | 0 | 0 | 0 | 0 | 0 | 0 |
| C32 | 2020-2024 | 0 | | 0 | 0 | 0 | 0 | 0 | 0 |
|  | 2025-2029 | 0 | | 0 | 0 | 0 | 0 | 0 | 0 |
|  | 2030-2034 | 0 | | 0 | 0 | 0 | 0 | 0 | 0 |
|  | 2035-2039 | 0 | | 0 | 0 | 0 | 0 | 0 | 0 |
| Lung | 2015-2019 | 0 | | 0 | 0 | 87 | 267 | 242 | 596 |
| C33-C34 | 2020-2024 | 0 | | 0 | 0 | 86 | 286 | 300 | 672 |
|  | 2025-2029 | 0 | | 0 | 0 | 90 | 239 | 370 | 698 |
|  | 2030-2034 | 0 | | 0 | 0 | 96 | 223 | 373 | 692 |
|  | 2035-2039 | 0 | | 0 | 0 | 92 | 226 | 358 | 677 |
| Skin | 2015-2019 | 0 | | 0 | 0 | 0 | 0 | 0 | 0 |
| C43-C44 | 2020-2024 | 0 | | 0 | 0 | 0 | 0 | 0 | 0 |
|  | 2025-2029 | 0 | | 0 | 0 | 0 | 0 | 0 | 0 |
|  | 2030-2034 | 0 | | 0 | 0 | 0 | 0 | 0 | 0 |
|  | 2035-2039 | 0 | | 0 | 0 | 0 | 0 | 0 | 0 |
| Breast | 2015-2019 | 0 | | 98 | 114 | 453 | 458 | 515 | 1,637 |
| C50 | 2020-2024 | 0 | | 88 | 127 | 513 | 521 | 712 | 1,962 |
|  | 2025-2029 | 0 | | 79 | 118 | 587 | 488 | 964 | 2,235 |
|  | 2030-2034 | 0 | | 71 | 96 | 602 | 507 | 1,061 | 2,337 |
|  | 2035-2039 | 0 | | 74 | 78 | 537 | 555 | 1,117 | 2,361 |
| Uterus | 2015-2019 | 0 | | 337 | 183 | 940 | 381 | 495 | 2,337 |
| C53-C55 | 2020-2024 | 0 | | 365 | 227 | 1,184 | 420 | 620 | 2,817 |
|  | 2025-2029 | 0 | | 372 | 243 | 1,513 | 416 | 790 | 3,333 |
|  | 2030-2034 | 0 | | 340 | 251 | 1,710 | 486 | 833 | 3,619 |
|  | 2035-2039 | 0 | | 344 | 237 | 1,739 | 599 | 890 | 3,808 |
| Ovary | 2015-2019 | 0 | | 99 | 284 | 404 | 164 | 211 | 1,163 |
| C56 | 2020-2024 | 0 | | 96 | 293 | 425 | 164 | 251 | 1,229 |
|  | 2025-2029 | 0 | | 95 | 272 | 465 | 141 | 310 | 1,284 |
|  | 2030-2034 | 0 | | 91 | 248 | 462 | 144 | 311 | 1,256 |
|  | 2035-2039 | 0 | | 90 | 233 | 421 | 155 | 300 | 1,200 |
| Urinary | 2015-2019 | 0 | | 0 | 0 | 0 | 0 | 221 | 221 |
| bladder | 2020-2024 | 0 | | 0 | 0 | 0 | 0 | 248 | 248 |
| C67 | 2025-2029 | 0 | | 0 | 0 | 0 | 0 | 287 | 287 |
|  | 2030-2034 | 0 | | 0 | 0 | 0 | 0 | 305 | 305 |
|  | 2035-2039 | 0 | | 0 | 0 | 0 | 0 | 318 | 318 |
| Kidney | 2015-2019 | 0 | | 0 | 117 | 0 | 366 | 334 | 818 |
| & ureter | 2020-2024 | 0 | | 0 | 139 | 0 | 419 | 400 | 959 |
| C64-C66, | 2025-2029 | 0 | | 0 | 129 | 0 | 360 | 488 | 977 |
| C68 | 2030-2034 | 0 | | 0 | 92 | 0 | 337 | 508 | 937 |
|  | 2035-2039 | 0 | | 0 | 54 | 0 | 360 | 509 | 924 |
| Thyroid | 2015-2019 | 0 | | 0 | 0 | 0 | 0 | 0 | 0 |
| gland | 2020-2024 | 0 | | 0 | 0 | 0 | 0 | 0 | 0 |
| C73 | 2025-2029 | 0 | | 0 | 0 | 0 | 0 | 0 | 0 |
|  | 2030-2034 | 0 | | 0 | 0 | 0 | 0 | 0 | 0 |
|  | 2035-2039 | 0 | | 0 | 0 | 0 | 0 | 0 | 0 |
| Lymphoma | 2015-2019 | 60 | | 206 | 0 | 327 | 461 | 0 | 1,054 |
| C81-C85, | 2020-2024 | 55 | | 203 | 0 | 321 | 491 | 0 | 1,070 |
| C96 | 2025-2029 | 53 | | 187 | 0 | 318 | 440 | 0 | 998 |
|  | 2030-2034 | 50 | | 175 | 0 | 342 | 410 | 0 | 977 |
|  | 2035-2039 | 48 | | 176 | 0 | 349 | 401 | 0 | 973 |
| Myeloma | 2015-2019 | 0 | | 0 | 0 | 0 | 221 | 0 | 221 |
| C88-C90 | 2020-2024 | 0 | | 0 | 0 | 0 | 232 | 0 | 232 |
|  | 2025-2029 | 0 | | 0 | 0 | 0 | 208 | 0 | 208 |
|  | 2030-2034 | 0 | | 0 | 0 | 0 | 219 | 0 | 219 |
|  | 2035-2039 | 0 | | 0 | 0 | 0 | 251 | 0 | 251 |
| Leukemia | 2015-2019 | 0 | | 0 | 0 | 0 | 215 | 1,033 | 1,248 |
| C91-C95 | 2020-2024 | 0 | | 0 | 0 | 0 | 219 | 1,187 | 1,406 |
|  | 2025-2029 | 0 | | 0 | 0 | 0 | 186 | 1,377 | 1,563 |
|  | 2030-2034 | 0 | | 0 | 0 | 0 | 178 | 1,373 | 1,551 |
|  | 2035-2039 | 0 | | 0 | 0 | 0 | 195 | 1,347 | 1,541 |
| Total | 2015-2019 | 60 | | 882 | 698 | 3,351 | 4,628 | 5,604 | 15,224 |
|  | 2020-2024 | 55 | | 883 | 787 | 3,638 | 4,857 | 6,652 | 16,871 |
|  | 2025-2029 | 53 | | 860 | 763 | 4,178 | 4,229 | 7,928 | 18,011 |
|  | 2030-2034 | 50 | | 805 | 686 | 4,456 | 4,198 | 8,129 | 18,324 |
|  | 2035-2039 | 48 | | 808 | 602 | 4,267 | 4,589 | 8,095 | 18,409 |
|  |  |  | |  |  |  |  |  |  |
| NT-proBNP > 900 pg/mL in male | | | |  |  |  |  |  |  |
| Sites | Year | Age groups | | | | | | | |
|  |  | 0-14 | | 15-44 | 45-54 | 55-64 | 65-74 | 75+ | Total |
| Oral cavity | 2015-2019 | 0 | | 0 | 0 | 0 | 326 | 701 | 1,028 |
| & pharynx | 2020-2024 | 0 | | 0 | 0 | 0 | 340 | 871 | 1,210 |
| C00-C14 | 2025-2029 | 0 | | 0 | 0 | 0 | 286 | 1,092 | 1,378 |
|  | 2030-2034 | 0 | | 0 | 0 | 0 | 273 | 1,083 | 1,355 |
|  | 2035-2039 | 0 | | 0 | 0 | 0 | 311 | 1,031 | 1,342 |
| Esophagus | 2015-2019 | 0 | | 0 | 0 | 169 | 644 | 1,098 | 1,910 |
| C15 | 2020-2024 | 0 | | 0 | 0 | 162 | 632 | 1,316 | 2,110 |
|  | 2025-2029 | 0 | | 0 | 0 | 180 | 533 | 1,602 | 2,316 |
|  | 2030-2034 | 0 | | 0 | 0 | 210 | 529 | 1,606 | 2,345 |
|  | 2035-2039 | 0 | | 0 | 0 | 207 | 602 | 1,530 | 2,338 |
| Stomach | 2015-2019 | 0 | | 0 | 0 | 297 | 939 | 4,535 | 5,771 |
| C16 | 2020-2024 | 0 | | 0 | 0 | 256 | 922 | 5,017 | 6,195 |
|  | 2025-2029 | 0 | | 0 | 0 | 250 | 733 | 5,780 | 6,763 |
|  | 2030-2034 | 0 | | 0 | 0 | 266 | 651 | 5,701 | 6,618 |
|  | 2035-2039 | 0 | | 0 | 0 | 256 | 653 | 5,288 | 6,197 |
| Colon | 2015-2019 | 0 | | 0 | 153 | 158 | 1,292 | 6,309 | 7,912 |
| & rectum | 2020-2024 | 0 | | 0 | 169 | 154 | 1,289 | 7,284 | 8,897 |
| C18-C20 | 2025-2029 | 0 | | 0 | 160 | 166 | 1,066 | 8,534 | 9,926 |
|  | 2030-2034 | 0 | | 0 | 136 | 178 | 1,027 | 8,474 | 9,815 |
|  | 2035-2039 | 0 | | 0 | 126 | 164 | 1,105 | 8,076 | 9,472 |
| Liver | 2015-2019 | 0 | | 0 | 0 | 0 | 0 | 4,175 | 4,175 |
| C22 | 2020-2024 | 0 | | 0 | 0 | 0 | 0 | 4,003 | 4,003 |
|  | 2025-2029 | 0 | | 0 | 0 | 0 | 0 | 4,025 | 4,025 |
|  | 2030-2034 | 0 | | 0 | 0 | 0 | 0 | 3,690 | 3,690 |
|  | 2035-2039 | 0 | | 0 | 0 | 0 | 0 | 3,194 | 3,194 |
| Gallbladder | 2015-2019 | 0 | | 0 | 0 | 0 | 0 | 829 | 829 |
| & bile duct | 2020-2024 | 0 | | 0 | 0 | 0 | 0 | 938 | 938 |
| C23-C24 | 2025-2029 | 0 | | 0 | 0 | 0 | 0 | 1,101 | 1,101 |
|  | 2030-2034 | 0 | | 0 | 0 | 0 | 0 | 1,099 | 1,099 |
|  | 2035-2039 | 0 | | 0 | 0 | 0 | 0 | 1,035 | 1,035 |
| Pancreas | 2015-2019 | 0 | | 0 | 0 | 197 | 438 | 0 | 636 |
| C25 | 2020-2024 | 0 | | 0 | 0 | 186 | 436 | 0 | 622 |
|  | 2025-2029 | 0 | | 0 | 0 | 196 | 353 | 0 | 549 |
|  | 2030-2034 | 0 | | 0 | 0 | 204 | 329 | 0 | 533 |
|  | 2035-2039 | 0 | | 0 | 0 | 189 | 349 | 0 | 538 |
| Larynx | 2015-2019 | 0 | | 0 | 0 | 79 | 0 | 0 | 79 |
| C32 | 2020-2024 | 0 | | 0 | 0 | 71 | 0 | 0 | 71 |
|  | 2025-2029 | 0 | | 0 | 0 | 77 | 0 | 0 | 77 |
|  | 2030-2034 | 0 | | 0 | 0 | 84 | 0 | 0 | 84 |
|  | 2035-2039 | 0 | | 0 | 0 | 95 | 0 | 0 | 95 |
| Lung | 2015-2019 | 0 | | 0 | 204 | 372 | 1,173 | 1,487 | 3,235 |
| C33-C34 | 2020-2024 | 0 | | 0 | 219 | 356 | 1,223 | 1,740 | 3,538 |
|  | 2025-2029 | 0 | | 0 | 206 | 369 | 1,026 | 2,105 | 3,707 |
|  | 2030-2034 | 0 | | 0 | 191 | 378 | 958 | 2,123 | 3,650 |
|  | 2035-2039 | 0 | | 0 | 176 | 349 | 995 | 2,005 | 3,524 |
| Skin | 2015-2019 | 0 | | 0 | 0 | 0 | 0 | 1,064 | 1,064 |
| C43-C44 | 2020-2024 | 0 | | 0 | 0 | 0 | 0 | 1,368 | 1,368 |
|  | 2025-2029 | 0 | | 0 | 0 | 0 | 0 | 1,677 | 1,677 |
|  | 2030-2034 | 0 | | 0 | 0 | 0 | 0 | 1,770 | 1,770 |
|  | 2035-2039 | 0 | | 0 | 0 | 0 | 0 | 1,834 | 1,834 |
| Prostate | 2015-2019 | 0 | | 0 | 0 | 117 | 1,465 | 2,042 | 3,624 |
| C61 | 2020-2024 | 0 | | 0 | 0 | 119 | 1,763 | 2,925 | 4,807 |
|  | 2025-2029 | 0 | | 0 | 0 | 115 | 1,650 | 4,061 | 5,825 |
|  | 2030-2034 | 0 | | 0 | 0 | 101 | 1,609 | 4,639 | 6,349 |
|  | 2035-2039 | 0 | | 0 | 0 | 90 | 1,559 | 4,879 | 6,527 |
| Urinary | 2015-2019 | 0 | | 0 | 0 | 0 | 211 | 802 | 1,013 |
| bladder | 2020-2024 | 0 | | 0 | 0 | 0 | 211 | 920 | 1,131 |
| C67 | 2025-2029 | 0 | | 0 | 0 | 0 | 167 | 1,105 | 1,272 |
|  | 2030-2034 | 0 | | 0 | 0 | 0 | 156 | 1,146 | 1,302 |
|  | 2035-2039 | 0 | | 0 | 0 | 0 | 155 | 1,109 | 1,264 |
| Kidney | 2015-2019 | 0 | | 0 | 0 | 248 | 517 | 0 | 765 |
| & ureter | 2020-2024 | 0 | | 0 | 0 | 276 | 604 | 0 | 880 |
| C64-C66, | 2025-2029 | 0 | | 0 | 0 | 332 | 556 | 0 | 888 |
| C68 | 2030-2034 | 0 | | 0 | 0 | 357 | 565 | 0 | 921 |
|  | 2035-2039 | 0 | | 0 | 0 | 316 | 648 | 0 | 964 |
| Thyroid | 2015-2019 | 0 | | 0 | 0 | 0 | 0 | 0 | 0 |
| gland | 2020-2024 | 0 | | 0 | 0 | 0 | 0 | 0 | 0 |
| C73 | 2025-2029 | 0 | | 0 | 0 | 0 | 0 | 0 | 0 |
|  | 2030-2034 | 0 | | 0 | 0 | 0 | 0 | 0 | 0 |
|  | 2035-2039 | 0 | | 0 | 0 | 0 | 0 | 0 | 0 |
| Lymphoma | 2015-2019 | 0 | | 0 | 171 | 150 | 0 | 780 | 1,101 |
| C81-C85, | 2020-2024 | 0 | | 0 | 203 | 160 | 0 | 972 | 1,335 |
| C96 | 2025-2029 | 0 | | 0 | 205 | 174 | 0 | 1,214 | 1,593 |
|  | 2030-2034 | 0 | | 0 | 185 | 186 | 0 | 1,290 | 1,661 |
|  | 2035-2039 | 0 | | 0 | 166 | 178 | 0 | 1,310 | 1,654 |
| Myeloma | 2015-2019 | 0 | | 0 | 0 | 240 | 187 | 566 | 992 |
| C88-C90 | 2020-2024 | 0 | | 0 | 0 | 239 | 198 | 676 | 1,112 |
|  | 2025-2029 | 0 | | 0 | 0 | 256 | 165 | 796 | 1,217 |
|  | 2030-2034 | 0 | | 0 | 0 | 269 | 151 | 791 | 1,211 |
|  | 2035-2039 | 0 | | 0 | 0 | 240 | 155 | 720 | 1,115 |
| Leukemia | 2015-2019 | 261 | | 186 | 0 | 281 | 675 | 470 | 1,873 |
| C91-C95 | 2020-2024 | 244 | | 180 | 0 | 290 | 688 | 539 | 1,941 |
|  | 2025-2029 | 228 | | 185 | 0 | 319 | 578 | 639 | 1,948 |
|  | 2030-2034 | 217 | | 174 | 0 | 363 | 572 | 630 | 1,956 |
|  | 2035-2039 | 207 | | 172 | 0 | 349 | 618 | 603 | 1,949 |
| Total | 2015-2019 | 261 | | 186 | 527 | 2,309 | 7,866 | 24,858 | 36,007 |
|  | 2020-2024 | 244 | | 180 | 592 | 2,268 | 8,306 | 28,569 | 40,159 |
|  | 2025-2029 | 228 | | 185 | 572 | 2,433 | 7,114 | 33,729 | 44,261 |
|  | 2030-2034 | 217 | | 174 | 512 | 2,596 | 6,819 | 34,041 | 44,359 |
|  | 2035-2039 | 207 | | 172 | 468 | 2,432 | 7,151 | 32,614 | 43,044 |
|  |  |  | |  |  |  |  |  |  |
|  |  |  | |  |  |  |  |  |  |
| NT-proBNP > 900 pg/mL in female | | | |  |  |  |  |  |  |
| Sites | Year | Age groups | | | | | | | |
|  |  | 0-14 | | 15-44 | 45-54 | 55-64 | 65-74 | 75+ | Total |
| Oral cavity | 2015-2019 | 0 | | 0 | 0 | 0 | 0 | 0 | 0 |
| & pharynx | 2020-2024 | 0 | | 0 | 0 | 0 | 0 | 0 | 0 |
| C00-C14 | 2025-2029 | 0 | | 0 | 0 | 0 | 0 | 0 | 0 |
|  | 2030-2034 | 0 | | 0 | 0 | 0 | 0 | 0 | 0 |
|  | 2035-2039 | 0 | | 0 | 0 | 0 | 0 | 0 | 0 |
| Esophagus | 2015-2019 | 0 | | 0 | 0 | 0 | 0 | 175 | 175 |
| C15 | 2020-2024 | 0 | | 0 | 0 | 0 | 0 | 205 | 205 |
|  | 2025-2029 | 0 | | 0 | 0 | 0 | 0 | 246 | 246 |
|  | 2030-2034 | 0 | | 0 | 0 | 0 | 0 | 255 | 255 |
|  | 2035-2039 | 0 | | 0 | 0 | 0 | 0 | 257 | 257 |
| Stomach | 2015-2019 | 0 | | 0 | 0 | 0 | 148 | 522 | 670 |
| C16 | 2020-2024 | 0 | | 0 | 0 | 0 | 146 | 569 | 716 |
|  | 2025-2029 | 0 | | 0 | 0 | 0 | 116 | 645 | 761 |
|  | 2030-2034 | 0 | | 0 | 0 | 0 | 104 | 648 | 752 |
|  | 2035-2039 | 0 | | 0 | 0 | 0 | 103 | 619 | 722 |
| Colon | 2015-2019 | 0 | | 0 | 0 | 162 | 552 | 2,552 | 3,266 |
| & rectum | 2020-2024 | 0 | | 0 | 0 | 160 | 554 | 2,935 | 3,649 |
| C18-C20 | 2025-2029 | 0 | | 0 | 0 | 177 | 459 | 3,343 | 3,979 |
|  | 2030-2034 | 0 | | 0 | 0 | 183 | 441 | 3,364 | 3,988 |
|  | 2035-2039 | 0 | | 0 | 0 | 166 | 480 | 3,255 | 3,900 |
| Liver | 2015-2019 | 0 | | 0 | 0 | 0 | 0 | 0 | 0 |
| C22 | 2020-2024 | 0 | | 0 | 0 | 0 | 0 | 0 | 0 |
|  | 2025-2029 | 0 | | 0 | 0 | 0 | 0 | 0 | 0 |
|  | 2030-2034 | 0 | | 0 | 0 | 0 | 0 | 0 | 0 |
|  | 2035-2039 | 0 | | 0 | 0 | 0 | 0 | 0 | 0 |
| Gallbladder | 2015-2019 | 0 | | 0 | 0 | 0 | 0 | 0 | 0 |
| & bile duct | 2020-2024 | 0 | | 0 | 0 | 0 | 0 | 0 | 0 |
| C23-C24 | 2025-2029 | 0 | | 0 | 0 | 0 | 0 | 0 | 0 |
|  | 2030-2034 | 0 | | 0 | 0 | 0 | 0 | 0 | 0 |
|  | 2035-2039 | 0 | | 0 | 0 | 0 | 0 | 0 | 0 |
| Pancreas | 2015-2019 | 0 | | 0 | 0 | 0 | 230 | 0 | 230 |
| C25 | 2020-2024 | 0 | | 0 | 0 | 0 | 245 | 0 | 245 |
|  | 2025-2029 | 0 | | 0 | 0 | 0 | 209 | 0 | 209 |
|  | 2030-2034 | 0 | | 0 | 0 | 0 | 197 | 0 | 197 |
|  | 2035-2039 | 0 | | 0 | 0 | 0 | 217 | 0 | 217 |
| Larynx | 2015-2019 | 0 | | 0 | 0 | 0 | 0 | 0 | 0 |
| C32 | 2020-2024 | 0 | | 0 | 0 | 0 | 0 | 0 | 0 |
|  | 2025-2029 | 0 | | 0 | 0 | 0 | 0 | 0 | 0 |
|  | 2030-2034 | 0 | | 0 | 0 | 0 | 0 | 0 | 0 |
|  | 2035-2039 | 0 | | 0 | 0 | 0 | 0 | 0 | 0 |
| Lung | 2015-2019 | 0 | | 0 | 0 | 87 | 534 | 0 | 621 |
| C33-C34 | 2020-2024 | 0 | | 0 | 0 | 86 | 572 | 0 | 658 |
|  | 2025-2029 | 0 | | 0 | 0 | 90 | 478 | 0 | 568 |
|  | 2030-2034 | 0 | | 0 | 0 | 96 | 445 | 0 | 541 |
|  | 2035-2039 | 0 | | 0 | 0 | 92 | 453 | 0 | 545 |
| Skin | 2015-2019 | 0 | | 0 | 0 | 0 | 0 | 0 | 0 |
| C43-C44 | 2020-2024 | 0 | | 0 | 0 | 0 | 0 | 0 | 0 |
|  | 2025-2029 | 0 | | 0 | 0 | 0 | 0 | 0 | 0 |
|  | 2030-2034 | 0 | | 0 | 0 | 0 | 0 | 0 | 0 |
|  | 2035-2039 | 0 | | 0 | 0 | 0 | 0 | 0 | 0 |
| Breast | 2015-2019 | 0 | | 98 | 114 | 226 | 1,145 | 2,059 | 3,642 |
| C50 | 2020-2024 | 0 | | 88 | 127 | 257 | 1,303 | 2,847 | 4,622 |
|  | 2025-2029 | 0 | | 79 | 118 | 293 | 1,219 | 3,857 | 5,566 |
|  | 2030-2034 | 0 | | 71 | 96 | 301 | 1,268 | 4,244 | 5,980 |
|  | 2035-2039 | 0 | | 74 | 78 | 268 | 1,389 | 4,469 | 6,278 |
| Uterus | 2015-2019 | 0 | | 0 | 0 | 104 | 572 | 495 | 1,171 |
| C53-C55 | 2020-2024 | 0 | | 0 | 0 | 132 | 631 | 620 | 1,382 |
|  | 2025-2029 | 0 | | 0 | 0 | 168 | 625 | 790 | 1,582 |
|  | 2030-2034 | 0 | | 0 | 0 | 190 | 728 | 833 | 1,751 |
|  | 2035-2039 | 0 | | 0 | 0 | 193 | 899 | 890 | 1,981 |
| Ovary | 2015-2019 | 0 | | 0 | 284 | 101 | 327 | 423 | 1,135 |
| C56 | 2020-2024 | 0 | | 0 | 293 | 106 | 329 | 502 | 1,230 |
|  | 2025-2029 | 0 | | 0 | 272 | 116 | 281 | 621 | 1,291 |
|  | 2030-2034 | 0 | | 0 | 248 | 115 | 288 | 623 | 1,274 |
|  | 2035-2039 | 0 | | 0 | 233 | 105 | 311 | 601 | 1,250 |
| Urinary | 2015-2019 | 0 | | 0 | 0 | 0 | 0 | 0 | 0 |
| bladder | 2020-2024 | 0 | | 0 | 0 | 0 | 0 | 0 | 0 |
| C67 | 2025-2029 | 0 | | 0 | 0 | 0 | 0 | 0 | 0 |
|  | 2030-2034 | 0 | | 0 | 0 | 0 | 0 | 0 | 0 |
|  | 2035-2039 | 0 | | 0 | 0 | 0 | 0 | 0 | 0 |
| Kidney | 2015-2019 | 0 | | 0 | 0 | 0 | 0 | 334 | 334 |
| & ureter | 2020-2024 | 0 | | 0 | 0 | 0 | 0 | 400 | 400 |
| C64-C66, | 2025-2029 | 0 | | 0 | 0 | 0 | 0 | 488 | 488 |
| C68 | 2030-2034 | 0 | | 0 | 0 | 0 | 0 | 508 | 508 |
|  | 2035-2039 | 0 | | 0 | 0 | 0 | 0 | 509 | 509 |
| Thyroid | 2015-2019 | 0 | | 0 | 0 | 0 | 0 | 0 | 0 |
| gland | 2020-2024 | 0 | | 0 | 0 | 0 | 0 | 0 | 0 |
| C73 | 2025-2029 | 0 | | 0 | 0 | 0 | 0 | 0 | 0 |
|  | 2030-2034 | 0 | | 0 | 0 | 0 | 0 | 0 | 0 |
|  | 2035-2039 | 0 | | 0 | 0 | 0 | 0 | 0 | 0 |
| Lymphoma | 2015-2019 | 0 | | 0 | 0 | 109 | 461 | 1,831 | 2,401 |
| C81-C85, | 2020-2024 | 0 | | 0 | 0 | 107 | 491 | 2,159 | 2,757 |
| C96 | 2025-2029 | 0 | | 0 | 0 | 106 | 440 | 2,553 | 3,100 |
|  | 2030-2034 | 0 | | 0 | 0 | 114 | 410 | 2,622 | 3,146 |
|  | 2035-2039 | 0 | | 0 | 0 | 116 | 401 | 2,603 | 3,120 |
| Myeloma | 2015-2019 | 0 | | 0 | 0 | 0 | 0 | 1,948 | 1,948 |
| C88-C90 | 2020-2024 | 0 | | 0 | 0 | 0 | 0 | 2,340 | 2,340 |
|  | 2025-2029 | 0 | | 0 | 0 | 0 | 0 | 2,720 | 2,720 |
|  | 2030-2034 | 0 | | 0 | 0 | 0 | 0 | 2,800 | 2,800 |
|  | 2035-2039 | 0 | | 0 | 0 | 0 | 0 | 2,724 | 2,724 |
| Leukemia | 2015-2019 | 109 | | 0 | 0 | 0 | 215 | 517 | 840 |
| C91-C95 | 2020-2024 | 102 | | 0 | 0 | 0 | 219 | 593 | 915 |
|  | 2025-2029 | 95 | | 0 | 0 | 0 | 186 | 688 | 969 |
|  | 2030-2034 | 90 | | 0 | 0 | 0 | 178 | 687 | 954 |
|  | 2035-2039 | 86 | | 0 | 0 | 0 | 195 | 673 | 954 |
| Total | 2015-2019 | 109 | | 98 | 398 | 790 | 4,184 | 10,856 | 16,435 |
|  | 2020-2024 | 102 | | 88 | 420 | 847 | 4,489 | 13,171 | 19,117 |
|  | 2025-2029 | 95 | | 79 | 390 | 950 | 4,014 | 15,950 | 21,478 |
|  | 2030-2034 | 90 | | 71 | 343 | 999 | 4,060 | 16,582 | 22,146 |
|  | 2035-2039 | 86 | | 74 | 311 | 940 | 4,446 | 16,599 | 22,457 |
|  |  |  | |  |  |  |  |  |  |
| Cardiovascular disease in male | | | |  |  |  |  |  |  |
| Sites | Year | Age groups | | | | | | | |
|  |  | 0-14 | | 15-44 | 45-54 | 55-64 | 65-74 | 75+ | Total |
| Oral cavity | 2015-2019 | 0 | | 0 | 0 | 973 | 2,285 | 2,104 | 5,362 |
| & pharynx | 2020-2024 | 0 | | 0 | 0 | 973 | 2,379 | 2,612 | 5,963 |
| C00-C14 | 2025-2029 | 0 | | 0 | 0 | 1,131 | 2,002 | 3,276 | 6,409 |
|  | 2030-2034 | 0 | | 0 | 0 | 1,285 | 1,908 | 3,248 | 6,441 |
|  | 2035-2039 | 0 | | 0 | 0 | 1,178 | 2,178 | 3,092 | 6,448 |
| Esophagus | 2015-2019 | 0 | | 0 | 294 | 1,433 | 3,090 | 2,854 | 7,672 |
| C15 | 2020-2024 | 0 | | 0 | 347 | 1,374 | 3,034 | 3,421 | 8,177 |
|  | 2025-2029 | 0 | | 0 | 352 | 1,531 | 2,560 | 4,166 | 8,609 |
|  | 2030-2034 | 0 | | 0 | 299 | 1,786 | 2,541 | 4,176 | 8,801 |
|  | 2035-2039 | 0 | | 0 | 218 | 1,756 | 2,890 | 3,977 | 8,841 |
| Stomach | 2015-2019 | 0 | | 0 | 370 | 3,363 | 11,075 | 15,226 | 30,034 |
| C16 | 2020-2024 | 0 | | 0 | 393 | 2,905 | 10,879 | 16,842 | 31,019 |
|  | 2025-2029 | 0 | | 0 | 382 | 2,832 | 8,653 | 19,405 | 31,272 |
|  | 2030-2034 | 0 | | 0 | 341 | 3,015 | 7,677 | 19,141 | 30,173 |
|  | 2035-2039 | 0 | | 0 | 300 | 2,902 | 7,703 | 17,753 | 28,658 |
| Colon | 2015-2019 | 0 | | 283 | 1,684 | 3,168 | 12,921 | 16,298 | 34,353 |
| & rectum | 2020-2024 | 0 | | 251 | 1,864 | 3,083 | 12,887 | 18,818 | 36,903 |
| C18-C20 | 2025-2029 | 0 | | 244 | 1,762 | 3,320 | 10,659 | 22,046 | 38,030 |
|  | 2030-2034 | 0 | | 218 | 1,499 | 3,552 | 10,271 | 21,891 | 37,430 |
|  | 2035-2039 | 0 | | 218 | 1,387 | 3,285 | 11,052 | 20,863 | 36,805 |
| Liver | 2015-2019 | 0 | | 0 | 0 | 696 | 4,172 | 6,958 | 11,825 |
| C22 | 2020-2024 | 0 | | 0 | 0 | 588 | 3,655 | 6,671 | 10,914 |
|  | 2025-2029 | 0 | | 0 | 0 | 599 | 2,723 | 6,708 | 10,030 |
|  | 2030-2034 | 0 | | 0 | 0 | 642 | 2,440 | 6,150 | 9,232 |
|  | 2035-2039 | 0 | | 0 | 0 | 605 | 2,570 | 5,324 | 8,498 |
| Gallbladder | 2015-2019 | 0 | | 0 | 0 | 140 | 444 | 2,073 | 2,656 |
| & bile duct | 2020-2024 | 0 | | 0 | 0 | 127 | 444 | 2,346 | 2,917 |
| C23-C24 | 2025-2029 | 0 | | 0 | 0 | 137 | 352 | 2,752 | 3,240 |
|  | 2030-2034 | 0 | | 0 | 0 | 153 | 321 | 2,748 | 3,222 |
|  | 2035-2039 | 0 | | 0 | 0 | 147 | 349 | 2,588 | 3,084 |
| Pancreas | 2015-2019 | 0 | | 55 | 305 | 592 | 1,315 | 1,500 | 3,767 |
| C25 | 2020-2024 | 0 | | 50 | 328 | 557 | 1,308 | 1,786 | 4,029 |
|  | 2025-2029 | 0 | | 37 | 310 | 587 | 1,060 | 2,151 | 4,144 |
|  | 2030-2034 | 0 | | 30 | 263 | 612 | 988 | 2,105 | 3,997 |
|  | 2035-2039 | 0 | | 30 | 190 | 568 | 1,047 | 1,934 | 3,769 |
| Larynx | 2015-2019 | 0 | | 0 | 0 | 396 | 1,283 | 216 | 1,894 |
| C32 | 2020-2024 | 0 | | 0 | 0 | 353 | 1,226 | 248 | 1,828 |
|  | 2025-2029 | 0 | | 0 | 0 | 383 | 980 | 288 | 1,652 |
|  | 2030-2034 | 0 | | 0 | 0 | 419 | 877 | 277 | 1,573 |
|  | 2035-2039 | 0 | | 0 | 0 | 474 | 969 | 249 | 1,692 |
| Lung | 2015-2019 | 0 | | 155 | 408 | 2,008 | 8,065 | 7,433 | 18,069 |
| C33-C34 | 2020-2024 | 0 | | 161 | 438 | 1,920 | 8,408 | 8,701 | 19,629 |
|  | 2025-2029 | 0 | | 159 | 413 | 1,994 | 7,056 | 10,525 | 20,147 |
|  | 2030-2034 | 0 | | 156 | 381 | 2,042 | 6,589 | 10,614 | 19,782 |
|  | 2035-2039 | 0 | | 159 | 351 | 1,882 | 6,841 | 10,026 | 19,259 |
| Skin | 2015-2019 | 0 | | 0 | 0 | 189 | 708 | 2,660 | 3,557 |
| C43-C44 | 2020-2024 | 0 | | 0 | 0 | 235 | 830 | 3,421 | 4,486 |
|  | 2025-2029 | 0 | | 0 | 0 | 298 | 774 | 4,193 | 5,265 |
|  | 2030-2034 | 0 | | 0 | 0 | 362 | 825 | 4,425 | 5,612 |
|  | 2035-2039 | 0 | | 0 | 0 | 354 | 967 | 4,586 | 5,907 |
| Prostate | 2015-2019 | 0 | | 0 | 0 | 1,525 | 14,160 | 20,416 | 36,101 |
| C61 | 2020-2024 | 0 | | 0 | 0 | 1,549 | 17,042 | 29,254 | 47,845 |
|  | 2025-2029 | 0 | | 0 | 0 | 1,493 | 15,950 | 40,605 | 58,048 |
|  | 2030-2034 | 0 | | 0 | 0 | 1,314 | 15,556 | 46,385 | 63,256 |
|  | 2035-2039 | 0 | | 0 | 0 | 1,164 | 15,071 | 48,786 | 65,021 |
| Urinary | 2015-2019 | 0 | | 0 | 227 | 610 | 2,636 | 5,935 | 9,408 |
| bladder | 2020-2024 | 0 | | 0 | 204 | 562 | 2,637 | 6,805 | 10,208 |
| C67 | 2025-2029 | 0 | | 0 | 171 | 546 | 2,087 | 8,174 | 10,978 |
|  | 2030-2034 | 0 | | 0 | 143 | 507 | 1,947 | 8,478 | 11,076 |
|  | 2035-2039 | 0 | | 0 | 118 | 435 | 1,933 | 8,209 | 10,695 |
| Kidney | 2015-2019 | 0 | | 0 | 255 | 1,613 | 2,928 | 1,506 | 6,303 |
| & ureter | 2020-2024 | 0 | | 0 | 311 | 1,794 | 3,425 | 1,904 | 7,434 |
| C64-C66, | 2025-2029 | 0 | | 0 | 289 | 2,155 | 3,151 | 2,475 | 8,070 |
| C68 | 2030-2034 | 0 | | 0 | 216 | 2,318 | 3,199 | 2,624 | 8,357 |
|  | 2035-2039 | 0 | | 0 | 158 | 2,052 | 3,674 | 2,627 | 8,512 |
| Thyroid | 2015-2019 | 0 | | 0 | 98 | 339 | 476 | 0 | 914 |
| gland | 2020-2024 | 0 | | 0 | 127 | 393 | 546 | 0 | 1,066 |
| C73 | 2025-2029 | 0 | | 0 | 130 | 491 | 499 | 0 | 1,120 |
|  | 2030-2034 | 0 | | 0 | 114 | 563 | 525 | 0 | 1,201 |
|  | 2035-2039 | 0 | | 0 | 117 | 546 | 625 | 0 | 1,288 |
| Lymphoma | 2015-2019 | 0 | | 173 | 512 | 1,053 | 2,268 | 2,341 | 6,347 |
| C81-C85, | 2020-2024 | 0 | | 182 | 609 | 1,120 | 2,608 | 2,916 | 7,436 |
| C96 | 2025-2029 | 0 | | 184 | 616 | 1,216 | 2,489 | 3,641 | 8,147 |
|  | 2030-2034 | 0 | | 180 | 555 | 1,304 | 2,442 | 3,869 | 8,350 |
|  | 2035-2039 | 0 | | 184 | 498 | 1,248 | 2,582 | 3,930 | 8,442 |
| Myeloma | 2015-2019 | 0 | | 0 | 0 | 600 | 933 | 566 | 2,098 |
| C88-C90 | 2020-2024 | 0 | | 0 | 0 | 597 | 990 | 676 | 2,262 |
|  | 2025-2029 | 0 | | 0 | 0 | 640 | 825 | 796 | 2,261 |
|  | 2030-2034 | 0 | | 0 | 0 | 673 | 753 | 791 | 2,217 |
|  | 2035-2039 | 0 | | 0 | 0 | 600 | 775 | 720 | 2,095 |
| Leukemia | 2015-2019 | 287 | | 186 | 298 | 422 | 675 | 470 | 2,338 |
| C91-C95 | 2020-2024 | 269 | | 180 | 362 | 435 | 688 | 539 | 2,472 |
|  | 2025-2029 | 251 | | 185 | 358 | 478 | 578 | 639 | 2,489 |
|  | 2030-2034 | 238 | | 174 | 325 | 545 | 572 | 630 | 2,484 |
|  | 2035-2039 | 228 | | 172 | 322 | 523 | 618 | 603 | 2,466 |
| Total | 2015-2019 | 287 | | 851 | 4,451 | 19,119 | 69,434 | 88,555 | 182,697 |
|  | 2020-2024 | 269 | | 824 | 4,982 | 18,565 | 72,988 | 106,960 | 204,587 |
|  | 2025-2029 | 251 | | 807 | 4,784 | 19,832 | 62,398 | 131,840 | 219,912 |
|  | 2030-2034 | 238 | | 758 | 4,136 | 21,091 | 59,429 | 137,552 | 223,204 |
|  | 2035-2039 | 228 | | 763 | 3,659 | 19,719 | 61,845 | 135,267 | 221,481 |
|  |  |  | |  |  |  |  |  |  |
| Cardiovascular disease in female | | | |  |  |  |  |  |  |
| Sites | Year | Age groups | | | | | | | |
|  |  | 0-14 | | 15-44 | 45-54 | 55-64 | 65-74 | 75+ | Total |
| Oral cavity | 2015-2019 | 0 | | 0 | 0 | 0 | 0 | 0 | 0 |
| & pharynx | 2020-2024 | 0 | | 0 | 0 | 0 | 0 | 0 | 0 |
| C00-C14 | 2025-2029 | 0 | | 0 | 0 | 0 | 0 | 0 | 0 |
|  | 2030-2034 | 0 | | 0 | 0 | 0 | 0 | 0 | 0 |
|  | 2035-2039 | 0 | | 0 | 0 | 0 | 0 | 0 | 0 |
| Esophagus | 2015-2019 | 0 | | 0 | 0 | 0 | 490 | 524 | 1,014 |
| C15 | 2020-2024 | 0 | | 0 | 0 | 0 | 496 | 615 | 1,111 |
|  | 2025-2029 | 0 | | 0 | 0 | 0 | 429 | 737 | 1,165 |
|  | 2030-2034 | 0 | | 0 | 0 | 0 | 455 | 765 | 1,220 |
|  | 2035-2039 | 0 | | 0 | 0 | 0 | 503 | 770 | 1,273 |
| Stomach | 2015-2019 | 0 | | 141 | 0 | 169 | 1,036 | 3,395 | 4,740 |
| C16 | 2020-2024 | 0 | | 130 | 0 | 149 | 1,023 | 3,702 | 5,003 |
|  | 2025-2029 | 0 | | 128 | 0 | 146 | 810 | 4,192 | 5,275 |
|  | 2030-2034 | 0 | | 127 | 0 | 147 | 729 | 4,210 | 5,213 |
|  | 2035-2039 | 0 | | 124 | 0 | 137 | 724 | 4,020 | 5,005 |
| Colon | 2015-2019 | 0 | | 0 | 0 | 1,296 | 4,970 | 9,571 | 15,837 |
| & rectum | 2020-2024 | 0 | | 0 | 0 | 1,278 | 4,985 | 11,006 | 17,269 |
| C18-C20 | 2025-2029 | 0 | | 0 | 0 | 1,413 | 4,134 | 12,538 | 18,085 |
|  | 2030-2034 | 0 | | 0 | 0 | 1,464 | 3,968 | 12,615 | 18,047 |
|  | 2035-2039 | 0 | | 0 | 0 | 1,325 | 4,316 | 12,206 | 17,846 |
| Liver | 2015-2019 | 0 | | 0 | 0 | 0 | 1,818 | 0 | 1,818 |
| C22 | 2020-2024 | 0 | | 0 | 0 | 0 | 1,602 | 0 | 1,602 |
|  | 2025-2029 | 0 | | 0 | 0 | 0 | 1,212 | 0 | 1,212 |
|  | 2030-2034 | 0 | | 0 | 0 | 0 | 1,112 | 0 | 1,112 |
|  | 2035-2039 | 0 | | 0 | 0 | 0 | 1,183 | 0 | 1,183 |
| Gallbladder | 2015-2019 | 0 | | 0 | 0 | 0 | 209 | 842 | 1,050 |
| & bile duct | 2020-2024 | 0 | | 0 | 0 | 0 | 200 | 884 | 1,085 |
| C23-C24 | 2025-2029 | 0 | | 0 | 0 | 0 | 158 | 938 | 1,097 |
|  | 2030-2034 | 0 | | 0 | 0 | 0 | 137 | 908 | 1,044 |
|  | 2035-2039 | 0 | | 0 | 0 | 0 | 135 | 854 | 989 |
| Pancreas | 2015-2019 | 0 | | 0 | 0 | 188 | 230 | 0 | 418 |
| C25 | 2020-2024 | 0 | | 0 | 0 | 187 | 245 | 0 | 432 |
|  | 2025-2029 | 0 | | 0 | 0 | 213 | 209 | 0 | 422 |
|  | 2030-2034 | 0 | | 0 | 0 | 231 | 197 | 0 | 428 |
|  | 2035-2039 | 0 | | 0 | 0 | 206 | 217 | 0 | 423 |
| Larynx | 2015-2019 | 0 | | 0 | 0 | 107 | 75 | 0 | 182 |
| C32 | 2020-2024 | 0 | | 0 | 0 | 87 | 70 | 0 | 157 |
|  | 2025-2029 | 0 | | 0 | 0 | 93 | 75 | 0 | 168 |
|  | 2030-2034 | 0 | | 0 | 0 | 113 | 67 | 0 | 180 |
|  | 2035-2039 | 0 | | 0 | 0 | 107 | 75 | 0 | 182 |
| Lung | 2015-2019 | 0 | | 0 | 93 | 434 | 2,271 | 2,182 | 4,980 |
| C33-C34 | 2020-2024 | 0 | | 0 | 109 | 430 | 2,431 | 2,698 | 5,667 |
|  | 2025-2029 | 0 | | 0 | 111 | 448 | 2,032 | 3,328 | 5,919 |
|  | 2030-2034 | 0 | | 0 | 89 | 478 | 1,893 | 3,360 | 5,819 |
|  | 2035-2039 | 0 | | 0 | 84 | 459 | 1,925 | 3,226 | 5,694 |
| Skin | 2015-2019 | 0 | | 0 | 0 | 93 | 385 | 517 | 996 |
| C43-C44 | 2020-2024 | 0 | | 0 | 0 | 112 | 445 | 669 | 1,226 |
|  | 2025-2029 | 0 | | 0 | 0 | 133 | 411 | 808 | 1,352 |
|  | 2030-2034 | 0 | | 0 | 0 | 151 | 431 | 879 | 1,461 |
|  | 2035-2039 | 0 | | 0 | 0 | 165 | 479 | 943 | 1,586 |
| Breast | 2015-2019 | 0 | | 489 | 571 | 1,812 | 7,555 | 7,206 | 17,632 |
| C50 | 2020-2024 | 0 | | 442 | 637 | 2,054 | 8,598 | 9,965 | 21,696 |
|  | 2025-2029 | 0 | | 393 | 590 | 2,346 | 8,049 | 13,499 | 24,877 |
|  | 2030-2034 | 0 | | 357 | 478 | 2,406 | 8,371 | 14,853 | 26,464 |
|  | 2035-2039 | 0 | | 369 | 391 | 2,146 | 9,166 | 15,641 | 27,713 |
| Uterus | 2015-2019 | 0 | | 675 | 0 | 1,358 | 1,524 | 2,229 | 5,786 |
| C53-C55 | 2020-2024 | 0 | | 730 | 0 | 1,710 | 1,681 | 2,791 | 6,913 |
|  | 2025-2029 | 0 | | 743 | 0 | 2,186 | 1,665 | 3,553 | 8,147 |
|  | 2030-2034 | 0 | | 679 | 0 | 2,471 | 1,942 | 3,748 | 8,839 |
|  | 2035-2039 | 0 | | 689 | 0 | 2,511 | 2,397 | 4,003 | 9,599 |
| Ovary | 2015-2019 | 0 | | 99 | 663 | 808 | 818 | 634 | 3,023 |
| C56 | 2020-2024 | 0 | | 96 | 683 | 850 | 822 | 753 | 3,204 |
|  | 2025-2029 | 0 | | 95 | 636 | 930 | 703 | 931 | 3,295 |
|  | 2030-2034 | 0 | | 91 | 578 | 924 | 720 | 934 | 3,248 |
|  | 2035-2039 | 0 | | 90 | 544 | 842 | 777 | 901 | 3,154 |
| Urinary | 2015-2019 | 0 | | 0 | 0 | 0 | 190 | 883 | 1,072 |
| bladder | 2020-2024 | 0 | | 0 | 0 | 0 | 206 | 992 | 1,198 |
| C67 | 2025-2029 | 0 | | 0 | 0 | 0 | 170 | 1,148 | 1,318 |
|  | 2030-2034 | 0 | | 0 | 0 | 0 | 165 | 1,221 | 1,386 |
|  | 2035-2039 | 0 | | 0 | 0 | 0 | 177 | 1,273 | 1,451 |
| Kidney | 2015-2019 | 0 | | 0 | 117 | 245 | 610 | 1,003 | 1,975 |
| & ureter | 2020-2024 | 0 | | 0 | 139 | 250 | 699 | 1,200 | 2,288 |
| C64-C66, | 2025-2029 | 0 | | 0 | 129 | 278 | 600 | 1,463 | 2,470 |
| C68 | 2030-2034 | 0 | | 0 | 92 | 294 | 561 | 1,524 | 2,471 |
|  | 2035-2039 | 0 | | 0 | 54 | 260 | 600 | 1,528 | 2,443 |
| Thyroid | 2015-2019 | 0 | | 0 | 0 | 0 | 210 | 432 | 641 |
| gland | 2020-2024 | 0 | | 0 | 0 | 0 | 215 | 546 | 761 |
| C73 | 2025-2029 | 0 | | 0 | 0 | 0 | 190 | 679 | 869 |
|  | 2030-2034 | 0 | | 0 | 0 | 0 | 194 | 683 | 877 |
|  | 2035-2039 | 0 | | 0 | 0 | 0 | 219 | 664 | 883 |
| Lymphoma | 2015-2019 | 0 | | 103 | 0 | 653 | 1,153 | 2,441 | 4,350 |
| C81-C85, | 2020-2024 | 0 | | 102 | 0 | 643 | 1,227 | 2,878 | 4,850 |
| C96 | 2025-2029 | 0 | | 94 | 0 | 637 | 1,101 | 3,404 | 5,235 |
|  | 2030-2034 | 0 | | 88 | 0 | 684 | 1,025 | 3,496 | 5,292 |
|  | 2035-2039 | 0 | | 88 | 0 | 698 | 1,002 | 3,471 | 5,259 |
| Myeloma | 2015-2019 | 0 | | 0 | 0 | 173 | 441 | 1,948 | 2,563 |
| C88-C90 | 2020-2024 | 0 | | 0 | 0 | 202 | 464 | 2,340 | 3,006 |
|  | 2025-2029 | 0 | | 0 | 0 | 247 | 416 | 2,720 | 3,383 |
|  | 2030-2034 | 0 | | 0 | 0 | 247 | 439 | 2,800 | 3,485 |
|  | 2035-2039 | 0 | | 0 | 0 | 260 | 503 | 2,724 | 3,487 |
| Leukemia | 2015-2019 | 254 | | 302 | 0 | 0 | 429 | 1,550 | 2,536 |
| C91-C95 | 2020-2024 | 238 | | 308 | 0 | 0 | 438 | 1,780 | 2,764 |
|  | 2025-2029 | 222 | | 326 | 0 | 0 | 372 | 2,065 | 2,985 |
|  | 2030-2034 | 210 | | 327 | 0 | 0 | 355 | 2,060 | 2,952 |
|  | 2035-2039 | 201 | | 322 | 0 | 0 | 389 | 2,020 | 2,932 |
| Total | 2015-2019 | 254 | | 1,809 | 1,443 | 7,336 | 24,415 | 35,356 | 70,614 |
|  | 2020-2024 | 238 | | 1,807 | 1,569 | 7,952 | 25,848 | 42,817 | 80,231 |
|  | 2025-2029 | 222 | | 1,778 | 1,467 | 9,068 | 22,736 | 52,002 | 87,273 |
|  | 2030-2034 | 210 | | 1,669 | 1,237 | 9,610 | 22,760 | 54,054 | 89,539 |
|  | 2035-2039 | 201 | | 1,682 | 1,073 | 9,115 | 24,786 | 54,244 | 91,101 |
